# Evaluation of the Role of ITGBL1 in Ovarian Cancer

**DOI:** 10.3390/cancers12092676

**Published:** 2020-09-19

**Authors:** Alexander Jorge Cortez, Katarzyna Aleksandra Kujawa, Agata Małgorzata Wilk, Damian Robert Sojka, Joanna Patrycja Syrkis, Magdalena Olbryt, Katarzyna Marta Lisowska

**Affiliations:** 1 Department of Biostatistics and Bioinformatics, Maria Skłodowska-Curie National Research Institute of Oncology, Gliwice Branch, 44-102 Gliwice, Poland; Alexander.Cortez@io.gliwice.pl (A.J.C.); Agata.Wilk@io.gliwice.pl (A.M.W.); 2 Center for Translational Research and Molecular Biology of Cancer, Maria Skłodowska-Curie National Research Institute of Oncology, Gliwice Branch, 44-102 Gliwice, Poland; Katarzyna.Kujawa@io.gliwice.pl (K.A.K.); Damian.Sojka@io.gliwice.pl (D.R.S.); Joanna.Syrkis@io.gliwice.pl (J.P.S.); Magdalena.Olbryt@io.gliwice.pl (M.O.)

**Keywords:** ITGBL1, TIED, ovarian cancer, functional genomics, gene expression profiling, migration, invasion, adhesion, proliferation, chemoresistance, signaling pathways

## Abstract

**Simple Summary:**

A poorly characterized protein called Integrin beta-like 1 (ITGBL1) may play an important role in ovarian cancer progression. Our previous studies have indicated that increased expression of the gene coding for ITGBL1 is related to poor prognosis for ovarian cancer patients. In the present study we investigated the role of ITGBL1 in ovarian cancer cells using several in-vitro assays and global gene expression analysis. We found that ITGBL1 overexpression affected cellular adhesion, motility and invasiveness. In addition, ITGBL1 caused cells to be more resistant to cisplatin and paclitaxel, major drugs used in ovarian cancer treatment. Our results indicate that higher expression of ITGBL1 in ovarian cancer is associated with the features that may worsen the clinical course of the disease. Further studies will show if ITGBL1 can be exploited as a cancer biomarker and/or molecular target for experimental biological therapy.

**Abstract:**

In our previous microarray study we identified two subgroups of high-grade serous ovarian cancers with distinct gene expression and survival. Among differentially expressed genes was an *Integrin beta-like 1* (*ITGBL1*), coding for a poorly characterized protein comprised of ten EGF-like repeats. Here, we have analyzed the influence of ITGBL1 on the phenotype of ovarian cancer (OC) cells. We analyzed expression of four putative *ITGBL1* mRNA isoforms in five OC cell lines. OAW42 and SKOV3, having the lowest level of any *ITGBL1* mRNA, were chosen to produce *ITGBL1*-overexpressing variants. In these cells, abundant *ITGBL1* mRNA expression could be detected by RT-PCR. Immunodetection was successful only in the culture media, suggesting that ITGBL1 is efficiently secreted. We found that *ITGBL1* overexpression affected cellular adhesion, migration and invasiveness, while it had no effect on proliferation rate and the cell cycle. *ITGBL1*-overexpressing cells were significantly more resistant to cisplatin and paclitaxel, major drugs used in OC treatment. Global gene expression analysis revealed that signaling pathways affected by *ITGBL1* overexpression were mostly those related to extracellular matrix organization and function, integrin signaling, focal adhesion, cellular communication and motility; these results were consistent with the findings of our functional studies. Overall, our results indicate that higher expression of *ITGBL1* in OC is associated with features that may worsen clinical course of the disease.

## 1. Introduction

Ovarian cancer is the second most common gynecologic malignancy in the western world. The disease is usually diagnosed late due to asymptomatic development and a lack of effective tools for early detection and screening. Advances in the diagnosis and treatment of ovarian cancer require further studies that will enable better understanding of its biology, empowering development of new molecular biomarkers and identification of possible new therapeutic targets [1,2].

In our previous microarray study we analyzed gene expression profiles of over 100 ovarian cancer samples [3]. We identified two molecular subgroups of high grade serous ovarian cancers (HG-SOC) with distinct gene expression profiles and survival rate [4]. *Integrin beta-like1* (*ITGBL1*) was one of the top ranked genes (rank 6 in 96-gene list) of the negative prognostic signature identified in that study; it demonstrated Fold Change = 8.1 between a low-expressing group (good prognosis) and a high-expressing group (bad prognosis). Also, currently available public algorithms like Kaplan-Meyer Plotter [5] and CSIOVDB database [6], operating on considerably larger sample sets, return significant results indicating a correlation between *ITGBL1* mRNA expression level in the tumor, as well as the survival of ovarian cancer patients.

ITGBL1 is a poorly characterized protein, with some structural similarity to integrin β. *ITGBL1* cDNA was first cloned in 1999 from the osteoblast cDNA library [7]. It was initially called TIED (Ten integrin EGF-like repeat domain-containing protein), the name precisely illustrating the structure of this protein. The present name, ITGBL1, refers to the fact that its EGF-like repeats show high amino-acid sequence homology compared to those found in integrins β, and have the same predicted fold [8]. However, integrins β have only four such repeats, while ITGBL1 has ten of them. Similar to integrins, ITGBL1 contains the signal peptide (SP), which can drive either protein translocation to the cellular membrane or its secretion. However, lack of transmembrane domain indicates a secretory nature of ITGBL1, as opposed to integrins which are membrane-anchored proteins. Moreover, unlike integrins, ITGBL1 contains neither a cytoplasmic domain nor a globular RGD (Arg–Gly–Asp) domain responsible for interactions with ECM molecules [7]. As a consequence, prediction of the functional properties of ITGBL1 should focus on those provided by ten EGF-like repeat domains.

EGF-like domains are present in numerous growth factors, receptors and adhesion molecules. They are found predominantly in soluble and cell surface proteins that mediate specific protein-protein recognition events [9]. Thus, it can be speculated that ITGBL1 may influence cellular adhesiveness and related properties like cellular motility and invasiveness, but it can also display growth factor activity [10,11].

Since its cloning and initial characterization in 1999, ITGBL1 studies have been abandoned for many years. We were the first to report in 2013 that *ITGBL1* overexpression stimulates ovarian cancer cell migration rate [12]. Later, since 2015, there have arisen a dozen or so reports concerning the ITGBL1 role in several human diseases, e.g., breast cancer [13], non-small cell lung cancer (NSCLC) [14,15], HBV-related liver pathologies [16,17], pulmonary fibrosis [15], osteoarthritis [18], colorectal [19,20,21], gastric [22], prostate [23] and ovarian cancer [24,25].

Based on the results of these studies, the role of ITGBL1 seems ambiguous (summarized in Supplementary Material 1). In liver [16,17] and pulmonary [15] pathologies, higher expression of *ITGBL1* was found to be correlated with severity of disease and its more advanced stage. Also, in the majority of solid tumors that were studied, a higher expression of *ITGBL1* was found to be associated with a more advanced stage, with the presence of distant metastases, worse prognosis, and/or with chemoresistance, indicating oncogenic properties of ITGBL1 [13,19,20,21,22,23,24,25]. The exception is NSCLC, in which ITGBL1 is postulated to play an opposite role of tumor suppressor, and its decreased level is associated with worse disease course [14]. Also, in acute myeloid leukemia, *ITGBL1* promoter hypermethylation (resulting in decreased protein level), is associated with worse prognosis [26].

Interestingly, it was experimentally established that ITGBL1 secreted from developing chondrocytes can physically interact with integrins to down-regulate their activity [18]. However, the study on ITGBL1′s role in colorectal cancer metastasis demonstrated no such interactions [20]. Thus, it seems that the role and activity of ITGBL1 may be diverse, both in physiology and in different pathological states.

In our current study we investigated ITGBL1 influence on ovarian cancer cells phenotype. Using various functional assays we found that *ITGBL1* overexpressing cells have altered adhesiveness and increased invasiveness and migration rate; however there is no change in proliferation rate and cell cycle. We also showed that cell lines overexpressing *ITGBL1* were more resistant to cisplatin and paclitaxel than control lines. These results were supported by signaling pathway analysis in cells with *ITGBL1* overexpression.

## 2. Results

### 2.1. ITGBL1 mRNA Expression in Ovarian Cancer Tissues and Cell Lines

**Figure 1:**
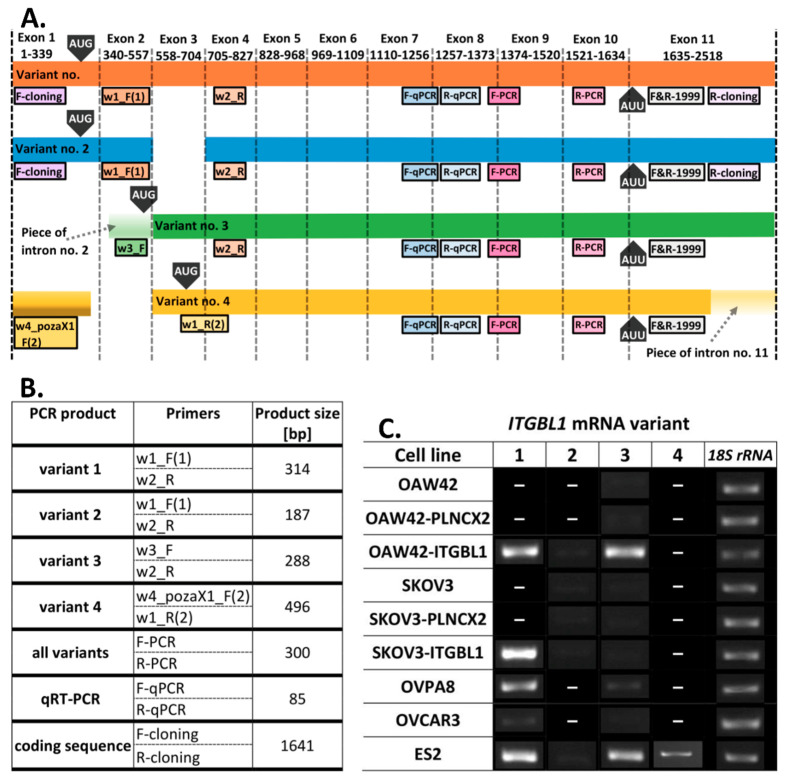
Detection of *ITGBL1* mRNA in different ovarian cancer cell lines. (**A**) Schematic representation of four predicted *ITGBL1* mRNA variants. Location of primers used for unambiguous detection is indicated by boxes below each mRNA variant. Primers used for concurrent detection of all four isoforms are marked in pink (F-PCR, R-PCR); primers used for cloning of the whole *ITGBL1* coding sequence (CDS) are marked in violet (F-cloning and R-cloning); as a reminder, we show location of primers used for quantitative RT-PCR (F-qPCR, R-qPCR; marked in blue). (**B**) Primer sets used for different purposes and PCR product sizes. (**C**) RT-PCR detection of different *ITGBL1* mRNA isoforms in wild-type and genetically modified cell lines. The ribosomal 18S rRNA served as the reference. Full gel electrophoresis images from (**C**) are shown in Supplementary material 2.

**Figure 2:**
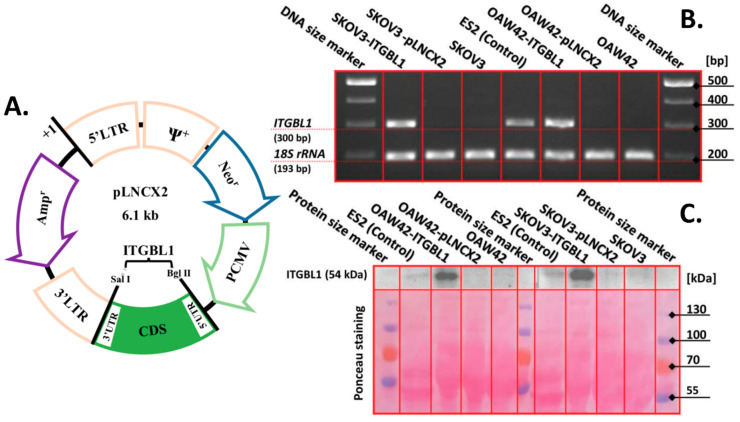
Construction and verification of cellular models. (**A**) RT-PCR amplified *ITGBL1* CDS (variant 1 mRNA) was cloned into expression vector pLNCX2. (**B**) RT-PCR revealed that *ITGBL1* mRNA (concurrent amplification of all isoforms) is detectable in the wild-type ES2 cell line (positive control) and in OAW42 and SKOV3 cell lines stably transduced with pLNCX1-ITGBL1 construct, while absent in wild-type and empty pLNCX2-transduced OAW42 and SKOV3 cells. (Supplementary Material 3A) No ITGBL1 signal could be detected by WB in the protein extracts from each analyzed cell line using Sigma-Aldrich HPA005676 antibody. (**C**) WB analysis with the same antibody confirmed presence of ITGBL1 in the culture media from indicated cell cultures (upper panel). Ponceau stained blot is shown to demonstrate protein loading uniformity (lower panel). Full blots from (**C**) are shown in Supplementary Material 3. Full gel electrophoresis image from (**B**) is shown in Supplementary material 2.

Except full size mRNA (variant 1 mRNA, NCBI Reference Sequence: NM_004791.2), three additional mRNA variants of *ITGBL1* (variants 2–4: NM_001271755.1, NM_001271756.1, NM_001271754.2, respectively) have been computationally predicted (Figure 1A). For this reason, we evaluated the presence of all four *ITGBL1* variants in different ovarian cancer cell lines (Figure 1B,C, and Figure 2B).

We analyzed four commercially available cell lines (OAW42, SKOV3, ES2, and OVCAR3) and one cell line (OVPA8) established by our group [6]. ES2 was the only cell line expressing all four mRNA variants. The OVPA8 cell line showed considerable amounts of variant 1 mRNA and trace amounts of variant 3, while the OVCAR3 cell line expressed only low amounts of both these variants. OAW42 and SKOV3 cell lines had the lowest expression level of any *ITGBL1* mRNA. The latter two were chosen to produce cell line variants with *ITGBL1* overexpression.

### 2.2. Production of ITGBL1-Overexpressing Cell Lines

Total RNA was extracted from GM07532 fibroblast cells. The whole *ITGBL1* coding sequence (variant 1 mRNA, NM_004791.2) was RT-PCR amplified and cloned into retroviral expression vector pLNCX2 (Figure 2A). Recombinant vector was transduced into the target cells, resulting in OAW42-ITGBL1 and SKOV3-ITGBL1 cell lines. Abundant *ITGBL1* mRNA expression could be detected in these cells by RT-PCR (Figure 2B) when using primer pair designed for concurrent amplification of all mRNA variants (Figure 1B). When using variant-specific primer pairs, OAW42-ITGBL1 cells were found to express significant amounts of variants 1 and 3, while trace amounts of variant 2; SKOV3-ITGBL1 expressed very high amounts of variant 1 and trace amounts of variants 2 and 3. Comparing to wild type ovarian cancer cell lines, it can be assumed that obtained expression patterns fit within naturally occurring frames (Figure 1C). Interestingly, anti-ITGBL1 antibody detected ITGBL1 protein in the culture media from OAW42-ITGBL1 and SKOV3-ITGBL1 culture (Figure 2C), while not in the cellular lysates (Supplementary Material 3A), indicating that in the cell culture conditions ITGBL1 is efficiently secreted. It must be noted that ITGBL1-overexpressing OAW42 and SKOV3 cells produce much higher amounts of the protein than wild type ES2 cells.

OAW42-PLNCX2 and SKOV3-PLNCX2 cell lines (containing an empty vector) were produced to serve as a control in further experiments. In these cells, *ITGBL1* mRNA expression pattern did not differ from wild type counterparts (Figure 1C and Figure 2B) and no ITGBL1 protein could be detected neither in cell lysates nor in culture media (Supplementary Material 3A and Figure 2C).

The ES2 cell line is of unclear histological origin: it is sold as a model for clear-cell ovarian cancer, but this identity has been questioned in some studies that have suggested high-grade serous ovarian cancer (HG-SOC) origin of these cells (reviewed in: [27]). The OVPA8 cell line is definitely of HG-SOC origin, and OVCAR3 is most probably HG-SOC, too. OAW42 cells probably represent serous ovarian cancer, but are unlikely HG-SOC, while the SKOV3 line is also uncertain, representing either serous or clear-cell origin [27]). Taken together, these data suggest that there is no correlation between the expression pattern of *ITGBL1* mRNA variants and the histological type of ovarian cancer.

### 2.3. ITGBL1 Overexpression Results in Altered Adhesiveness of Ovarian Cancer Cells

**Figure 3:**
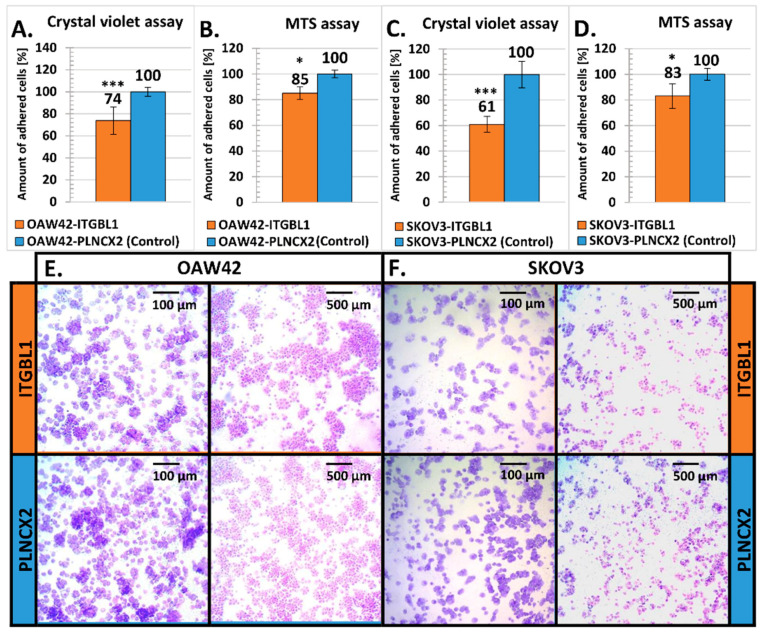
Evaluation of cellular attachment to the uncoated plastic surface. The amount of surface-attached cells was measured 5 min (OAW42) and 15 min (SKOV3) after seeding by crystal violet staining (**A**,**C**) and by MTS (**B**,**D**). Statistical significance was assessed with Student’s *t*-Test; * indicates *p* < 0.01, *** *p* < 0.001. *Y*-axis represents percentage of crystal violet/formazan released from fixed attached cells (mean ± SD, *n* = 3, each in 12 technical repeats). The amount obtained from control cells was taken as the reference value (100%). (**E**,**F**) Representative images of surface-attached cells, *ITGBL1*-overexpressing and control (crystal violet staining).

Since EGF-like domains may participate in regulating cellular adhesiveness, we have analyzed whether *ITGBL1* overexpression can affect this process. Cellular adhesion is a multistep process; the first phase is mediated mostly by physico-chemical interactions with the culture vessel surface; the second phase relies on integrin binding while full attachment involves formation of focal adhesions [28,29]. To analyze these, we used two experimental approaches: attachment assay and spreading assay [30]. The first one was performed quickly after seeding: 5 min in the case of the OAW42 line and 15 min in the case of the SKOV3 line. Both, crystal violet staining (Figure 3A,C,E,F) and MTS assay (Figure 3B,D), revealed that *ITGBL1* overexpression resulted in significantly reduced initial attachment of the cells to the uncoated plastic surface *(p* < 0.01 and *p* < 0.001, respectively).

**Figure 4:**
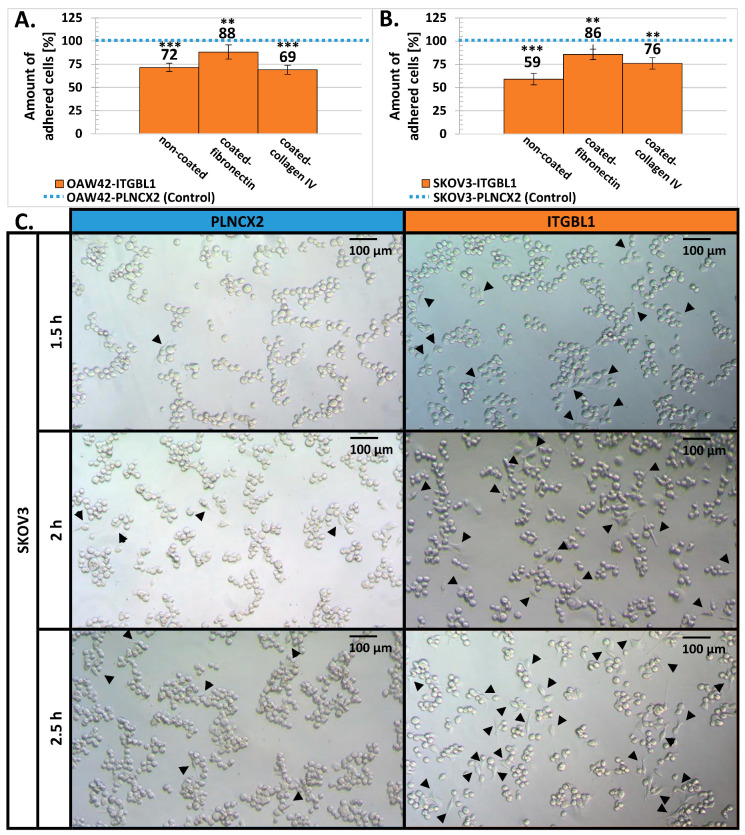
The effect of *ITGBL1* overexpression on the adhesiveness of OAW42 and SKOV3 cells. (**A**,**B**) comparison of cellular attachment to uncoated and fibronectin or collagen coated plastic surface (crystal violet assay), 5 min (OAW42) and 15 min (SKOV3) after seeding. *Y*-axis represents percentage of crystal violet released from fixed adherent cells (mean ± SD, *n* = 3, each in 12 technical repeats). The amount obtained from control cells was taken as the reference value (100%). Statistical significance was determined by one-way analysis of variance (ANOVA) with Scheffe’s adjustment for pairwise comparisons; ** indicates *p* < 0.01, *** *p* < 0.001. (**C**) Spreading assay. Images taken at the indicated time points after seeding show that *ITGBL1*-overexpressing cells tend to adopt a spread morphology (flatten) quicker than control cells. Flattened cells are indicated by arrowheads.

We also analyzed initial cellular attachment to the surface coated with fibronectin or collagen IV (Figure 4A,B). Fibronectin coating partially abolished the effect of *ITGBL1* overexpression, but adhesiveness of OAW42-ITGBL1 and SKOV3-ITGBL1 cells was still reduced in comparison to the control ones. Covering of the culture vessel surface with collagen IV had an ambiguous and cell line-dependent effect: in the case of OAW42, *ITGBL1*-overexpressing cells had even weaker attachment than to the uncoated surface, while in the case of SKOV3, *ITGBL1*-overexpressing cells attached stronger than to a bare surface, but weaker than to the fibronectin-coated one.

Next, we used spreading assay [30] which allowed to evaluate the number of cells that have adopted spread morphology at 1.5–2.5 h after seeding. We found that *ITGBL1-*overexpressing cells tended to adopt spread morphology (flatten) quicker than control cells (Figure 4C). Thus, *ITGBL1* overexpression resulted in weakened initial attachment of the cells but accelerated cellular spreading in the next steps of cellular adhesion.

### 2.4. ITGBL1 Overexpression Promotes Ovarian Cancer Cell Migration and Invasion Rate

**Figure 5:**
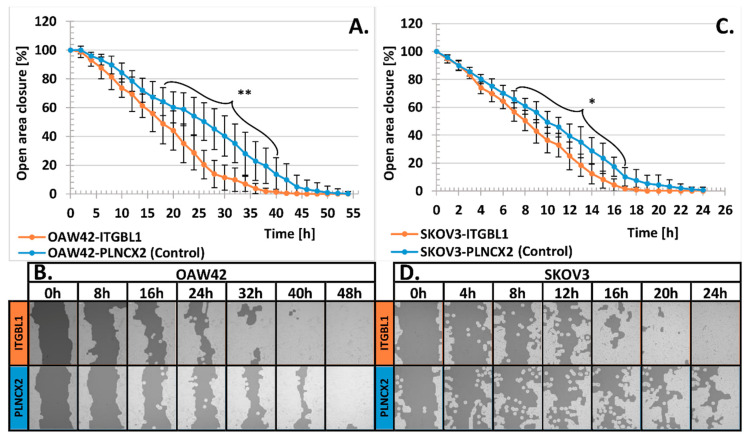
Scratch assay. (**A**) Comparison of scratch area filling time required for OAW42-ITGBL1 and OAW42-PLNCX2 cells. (**C**) Comparison of scratch area filling time by SKOV3-ITGBL1 and SKOV3-PLNCX2 cells; *X*-axis represents observation time points, *Y*-axis represents size of the remaining scratch area (mean ± SD, *n* = 3, each in 10 technical repeats). The initial size of scratch area was assumed as 100%. Statistical significance was assessed with Student’s *t*-Test with Bonferroni correction for multiple testing; * indicates *p* < 0.05; ** *p* < 0.01. (**B**,**D**) Representative images showing scratch filing by *ITGBL1*-overexpressing and control cells at the indicated time points.

As we found that *ITGBL1* overexpression altered ovarian cancer cells adhesiveness, we further analyzed how ITGBL1 affects cognate cellular functions, like motility and invasiveness. When analyzing cellular migration rate by scratch assay, we observed that OAW42-ITGBL1 and SKOV3-ITGBL1 cells had significantly higher motility than isogenic control cells (*p* < 0.01 and *p* < 0.05, respectively; Figure 5).

**Figure 6:**
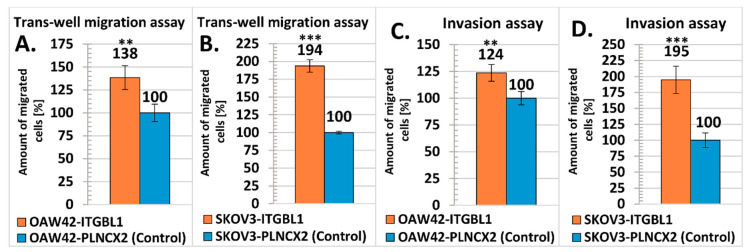
Comparison of trans-well migration rate and Matrigel invasiveness of *ITGBL1* overexpressing and control cell lines. (**A**,**B**) Trans-well migration assay. *Y*-axis represents percentage of cells that migrated through the membrane with 8 μm pores. (**C**,**D**) Matrigel invasion assay. *Y*-axis represents the percentage of cells that migrated through Matrigel coated trans-well inserts with 8 μm pores (mean ± SD, *n* = 3, each in 9 technical repeats). The result obtained with control cells was taken as the reference value (100%). Statistical significance was assessed with Student’s *t*-Test; ** indicates *p* < 0.01; *** *p* < 0.001.

We also analyzed cellular migration rate using trans-well migration assay. Both OAW42 and SKOV3 cells with *ITGBL1* overexpression showed significantly higher migration rate than isogenic control cells (*p* < 0.01 and *p* < 0.001, respectively; Figure 6A,B).

In addition, we found that both OAW42 and SKOV3 cells with *ITGBL1* overexpression had significantly higher ability to invade through Matrigel than their isogenic controls (*p* < 0.01 and *p* < 0.001, respectively; Figure 6C,D).

### 2.5. ITGBL1 Has No Effect on the Proliferation Rate of Ovarian Cancer Cells

Proteins with EGF-like repeats may potentially exhibit growth factor activity. Thus, we analyzed proliferation rate of OAW42-ITGBL1 and SKOV3-ITGBL1 cells, in comparison to their respective controls—OAW42-PLNCX2 and SKOV3-PLNCX2. We did not observe differences in proliferation rate between control and ITGBL1-overexpressing cells neither by using crystal violet (Supplementary Material 4.1. A,C) or by using MTS assay (Supplementary Material 4.1. B,D).

Using the same cell lines we have also evaluated distribution of the cell cycle phases by flow cytometry. We observed no difference between *ITGBL1*-overexpressing and control cells (Supplementary Material 4.2. A,B).

### 2.6. ITGBL1 Overexpression Results in Increased Chemoresistance of Ovarian Cancer Cells

**Figure 7:**
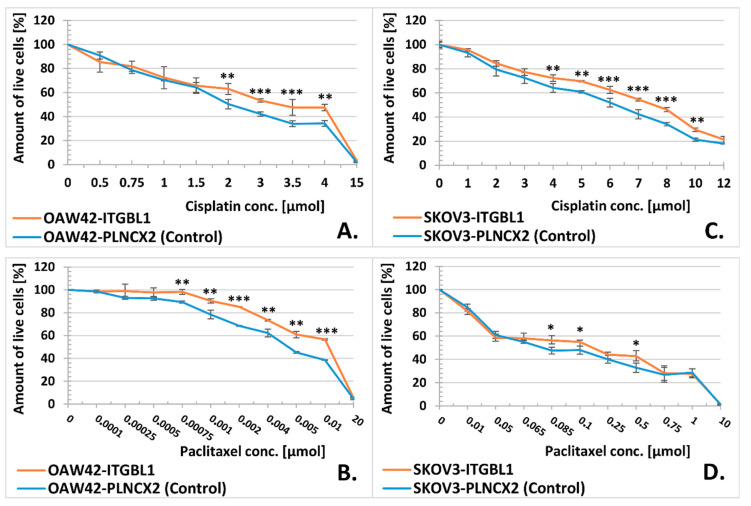
MTS assay for evaluation of cellular sensitivity to cisplatin (**A**,**C**) and paclitaxel (**B**,**D**). *Y*-axis represents percentage of live cells (mean ± SD, *n* = 3, each in 6 technical repeats) after 72 h of incubation with a drug, at an indicated concentration (*X*-axis). The absorbance of formazan from control (untreated) cells was taken as the reference value (100%). Statistical significance was assessed with Student’s *t*-Test; * indicates *p* < 0.05, ** *p* < 0.01, *** *p* < 0.001.

We also checked whether *ITGBL1* overexpression may alter cellular sensitivity toward drugs used in the standard first line chemotherapy for ovarian cancer, i.e., cisplatin and paclitaxel [31]. Indeed, cell lines overexpressing *ITGBL1* were slightly but significantly more resistant to cisplatin and to paclitaxel, as compared to control ones (Table 1, Figure 7).

### 2.7. Gene Expression Profiling and Signaling Pathways Related to ITGBL1

**Figure 8:**
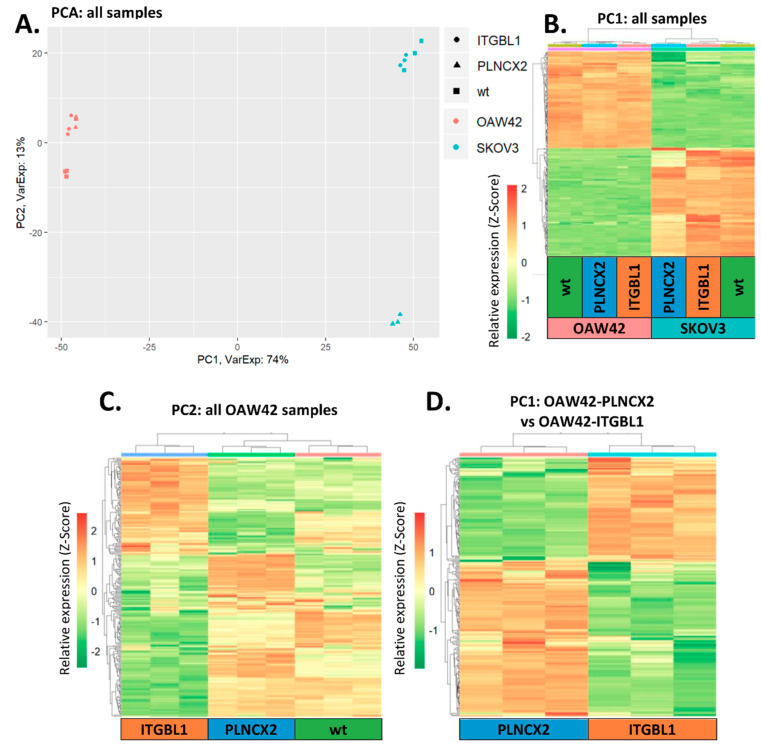
Unsupervised analysis of gene expression profiles in the analyzed cell lines. (**A**) Principal Component Analysis performed on all samples. Samples tend to group by the cell line. This difference is most prominent (74% of variance) and is described by first principal component (PC1). (**B**) Hierarchical clustering of all samples according to PC1. Heat map demonstrates that genes from PC1 have distinct expression pattern in OAW42 and SKOV3 cells. (**C**) Hierarchical clustering of OAW42 samples according to PC2. PC2 differentiates *ITGBL1*-overexpressing OAW42 samples from all control OAW42 samples (wild-type and with an empty pLNCX2). (**D**) Hierarchical clustering of OAW42 samples (only empty pLNCX2 controls and samples with *ITGBL1* overexpression).

To analyze the influence of ITGBL1 on cellular networks and signaling pathways we evaluated, using DNA microarrays, the global gene expression pattern of cells with and without *ITGBL1* overexpression. Using Principal Component Analysis (PCA), an unsupervised method of data analysis, we selected gene sets (principal components, PC) related to major sources of variability in our dataset. Then, we executed hierarchical clustering of samples based on expression of genes from each principal component; the results were visualized as a heat map plot for easier recognition of molecular differences between samples (key results are shown on Figure 8; the whole workflow and results of all analyses are contained in Supplementary Material 5).

First, we performed PCA based on all samples: SKOV3 and OAW42 cells, each cell line in a wild-type variant, with an empty pLNCX2 vector and with *ITGBL1* overexpression (Figure 8A; Supplementary Material 5A). We expected that this pan-analysis could reveal general hallmarks resulting from *ITGBL1* overexpression in ovarian cancer cells. However, we observed that the main diversity was related to the difference between two cell lines (OAW42 *versus* SKOV3), while not with ITGBL1. This is clearly visible in the distribution of samples according to the first principal component (PC1): samples are grouped by the cell line, not by the ITGBL1 status. The only exceptions are SKOV3-PLNCX2 samples which are placed slightly apart from all others. The latter difference is defined by second principal component (PC2). Of note, this difference is not very prominent, as PC2 accounts only for 13% of variance, while PC1 accounts for 74%. Hierarchical clustering based on expression of the genes from PC1 also clearly illustrates the difference between SKOV3 and OAW42 (Figure 8B). Expression pattern of genes from PC2 also distinguishes between cell lines, and additionally, portrays some unique features of SKOV3-PLNCX2 cell line (Supplementary Material 5B).

In the next step of analysis, we excluded the major source of variability which was identified as the difference between OAW42 and SKOV3 cell lines. This difference represented a main confounding factor in the search for ITGBL1-related changes. Thus, we performed PCA on each cell line separately (Supplementary Material 5C and 3F). In both cell lines, hierarchical clustering using PC1 genes did not allow us to distinguish *ITGBL1*-overexpressing samples from control ones (Supplementary Material 5D and 3G). On this basis we assumed that this gene set was not related to *ITGBL1*.

Finally, hierarchical clustering based on PC2 showed that most of the genes consistently had similar expression patterns in both types of control samples (wild type and empty vector-containing), while they were distinct in *ITGBL1*-overexpressing samples (Figure 8C, Supplementary Material 5D and 3G). We assumed that these genes were related to *ITGBL1* status. Consistently, genes from PC2 were used for signaling pathways analysis (full lists of signaling pathways from all consecutive comparisons can be found in Supplementary Material 5).

In OAW42 cells we found 72 significantly affected pathways, out of which 21 were related to ECM, focal adhesion, integrin signaling, etc. In SKOV3 cells there were 44 significant pathways, out of which 18 were associated with cellular communication, ECM, integrin signaling, etc.

At the end, we performed PCA and signaling pathways analysis using only one type of control sample (empty pLNCX2-containing; Figure 8D). This comparison was consistent with the model system used in all above described functional analyses. In OAW42 we found 76 significantly affected pathways, out of which 22 were related to ECM, integrin signaling, focal adhesion, cellular motility, etc. (Table 2).

In SKOV3 there were 146 significant pathways, among them 44 related with ECM, cell junction, cellular motility, ERBB2 and ERBB4 signaling, etc. (Table 3). The results of signaling pathways analysis are concordant with main functional changes observed in *ITGBL1*-overexpressing cells, like altered adhesiveness, enhanced motility and invasiveness.

## 3. Discussion

### 3.1. Cellular Adhesion, Migration, and Invasion

Cellular adhesion is a complex, dynamic process that can be divided into three phases [28,29,30,32]. Phase I is driven mostly by electrostatic forces; cells are attached, but remain round; it is typically studied by so called attachment assay. Phase II is mediated by integrin bonding; at this stage cells start flattening. Third phase is characterized by establishment of focal adhesions and full spreading of cells. To evaluate latter two phases spreading assay is recommended.

The influence of ITGBL1 on ovarian cancer cells adhesion was studied by Sun et al. [24] and by us. Despite differences in the experimental model, it can be assumed that both works evaluated phase II/III of cellular adhesion and both had concordant results.

By the means of spreading assay we found that *ITGBL1*-overexpressing cells adopt spread morphology faster than control cells within the time frame of 1.5–2.5 h after seeding. Sun et al. [24], who performed attachment assay at one hour after seeding, observed stronger attachment of ITGBL1-containing cells. Taken together, these results indicate that between 1 and 2.5 h after seeding ovarian cancer cells with higher ITGBL1 level have the advantage of faster spreading and creating stronger bonds with the plastic surface, either bare or coated with ECM proteins. In addition, we had an intriguing, but unique observation that during phase I of cell adhesion (5–15 min after seeding, time-points not analyzed by Sun et al. [24]), *ITGBL1* overexpression resulted in diminished cellular attachment.

Opposite results to ours and those of Sun et al. [24], were obtained in a very elegant work by Song E.K. et al. [18], who studied the role of ITGBL1 in chondrogenesis. First, they showed by co-immunoprecipitation that in the presence of Ca^2+^, ITGBL1 can directly interact with integrin β and inhibit its activity: depletion of ITGBL1 increased, whereas *ITGBL1* overexpression reduced the amount of active integrin β in PC3 prostate cancer cells. Next, they analyzed cell spreading and focal adhesion at 8 h after seeding. Strikingly, ITGBL1 depletion increased cell spreading, whereas *ITGBL1* overexpression caused PC3 cells to detach from collagen-coated plates. Similar results were obtained with human chondrocytes and bone marrow stem cells (hBMSC) [18]. This is an opposite effect than observed in ovarian cancer cells, which is confusing since in both cancers (prostate and ovarian) ITGBL1 is proposed as a negative prognostic factor. To try to mitigate this contradiction one could take into account that these cancers have completely different mechanisms of metastasis and progression: ovarian cancer spreads mostly by intraperitoneal seeding, while prostate cancer produces distant metastases via the lymphatic system; thus, in each case, different cellular behaviors may be responsible for the acceleration of cancer progression.

Regulation of adhesion is an indispensable component of cellular migration and invasion. Six studies, including ours, have analyzed ITGBL1 influence on cellular migration and invasiveness [14,23,24,33,34]. We have shown, similar to Sun et al. [24], that in ovarian cancer ITGBL1 enhances both of these processes. The same observation concerns colorectal [34], prostate [23], and hepatocellular cancer [33] cells. The only exception is NSCLC [14], where the addition of rITGBL1 was shown to inhibit both migration and invasion.

Of note, we observed that wild-type ovarian cancer cell line ES2 is characterized by exceptionally high migration rate. Interestingly, this cell line is the only one, from those we tested, expressing all four *ITGBL1* mRNA variants and has the highest ITGBL1 level. These observations further support our hypothesis that ITGBL1 may positively impact on ovarian cancer cells migration and invasiveness.

### 3.2. Signaling Pathways and ITGBL1 Co-Expressed Genes

Several approaches, both experimental and in silico, have been used to elucidate involvement of ITGBL1 in cellular signaling pathways (summarized in Supplementary Material 1).

Based on experimental data, ITGBL1 has been proposed to be implicated in the following signaling pathways:

- Wnt/PCP (activated by ITGBL1 in ovarian cancer [24], but suppressed in lung cancer [14]; concordant with the proposed oncogenic role of ITGBL1 in the first one, and tumor suppressor role in the latter).
- FAK/SRC (activated in ovarian cancer [24]).
- TGFβ (upregulated by ITGBL1 in hepatocellular cacinoma [28], and in breast cancer [10]).
- TNFAIP3/NF-κB (stimulated by ITGBL1 in prostate cancer [23] and in fibroblasts activated by ITGBL1 released from colorectal cancer primary tumor in extracellular vesicles [20]),
- PI3K/Akt (activated by ITGBL1 in ovarian cancer [18]).

In-silico analyses indicated following pathways to be affected by ITGBL1:

- TGBβ1 (in liver fibrosis [16]).
- KRAS/EMT (in gastric cancer [22]).
- Wnt/β-catenin (in colorectal cancer [19]).

In our study, we found that signaling pathways significantly associated with *ITGBL1* overexpression were mostly related with ECM organization and function, integrin signaling, focal adhesion, cellular communication and motility. This is concordant with the results of our functional studies which show that ITGBL1 affects cellular migration, adhesion and invasiveness. Analogous gene expression experiment was done by Huang et al. [33], who performed RNA-seq in *ITGBL1*-overexpressing *versus* control SMMC-7721 hepatocellular carcinoma cells. They selected 196 differentially expressed genes; this list only partially overlaps with our lists of differentially expressed genes identified in SKOV3 and OAW42 models (96 and 48 common genes, respectively). From 12 genes selected by Huang et al. [33] for further, more detailed studies, several were present also in our comparisons; in particular, *CDH2* and *VIM* (found in both OAW42 and SKOV3), as well as *FOS*, *VEGFA* and *FOXO1* (found exclusively in SKOV3). The comparison of our lists of differentially expressed genes and the list obtained by Huang et al. [33] is shown in Supplementary Material 6.

We also observed slightly increased in-vitro chemoresistance in ITGBL1-overexpressing cells; this may be explained only by more efficient ECM proteins deposition providing physical barrier for drug diffusion [35,36], since no classical pathways related with chemoresistance were found in our analysis. Of note, Song et al. [25] also showed that ITGBL1 confers in-vitro resistance to cisplatin and paclitaxel, as well as in-vivo resistance to cisplatin (paclitaxel not tested). From among cell lines tested, only SKOV3 were overlapping with our experiment; interestingly, Song et al. [25] observed much higher IC50 values for cisplatin than we did, and much greater difference in IC50 between control and ITGBL1-overexpressing SKOV3 cells.

In breast [13] and colorectal cancer [20] *Runx2* (*Runt-related transcription factor 2*) was found among ITGBL1 co-expressed genes, and functional studies demonstrated its role in transcriptional regulation of *ITGBL1*. In our microarray data, we observed several RUNX2 related pathways when using Reactome online software, e.g., “RUNX2 regulates genes involved in cell migration”, “RUNX3 Regulates Immune Response and Cell Migration”, “Transcriptional regulation by RUNX2”. *RUNX2* was also found among differentially regulated genes in ITGBL1 overexpressing *versus* control SKOV3 cells (but not in OAW42). It suggests that ITGBL1 could remain under transcriptional control of *RUNX2* also in ovarian cancer.

### 3.3. Technical Constraints

A key method employed in a majority of the above-discussed experiments is Western blotting (WB). However, ITGBL1 has been more widely studied for no more than five years, and there is no anti-ITGBL1 antibody on the market that has been reliably validated. There is also a lot of additional uncertainty, because some authors do not mention catalogue number/company name of the antibody producer. We have summarized our investigations concerning anti-ITGBL1 antibodies used in twelve studies in Supplementary Material 7.

The greatest discrepancy between our observations and those from [13,14,15,16,22,23,24,25,33,34] is that in all these works authors were able to detect ITGBL1 (either endogenous or expressed from the vector) in the whole-cell lysates, while we were not. We could detect ITGBL1 only in the concentrated culture media. This difference could be caused by the fact that we used a different antibody (HPA005676, Sigma-Aldrich, Saint Louis, MO, USA). We chose this product some time ago, based on the analysis of multiple examples of protein staining with this antibody displayed in the Human Protein Atlas (HPA). In the case of ovaries, HPA showed very weak ITGBL1 staining in follicle cells, no staining in stromal cells, variable staining in ovarian cancer samples (from weak to absent) and weak in EFO21 ovarian cancer cell line. However, these images were later withdrawn from the HPA website, possibly due to their uncertainty.

It seems that Song E.K. et al. [18] used the same antibody from Sigma-Aldrich (they did not mention cat. no), but only for IHC (they obtained positive staining in cartilage chondrocytes). However, they do not show any example of ITGBL1 detection by WB; instead, they used RT-PCR or tag detection in the case of recombinant ITGBL1-HA protein. This may suggest that they also encountered difficulties with WB detection of ITGBL1 in cellular lysates.

We also tested three other antibodies which have been sold with recommendation for WB (Supplementary Material 3). These were (1) ProSci (cat. no 29-712; Poway, CA, USA), (2) ABGENT (cat. no Ap8781c; San Diego, CA, USA), (3) Thermo Fisher Scientific (cat. no PA5-42123; Waltham, MA, USA). With none of these antibodies we were able to obtain a band corresponding to ITGBL1.

### 3.4. ITGBL1 Function

It is still unclear, how mechanistically ITGBL1 performs its functions. In our opinion, ITGBL1 should not be regarded as a functional counterpart of integrins, as it has considerably unlike the domain structure. ITGBL1 contains one functional domain built of ten EGF-like repeats and signal peptide that warrants protein secretion. EGF-like repeats may exert growth factor activity and/or may mediate specific protein–protein interactions. We analyzed the influence of ITGBL1 on ovarian cancer cell proliferation rates and cell cycles, but we did not notice any changes. On the contrary, in colorectal cancer, depletion of ITGBL1 was shown to result in decreased cellular proliferation in vitro. This may suggest that ITGBL1 function is cancer type-specific. The latter is supported also by the studies on the ITGBL1 role in lung cancer and in leukemia, in which two cancers with this protein exert not oncogenic, but tumor suppressive function.

We suppose that plethora of ITGBL1-related cellular effects can result from its interactions with diverse proteins and modulation of their functions. In one study ([18]), ITGBL1 was shown to directly interact with integrins (in Ca^2+^ dependent manner) and to inhibit their functions in prostate cancer PC3 cells, hBMSC and chondrocytes. This suggests that the influence of ITGBL1 on adhesion, migration and invasiveness may be mediated by interactions with integrins. However, Ji et al. [20] found no direct ITGBL1-integrin interactions in colorectal cancer cells (Supplementary Material 1). This may again point to the tissue-specific activity of ITGBL1 or may simply result from technical differences (e.g., lack of Ca^2+^ ions in the latter experiment).

Based on in-silico analysis of gene expression data from colorectal cancer Qi et al. proposed ITGBL1-related protein–protein interaction network [19]. It consists of a dozen or so of ITGBL1 binding proteins, among them three (FN1, CTNNB1, COL1A1) which could provide a link with extracellular Wnt signaling pathway. Of note, four genes from this network were overlapping with our previously published negative prognostic signature (FN1, COL1A1, SFRP2 and SFRP4) [2]. We have also previously shown that FN1 (evaluated by IHC) is an independent prognostic factor for ovarian cancer patients [30].

Ji et al. [20] isolated several ITGBL1-binding partners from fibroblasts, among them TNFAIP3 (Tumor necrosis factor alpha-induced protein 3), on which they focused their further studies. Other binding partners include several proteins involved in primary metabolism: PGK1 (Phosphoglycerate kinase 1), GAPDH (Glyceraldehyde-3-phosphate dehydrogenase), LDHA and LDHB (L-lactate dehydrogenase A chain, and B chain, respectively), and particularly in glucose metabolism: ALDOA (Fructose-bisphosphate aldolase A), TPI1 (Triosephosphate isomerase), ENO1 (Alpha-enolase; also involved in growth control, hypoxia tolerance and allergic responses). Two stress proteins strongly binding to ITGBL1: HSPA5 (Glucose-Regulated Protein 78) and HSPA8 (Constitutive Heat Shock Protein 70), are also engaged in basic metabolism (protein synthesis and folding). Other ITGBL1-interacting proteins were related to nucleotide metabolism, DNA methylation, and repair: NME1 (Nucleoside diphosphate kinase A, also involved in cell proliferation, differentiation, signal transduction, etc.), AHCY (Adenosylhomocysteinase), PCNA (Proliferating Cell Nuclear Antigen). Interactions with these proteins could delineate ITGBL1 functions and are worth further study.

## 4. Materials and Methods

### 4.1. Cell Culture and Experimental Conditions

The human ovarian cancer cell lines: SKOV3 (ATCC-HTB-77), ES2 (ATCC-CRL-1978), OVCAR3 (ATCC-HTB-161) and OAW42 (ECACC-85073102) were obtained from American Type Culture Collection (ATCC, Manassas, VA, USA) and from European Collection of Authenticated Cell Cultures (ECACC, Salisbury, UK). OVPA8 (ECACC-19061601) is a human ovarian cancer cell line established by our group [37]. PT67, a retrovirus packaging cell line derived from TK- NIH/3T3 cells was purchased from ATCC. GM07532, a human fibroblast cell line from a healthy female donor was obtained from Coriell Institute for Medical Research (Camden, NJ, USA). SKOV3 cells were maintained in McCoy’s medium (Sigma-Aldrich, Saint Louis, MO, USA), PT67 cells were grown in DMEM (Sigma-Aldrich), other cell lines were cultured in RPMI (Sigma-Aldrich, Saint Louis, MO, USA): for ES2, OVCAR3, GM07532, OAW42 RPMI medium was supplemented with 0.1% insulin (Sigma-Aldrich), for OVPA8-with 2 mM L-glutamine (Sigma-Aldrich). Until otherwise stated, cell cultures were performed with addition of 10% fetal bovine serum (Gibco, Carlsbad, CA, USA), 100 µg/mL streptomycin and 100 U/mL penicillin (Sigma-Aldrich), at 37 °C, in a humidified environment of 5% CO_2_ atmosphere. All cultures were regularly checked for mycoplasma contamination.

### 4.2. Total RNA Extraction and Reverse Transcription

Total RNA was extracted from the cells using RNeasy Mini Kit (Qiagen, Hilden, Germany) with simultaneous on column DNase I digestion, according to the manufacturer’s instructions. RNA purity and concentration were estimated with Nanodrop ND-2000 spectrophotometer (Thermo Fisher Scientific, Waltham, MA, USA). For oligonucleotide microarray experiment, RNA quality was assessed using the 2100 Bioanalyzer with the RNA 6000 Nano Kit (Agilent Technologies, Santa Clara, CA, USA) and only samples with RNA integrity number (RIN) > 7.0 were used. For reverse transcription-PCR, RNA quality was evaluated by agarose gel electrophoresis, then half a µg of total RNA was used for cDNA synthesis with the Omniscript RT Kit (Qiagen, Hilden, Germany), according to the manufacturer’s instructions.

### 4.3. ITGBL1 CDS Cloning and Sequencing

Retroviral vector encoding the ITGBL1 protein under the control of CMV promoter was constructed by insertion of the ITGBL1 coding sequence between BglII and SalI restriction sites of the pLNCX2 plasmid (Clontech, Takara Bio, Mountain View, CA, USA). The ITGBL1 coding sequence (CDS; GenBank accession number NM_004791.2), was amplified by PCR with GM07532 cDNA as a template. Primers used for cloning are shown in Table 4. The 1641 bp PCR product was obtained using Phusion High-fidelity DNA polymerase (Thermo Fisher Scientific, Waltham, MA, USA), annealing temperature 65 °C. The PCR amplified product was recovered from the electrophoretic agarose gel using GeneJET Gel extraction kit (Thermo Fisher Scientific, Waltham, MA, USA), according to the manufacturer’s instruction. The purified DNA product and pLNCX2 vector were digested with restriction enzymes, BglII and SalI. The digested products were gel purified and ligated using T4 DNA ligase (Rapid DNA Ligation Kit, Thermo Fisher Scientific, Waltham, MA, USA), according to the manufacturer’s instruction. The E. coli Mach1 competent cells were transformed with the 5 µL ligation mixture by heat shock method. The recombinant clones were screened using restriction enzymes digestion. The clone with appropriate structure was propagated in Luria Bertani (LB) medium (Difco, Franklin Lakes, NJ, USA) supplemented with ampicillin (100 µg/mL). High quality plasmid DNA was isolated using GeneJET Plasmid Midiprep Kit (Thermo Fisher Scientific, Waltham, MA, USA), according to the manufacturer’s instruction. The nucleotide sequence of pLNCX2-ITGBL1 plasmid was verified by DNA sequencing (service provided by Genomed S.A., Warsaw, Poland).

### 4.4. Generation of Stably ITGBL1-Overexpressing Cell Lines

Retroviruses were produced by transfecting plasmids (pLNCX2-ITGBL1 or empty pLNCX2) into PT67 packaging cells, according to the manufacturer’s instructions (Retroviral Gene Transfer Technology, Clontech, Takara Bio, Mountain View, CA, USA). OAW42 and SKOV3 cells (2 × 105/6-cm φ dishes) were transduced at 24 h after plating with supernatants containing retroviruses for 24 h at 37 °C with the addition of 8 µg/mL polybrene (Sigma-Aldrich, Saint Louis, MO, USA). Then, culture medium was replaced with the fresh one. This procedure was repeated three times. Stable cell lines which survived geneticin selection (G418, Sigma-Aldrich; OAW42–50 µg/mL, SKOV3–100 µg/mL) were used in further experiments.

### 4.5. Protein Extraction and Western Blot Analysis

Cells were seeded onto 6cm-diameter dishes and propagated until 70–80% confluence. To prepare total protein extracts, cells were lysed by scrapping in IP buffer (50 mM Tris–HCl pH 7.5, 150 mM NaCl, 0.1% Nonidet P-40, 50 mM NaF, 1 mM DTT, 1 mM PMSF) supplemented with Phosphatase Inhibitor cocktail and protease inhibitor mixture (cOmplete^TM^; Roche, Basel, Switzerland).

To prepare protein extracts from culture medium, centrifugal concentrators were used (Vivaspin MWCO 10 kDa; Sartorius, Gottingen, Germany). Cells were seeded as above and cultured until 70–80% confluence. Then, culture medium was changed to a serum-free one and collected after 24 h, then centrifuged using concentrators according to the manufacturer’s instructions.

Total protein content was determined using Protein Assay Kit (Bio-Rad, Hercules, CA, USA). 25–50 μg of total proteins were fractionated by SDS-PAGE on 8% polyacrylamide gels and transferred onto nitrocellulose membrane using the Trans Blot Turbo system (Bio-Rad), for 10 min. Membrane was blocked (60 min) in 5% nonfat milk/TTBS (0.25 M Tris–HCl pH 7.5, 0.15 M NaCl, and 0.1% Tween-20), and incubated overnight at 4 °C with primary antibody (Table 5). Antibody–antigen interaction was detected using secondary antibody and visualized using SuperSignal^®^ West Pico Chemiluminescent Substrate Kits (Thermo Fisher Scientific, Waltham, MA, USA). Anti-β-actin antibody was used as a loading control. In the case of protein detection in culture medium Ponceau staining served as a loading control. The primary antibodies were anti-ITGBL1 (HPA005676, Sigma-Aldrich, Saint Louis, MO, USA; dilution 1:750) and anti-β-actin (MAB1501, Milipore, Burlington, MA, USA; dilution 1:1000).

### 4.6. The primer Design and Semi-Quantitative PCR

Primers were designed using online Primer3 software v. 0.4.0 (Whitehead Institute for Biomedical Research, Cambridge, MA, USA). Primer sequences are shown in Table 4. The PCR was performed in the volume of 20 µL; mixture contained 1 × Phusion HF buffer, dNTPs (0.2 mM each), 0.4 U Phusion Hot Start II High-Fidelity DNA Polymerase (Thermo Fisher Scientific, Waltham, MA, USA), 0.3 pmol of each primer, and 25 ng cDNA. The PCR conditions included an initial denaturation at 98 °C for 30 s, followed by 35 cycles consisting of 30 s denaturation at 95 °C, 30 s primer annealing at a temperature indicated in Table 4, 30 s elongation at 72 °C in DNA Engine^®^ Thermal Cycler (Bio-Rad, Hercules, CA, USA). PCR products were separated by electrophoresis on a 2.5% agarose gel containing ethidium bromide. 18S rRNA was amplified as an internal control.

### 4.7. Scratch Assay

SKOV3 (4 × 10^4^ cells/well) and OAW42 (3 × 10^4^ cells/well) cells were seeded into 96-well plates, at ten replicates per sample. At about 90% confluency, a sterile 10 μL pipette tip was used to make a scratch across each well. The detached cells were removed by washing twice with a culture medium. The scratch closure was monitored during 48 h for SKOV3 and 72 h for OAW42, using a live-cell microscopy Cell Observer spinning disc confocal microscope with 10× objective magnification (100× total magnification) (Carl Zeiss, Oberkochen, Germany). Images were captured every 10 min. Image analysis was carried out with the TScratch software (CSE-lab, Zurich, Switzerland) using the default parameter settings. The experiment was repeated three times.

### 4.8. Transwell Migration Assay

Cells were resuspended in 0.1 mL of serum-free medium and seeded in 24-well Transwell inserts (8 μm pore size, BD Bioscience, San Jose, CA, USA), at a density of 7.5 × 10^4^ cells/per insert, at nine replicates per sample. Then, a 10% FBS-containing medium was added to the lower chamber to serve as chemoattractant. Cells were allowed to migrate through membrane pores for 4 h. Using a cotton swab, the non-migratory cells remaining in the upper chamber were removed and the inserts were rinsed with PBS. The cells that migrated across the membrane were fixed with cold methanol and stained with 0.1% crystal violet solution for 30 min, then washed 3 times with distillated water and air-dried. Afterwards, each membrane was soaked in 110 μL of 10% acetic acid for 10 min, to dissolve cell-associated crystal violet. Hundred μL of this solution were transferred into the 96-well plate, and the absorbance was measured at a wavelength of λ = 595 nm using a microplate reader Synergy 2 (BioTek, Winooski, VT, USA). The experiment was repeated three times.

### 4.9. Matrigel Transwell Invasion Assay

Cells were seeded in 24-well Transwell inserts (8 μm pore size, BD Bioscience, San Jose, CA, USA), coated with 200 µg/mL Matrigel (cat. no 354234; Corning, New York, NY, USA), at a density of 7.5 × 10^4^/per insert (suspended in 0.1 mL of serum-free medium), each sample in nine replicates. Medium containing 10% FBS was placed in the lower chambers to serve as chemoattractant. After 48–72 h of incubation, cells on the upper membrane surface were wiped off, and ones that invaded across the Matrigel-coated membrane were fixed with ice-cold methanol and stained with 0.1% crystal violet solution for 30 min, then washed 3 times with distillated water and air-dried. Invaded cells were observed under the inverted microscope and imaged (objective 20×, eyepiece magnification 10×; AxioVert 40 CFL with digital camera AxioCam Rec 5s, Carl Zeiss, Oberkochen, Germany). Afterwards, crystal violet was dissolved and absorbance measured as described above. The experiment was repeated three times.

### 4.10. In Vitro Cytotoxicity Assay

The following stock solutions were used: cisplatin (CDDP, 1 mg/mL, for infusion; Teva, Petah Tikva, Izrael) and paclitaxel (PTX, 100 mg/16.7 mL, for infusion; Teva, Petah Tikva, Izrael). The dose range of cisplatin and paclitaxel 0.5–35 µmol and 0.0001–30 µmol were used, respectively. Working solutions were prepared fresh before each experiment by dillution of stock in the culture medium without antibiotics. Control cells were incubated in culture medium without drug. SKOV3 (9 × 10^3^/well) and OAW42 (3 × 10^3^/well) cells were seeded onto 96-well plate (each sample in six replicates) and incubated with drugs for 72 h. Cell viability was estimated using MTS assay (CellTiter 96TM AQueous One Solution Assay, Promega, Madison, WI, USA) according to a manufacturer’s instructions. The absorbance of formazan product was measured (λ = 490 nm) using a microplate reader Synergy 2 (BioTek, Winooski, VT, USA). The experiment was repeated three times.

### 4.11. Cell Proliferation Assay

SKOV3 (2 × 10^3^ cells /well) and OAW42 (3 × 10^3^ cells/well) were seeded into 96-well plates, each sample in 18 replicates). At the indicated times (8, 24, 48, 72 and 96 h after plating) the metabolic activity of cells was measured with MTS assay, as described above. The metabolic activity values were calculated relative to the readouts obtained 8 h after plating. For crystal violet proliferation assay cells, at the indicated time points were washed with PBS, fixed in ice-cold methanol, stained with 0.1% crystal violet for 30 min, rinsed extensively with distilled water, and dried. Cell-associated dye was extracted with 10% acetic acid, aliquoted (200 μL) and the absorbance was measured (λ = 595 nm) using a microplate reader Synergy 2 (BioTek, Winooski, VT, USA). The experiment was repeated two times.

### 4.12. Cell Attachment Assay

The attachment assay was performed according to [30]. Samples were prepared in twelve replicates; 2 × 10^4^ cells/well were seeded into 96-well plates, either bare or coated with fibronectin (10 µg/mL; Sigma-Aldrich, Saint Louis, MO, USA) or collagen IV (10 µg/mL; Sigma-Aldrich). After 5 min (OAW42) or 15 min (SKOV3) incubation (37 °C, 5% CO_2_ atmosphere), cells were rinsed with PBS. The attachment was evaluated by MTS assay (as described in cell survival assay) and by staining with crystal violet (as described in Matrigel invasion assay). The experiment was repeated three times.

### 4.13. Cell Spreading Assay

The spreading assay was performed according to [30]. Cells were plated at the density of 2 × 10^4^ per well, in 96-well plates. Images were taken at 1.5 h, 2 h and 2.5 h post-plating (objective 20×, eyepiece magnification 10×; AxioVert 40 CFL with digital camera AxioCam Rec 5s, Carl Zeiss, Oberkochen, Germany).

### 4.14. Analysis of the Cell-Cycle Distribution

For the cell cycle assay, cells were plated onto 6-well plates (SKOV3 at the density of 1.5 × 10^5^/well; OAW42 at the density of 1.3 × 10^5^/well; six replicates each) and after 48 h incubation, the cells were collected, washed twice with cold PBS, fixed in cold 70% ethanol and stored at −20 °C. Before experimention, cells were washed with cold PBS and incubated with 100 μg/mL RNase A (Sigma-Aldrich, Saint Louis, MO, USA), then stained with 100 μg/mL propidium iodide solution (Sigma-Aldrich) at 37 °C for 30 min. DNA content was analyzed using fluorescence-activated cell sorting (FACS) with the BD FACS Canto II Cytometer (BD Bioscience, San Jose, CA, USA). A blue laser was used for excitation (488 nm) and the PE channel (586/42 nm) for the detection of DNA stained with iodium propide. Only single cells were considered during cell-cycle distribution analysis. The experiment was repeated two times.

### 4.15. RNA Preparation for Microarrays, Hybridization and Analysis

All procedures were performed according to the manufacturer’s instructions (Affymetrix, Santa Clara, CA, USA) using reagents recommended by Affymetrix. Total RNA (10 ng) from each sample was used as a template for cDNA and subsequent cRNA and 2nd-cycle cDNA syntheses using the GeneChip™ WT Pico Kit (Thermo Fisher Scientific, Waltham, MA, USA). Quality of cRNA was evaluated using 2100 Bioanalyzer (Agilent Technologies, Palo Alto, CA, USA). Twenty µg were taken for 2nd-cycle cDNA synthesis. Subsequently, 5.5 µg of ss-cDNA were fragmented, labeled and hybridized to the GeneChip™ Human Transcriptome Array 2.0 (Affymetrix). Arrays were scanned by GeneChip Scanner 3000 (Affymetrix). All GeneChips were visually inspected for irregularities. The global method of scaling, or normalization, was applied to all GeneChips. Quality measures, likewise the percentage of present genes and the ratio of endogenous genes, indicated a high overall quality of samples and assays.

### 4.16. Microarray Data Analysis

#### 4.16.1. Microarray Preprocessing

Entire bioinformatical analysis was performed in R environment (version 3.5.3) with Bioconductor packages. Microarray data was normalized with robust multi-array average (RMA). Genes with low expression were filtered based on mean expression value histogram. A threshold signal equal to 5, present in more than 3 arrays was assumed, what yielded a total of 22,275 annotated genes. Principal Component Analysis (PCA) was used to assess variability between cell lines, as it proved substantial, further analysis was conducted separately, with result aggregation as the final step.

#### 4.16.2. Differential Expression Analysis

Due to a small number of replicates, differential analysis was performed utilizing moderated *t*-Test. It is a combination of linear models and Empirical Bayes methods which, thanks to a reduced number of hyperparameters, is more stable and better suited for small sample sizes than the classical parametric approaches [38,39]. Separate models were constructed for wild type and pLNCX2 control. Obtained *p*-values were adjusted for multiple testing using Benjamini-Hochberg false discovery rate (FDR) correction. As a large number of differentially expressed genes allowed for strict criteria, adjusted *p*-value of < 10^−4^ was considered statistically significant. For identification of genes common for both cell lines, set intersection was used.

#### 4.16.3. Principal Component Analysis (PCA)

In addition to its value in identifying main differentiating factors and dimensionality reduction, PCA performed by singular value decomposition (SVD) [40] of scaled and centered data can be used as a feature selection method [41]. The result of transformation is a set of linear combinations of initial variables, i.e., gene expression values. Genes contributing most to the difference between groups emerging in PCA can be identified by coefficients greatest in magnitude, with a significance threshold in the form WN^−1/2,^ where N is the number of genes, and W is an arbitrary weight parameter with a recommended value of over 3. For different sample subsets analyzed, gene signatures were selected based on first and second principal component, with W = 3.5.

#### 4.16.4. Gene Set Enrichment Analysis

While the absolute value of SVD coefficients is a valid measure of gene significance, it does not translate directly to *p*-vales. Therefore, pathway enrichment of obtained gene sets was performed by the means of simple Over-Representation Analysis (ORA). Gene groups were tested against Canonical Pathways (CP) available at the Molecular Signatures Database (MsigDB) [42] as a part of curated gene sets (C2) collection, with *p*-value significance threshold of the hypergeometric test equal to 0.05. While Benjamini–Hochberg FDR correction for multiple testing was informatively performed, it was not considered binding due to exploratory character of this part of analysis.

### 4.17. Statistical Analysis

Continuous data were shown as mean values with standard deviation ranges, unless otherwise stated. Data were analyzed using parametric methods depending on data distribution and homogeneity of variance. Univariate statistical significance was determined by one-way analysis of variance (ANOVA) with Scheffe’s adjustment for pairwise comparisons. Difference significance between two groups was determined by two sample *t*-Test for independent samples. The Bonferroni correction was applied for multiple testing. Two-sided *p*-values < 0.05 were considered statistically significant. Analyses were carried out using Statistica 13.1 (TIBCO Software Inc., Palo Alto, CA, USA).

## 5. Conclusions

Our results indicate that higher expression of ITGBL1 in ovarian cancer cells is associated with the features that may worsen the clinical course of the disease. Altered cellular adhesiveness, together with increased motility and invasiveness, may enable easier spreading of cancer cells within peritoneal cavity. Increased resistance to cisplatin and paclitaxel may also account for faster progression of ovarian cancer. These results are in line with our previous observation that ovarian cancer patients with higher ITGBL1 expression in the tumor have significantly shorter overall survival.

Global gene expression analysis revealed that signaling pathways affected by ITGBL1 overexpression were mostly those related to extracellular matrix, cellular communication, migration and integrin signaling; that was consistent with the results of our functional studies.

ITGBL1 role in several human pathologies has recently started to be analyzed, but there are still many questions unanswered and many contradictory results. One reason for these discrepancies are technical constraints (lack of reliably validated anti-ITGBL1 antibodies, poorly described methods precluding repetition of the experiments). Second, ITGBL1 may play diverse roles in different physiological and pathological conditions, and should be studied case by case.

## Figures and Tables

**Table 1:** IC50 values of Cisplatin and Paclitaxel.

Cisplatin		Paclitaxel	
Cell lines	IC50 [µM]	Cell lines	IC50 [µM]
OAW42-ITGBL1	4.256 ± 0.491	OAW42-ITGBL1	0.025 ± 0.011
OAW42-PLNCX2	2.950 ± 0.207	OAW42-PLNCX2	0.008 ± 0.002
SKOV3-ITGBL1	8.066 ± 0.171	SKOV3-ITGBL1	0.201 ± 0.041
SKOV3-PLNCX2	6.612 ± 0.188	SKOV3-PLNCX2	0.114 ± 0.024

**Table 2:** Signaling pathways affected by changed expression of the genes from PC1 (PCA performed on OAW42-PLNCX2 and OAW42-ITGBL1 samples). Only 22 arbitrarily chosen pathways are shown (those related with ECM, integrin signaling, focal adhesion, cellular motility, etc.). Full list of 76 significant pathways is shown in Supplementary Material 5K.

Rank	Pathway	No of Genes in Pathway	No of Genes in Gene Set	*p*-Value
1	KEGG_ECM_RECEPTOR_INTERACTION	84	8	3.59 × 10^6^
4	REACTOME_LAMININ_INTERACTIONS	30	4	0.0003
5	NABA_MATRISOME	1026	24	0.0003
8	PID_INTEGRIN1_PATHWAY	66	5	0.0007
11	NABA_ECM_GLYCOPROTEINS	196	8	0.0014
12	REACTOME_EXTRACELLULAR_MATRIX_ORGANIZATION	301	10	0.0017
17	PID_REELIN_PATHWAY	28	3	0.0034
20	REACTOME_MET_ACTIVATES_PTK2_SIGNALING	30	3	0.0041
22	NABA_ECM_REGULATORS	238	8	0.0045
31	KEGG_FOCAL_ADHESION	199	7	0.0061
33	NABA_MATRISOME_ASSOCIATED	751	16	0.0079
35	REACTOME_DISSOLUTION_OF_FIBRIN_CLOT	13	2	0.0084
36	REACTOME_MET_PROMOTES_CELL_MOTILITY	41	3	0.0099
37	NABA_CORE_MATRISOME	275	8	0.0104
40	PID_INTEGRIN3_PATHWAY	43	3	0.0112
42	REACTOME_INTEGRIN_CELL_SURFACE_INTERACTIONS	85	4	0.0137
47	REACTOME_DEGRADATION_OF_THE_EXTRACELLULAR_MATRIX	140	5	0.0183
57	REACTOME_NON_INTEGRIN_MEMBRANE_ECM_INTERACTIONS	59	3	0.0261
60	PID_INTEGRIN_A9B1_PATHWAY	25	2	0.0297
61	PID_INTEGRIN_CS_PATHWAY	26	2	0.0319
75	PID_INTEGRIN_A4B1_PATHWAY	33	2	0.0494
76	REACTOME_ADHERENS_JUNCTIONS_INTERACTIONS	33	2	0.0494

**Table 3:** Signaling pathways affected by changed expression of the genes from PC1 (PCA performed on SKOV3-PLNCX2 and SKOV3-ITGBL1 samples). Only 44 arbitrarily chosen pathways which are related to ECM, cell junction, cellular motility, ERBB2/ERBB4 signaling, etc. are shown. Full list of 146 significant pathways is shown in Supplementary Material 5O.

Rank	Pathway	No. of Genes in Pathway	No. of Genes in Gene Set	*p*-Value
1	NABA_MATRISOME	1026	35	7.41 × 10^7^
2	REACTOME_EXTRACELLULAR_MATRIX_ORGANIZATION	301	16	4.68 × 10^6^
4	REACTOME_CELL_JUNCTION_ORGANIZATION	92	8	3.96 × 10^5^
7	PID_ERBB_NETWORK_PATHWAY	15	4	4.23 × 10^5^
9	NABA_MATRISOME_ASSOCIATED	751	25	4.58 × 10^5^
16	REACTOME_CELL_CELL_JUNCTION_ORGANIZATION	65	6	0.0003
18	REACTOME_PI3K_EVENTS_IN_ERBB4_SIGNALING	10	3	0.0003
21	REACTOME_CELL_CELL_COMMUNICATION	130	8	0.0004
28	REACTOME_ERBB2_ACTIVATES_PTK6_SIGNALING	13	3	0.0007
29	REACTOME_SHC1_EVENTS_IN_ERBB4_SIGNALING	14	3	0.0008
31	REACTOME_NUCLEAR_SIGNALING_BY_ERBB4	32	4	0.0009
32	NABA_SECRETED_FACTORS	343	13	0.0010
33	REACTOME_ERBB2_REGULATES_CELL_MOTILITY	15	3	0.0010
35	REACTOME_ADHERENS_JUNCTIONS_INTERACTIONS	33	4	0.0010
36	REACTOME_GRB2_EVENTS_IN_ERBB2_SIGNALING	16	3	0.0013
37	REACTOME_PI3K_EVENTS_IN_ERBB2_SIGNALING	16	3	0.0013
44	PID_INTEGRIN1_PATHWAY	66	5	0.0021
49	REACTOME_SHC1_EVENTS_IN_ERBB2_SIGNALING	22	3	0.0033
50	REACTOME_DEGRADATION_OF_THE_EXTRACELLULAR_MATRIX	140	7	0.0033
51	REACTOME_CONSTITUTIVE_SIGNALING_BY_ABERRANT_PI3K_IN_CANCER	75	5	0.0037
55	REACTOME_SIGNALING_BY_EGFR_IN_CANCER	25	3	0.0047
58	NABA_CORE_MATRISOME	275	10	0.0049
59	REACTOME_SIGNALING_BY_ERBB2_IN_CANCER	26	3	0.0053
60	NABA_ECM_REGULATORS	238	9	0.0058
61	REACTOME_INTEGRIN_CELL_SURFACE_INTERACTIONS	85	5	0.0063
62	REACTOME_SIGNALING_BY_PTK6	54	4	0.0064
65	REACTOME_DOWNREGULATION_OF_ERBB2_SIGNALING	29	3	0.0072
69	REACTOME_COLLAGEN_FORMATION	90	5	0.0080
70	REACTOME_SIGNALING_BY_ERBB4	58	4	0.0083
77	REACTOME_TYPE_I_HEMIDESMOSOME_ASSEMBLY	11	2	0.0095
78	KEGG_CELL_ADHESION_MOLECULES_CAMS	133	6	0.0103
87	REACTOME_DISSOLUTION_OF_FIBRIN_CLOT	13	2	0.0133
88	REACTOME_PI3K_AKT_SIGNALING_IN_CANCER	102	5	0.0134
90	PID_ERBB4_PATHWAY	38	3	0.0152
99	REACTOME_NEGATIVE_REGULATION_OF_THE_PI3K_AKT_NETWORK	110	5	0.0180
100	REACTOME_INTERLEUKIN_4_AND_INTERLEUKIN_13_SIGNALING	111	5	0.0186
101	NABA_ECM_GLYCOPROTEINS	196	7	0.0190
103	REACTOME_ECM_PROTEOGLYCANS	76	4	0.0207
104	PID_INTEGRIN3_PATHWAY	43	3	0.0211
109	PID_A6B1_A6B4_INTEGRIN_PATHWAY	46	3	0.0252
113	KEGG_ECM_RECEPTOR_INTERACTION	84	4	0.0286
123	REACTOME_SIGNALING_BY_ERBB2	50	3	0.0313
125	KEGG_ERBB_SIGNALING_PATHWAY	87	4	0.0320
143	PID_INTEGRIN_CS_PATHWAY	26	2	0.0492

**Table 4:** Primers used in this study.

**Primer**	**Sequence (5′→3′)**		**Primer Annealing Temperature**
**Primers used for cloning of the whole *ITGBL1* coding sequence (CDS)**			
F-cloning	AAAAAA*AGATCT*TCCTGCCGCCTCCCTCGGTG		65 °C
R-cloning	CATGGTTTCCTTCGCATTTAT*GTCGAC*TAATGGCCCAG		
**Primers used for concurrent detection of all four *ITGBL1* isoforms**			
F-PCR	GCTCTGGGAGGGGTAAATGTG		59 °C
R-PCR	TGCACTTCCCACAATGACAAGAA		
**Primers used for PCR internal control**			
18S rRNA_L	CATGGCCGTTCTTAGTTGGTG		59 °C
18S rRNA_R	GTGCAGCCCCGGACATCTAA		
**Primers used for detection of different *ITGBL1* mRNA isoforms**			
**Primer Name**	**Sequence (5′→3′**)	**Annealing Temp.**	**mRNA Variant**
w1_F(1)	CCTGTGTGAGTGCCATGAGT	61.4 °C	1 & 2
w2_R	CTTCTGTTTCATCGTCTATGCATTC		
w3_F	TAGTTGCAGTGATGGGAGCA	61.4 °C	3
w2_R	CTTCTGTTTCATCGTCTATGCATTC		
w4_pozaX1_F(2)	CTCTCCACTGAGGGGTTTGG	61.4 °C	4
w1_R(2)	GTGACATGTACCTGCATTAGAGC		

**Table 5:** List of antibodies used in Western blot analysis.

Application	Host/Clonality	Localization of Immunogen	Immunogen Sequence	Source (cat. no)	Dilution
**Primary**					
ITGBL1	Rabbit/Polyclonal	Exon: 6, 7, 8	VCGECTCHDVDPTGDWGDIHGDTCECDERDCRAVYDRYSDDFCSGHGQCNCGRCDCKAGWYGKKCEHPQSCTLSAEESIRKCQGSSDLPCSGRGKCECGKCTCYPPGDRRVYGKTCECDDRRCEDLDGV	Sigma-Aldrich (HPA005676)	1:750
ITGBL1	Rabbit/Polyclonal	Exon: 1 (fragment), 2	MRPPGFRNFLLLASSLLFAGLSAVPQSFSPSLRSWPGAACRLSRAESERR	Thermo Fisher Scientific (PA5-42123)	1:1000
ITGBL1	Rabbit/Polyclonal	Exon: 7 (fragment), 8	PCSGRGKCECGKCTCYPPGDRRVYGKT	ABGENT (Ap8781c)	1:3000
ITGBL1	Rabbit/Polyclonal	Exon: 3 (fragment), 4, 5 (fragment)	CSNAGTCHCGRCKCDNSDGSGLVYGKFCECDDRECIDDETEEICGGHGKC	ProSci (29-712)	1:300
β-actin	Mouse/Monoclonal	------------	------------	Milipore (MAB1501)	1:5000
**Secondary**					
Anti-Rabbit IgG (HRP)	Goat	------------	------------	Millipore (AP132P)	1:1000
Anti-Mouse IgG (HRP)	Donkey	------------	------------	R&D Systems (HAF018)	1:5000

## Supplementary Materials

The following are available online at https://www.mdpi.com/2072-6694/12/9/2676/s1; Supplementary Material 1. Overview of other ITGBL1-related studies; Supplementary Material 2. Semi-quantitative PCR; Supplementary Material 3. Western blotting; Supplementary Material 4. Proliferation; Supplementary Material 5. PCA; Supplementary Material 6. Comparison with Huang et al.; Supplementary Material 7. Antibodies.
