# Supplementary material for: Evaluation of the Role of ITGBL1 in Ovarian Cancer
Source: Cancers (Basel). 2020 Sep 19;12(9):2676. doi: 10.3390/cancers12092676 (PMC7563769; doi:10.3390/cancers12092676)
Supplement: Supplementary file 1 [file cancers-12-02676-s001.zip › Suplement ITGBL1 10.09.2020/1. Supplementary Material 1. Overview.docx]

**Supplementary Material 1. Overview of other ITGBL1-related studies.**

|  | **Pathology** | **Results and conclusions** | **Comments** |
| --- | --- | --- | --- |
| 1 | Ovarian cancer (OC)  Sun et al, 2016 [1] | - ITGBL1 promotes OC cell migration and adhesion (loss and gain of function assays). - Addition of rITGBL1 promotes cell migration and adhesion in a concentration dependent manner. - ATF2 activity was significantly inhibited in ITGBL1-silenced Caov-4 cells, and significantly induced in ITGBL1 overexpressing SKOV3 and rITGBL1 treated ES2 (as compared to controls). On this basis, Authors conclude that ITGBL1 influences the activity of Wnt/PCP pathway. - Reduced levels of phosphorylated FAK and Src were observed in ITGBL1 knocked-down cells (conclusion – ITGBL1 affects FAK/Src pathway in vitro). - RhoA activity was elevated in dose-dependent manner by addition of rITGBL1 - ITGBL1 was highly expressed in ovarian cancer as compared to normal ovarian tissue - Conclusions: ITGBL1 mediated cell migration and adhesion may be due to activation of the Wnt/PCP and FAK/SRC signaling | - We observed lesser initial adhesiveness (5-15 min after seeding; adhesion assay), while enhanced spreading of cells between 1.5-2.5 hours after seeding (spreading assay) - Sun et al performed adhesion assay 1 hour after seeding – they observed that ITGBL1 enhanced cellular attachment - Although we were using different Ab (Sigma-Aldrich) we were not able to detect ITGBL1 in cellular lysates, as authors did; we only detected ITGBL1 in culture medium - In our pathologist’s opinion samples described by Sun et al., as “normal” (Fig. 1C) resemble cancer tissue |
| 2 | Ovarian cancer  (OC)  Song et al, 2020 [2] | - Reduced levels of phosphorylated AKT, GSK-3 and FOXO3 were observed in ITGBL1-downregulated OC cells and in cells treated with LY294002, a broad-spectrum inhibitor of PI3K. ITGBL1 overexpression and addition of rITGBL1 had the opposite effect. Conclusion: ITGBL1 activates PI3K/Akt signaling. - ITGBL1 promoted tumor cell resistance to chemotherapeutic drugs, both in vitro and in vivo: - ITGBL1 knock-down promoted cisplatin and paclitaxel induced apoptosis, while ITGBL1 overexpression and addition of rITGBL1 - reduced. - ITGBL1 expression was significantly upregulated in ovarian cancer tissues compared to that in adjacent non-cancer tissues and high expression of ITGBL1 was significantly associated with lymph node invasion and advanced FIGO stage. - Conclusion: ITGBL1 confers tumor cell chemoresistance both in vitro and in vivo via upregulatingPI3K/Akt signaling. | - In our analysis increased ITGBL1 mRNA expression was a negative prognostic factor, no correlation with FIGO stage was observed (however, we had only FIGO III and IV samples). - Using the same Ab (Sigma-Aldirch), we were able to detect ITGBL1 only in culture media, while not in cell lysates. |
| 3 | Colorectal cancer (CRC)  Qiu et al, 2018 [3] | - Knockdown of ITGBL1 inhibits CRC cell proliferation, migration and invasion. - GO analysis revealed that ITGBL1 was associated with cell adhesion. - GSEA indicated that ITGBL1 was enriched in ECM receptor interaction and focal adhesion. According to the results of GSEA, Authors speculate that ITGBL1 might promote CRC migration and invasion through the FAK signaling pathway - ITGBL1 was significantly up-regulated in CRC (182 primary CRC tissues) as compared to normal colon tissues (54 samples). - High ITGBL1 expression was associated with shortened survival. - ITGBL1 was proposed as a novel oncogene associated with the progression and prognosis of CRC. | In the abstract Authors wrote that ITGBL1 KD promotes proliferation, migration and invasion:    while in the results - opposite:   |
| 4 | Gastric cancer (GC)  Li et al, 2017 [4] | - By analyzing Gene Expression Omnibus (GEO) and Oncomine databases, ITGBL1 was significantly up-regulated in GC, as compared with normal controls. Elevated ITGBL1 expression was positively correlated with node-metastasis and distant metastasis stage. High ITGBL1 expression was significantly associated with shorter survival in GC patients (independent prognostic factor) - Gene set enrichment analysis (GSEA) of multiple GEO datasets revealed a close relationship between ITGBL1 expression and the KRAS/EMT signaling pathway. - ITGBL1 as a potential biomarker for prognosis of gastric cancer patients; may influence cancer cell metastasis and invasion through the KRAS/EMT signaling pathways |  |
| 5 | Lung cancer  Gan et al., 2015 [5] | - ITGBL1 mRNA is downregulated in NSCLC tissue in comparison to tumor adjacent normal tissue (48 paired samples). - Low level of ITGBL1 mRNA correlates with poor prognosis in NSCLC (Oncomine database; opposite to other cancers); - rITGBL1 inhibits cellular migration and invasion in vitro (opposite to other cancers), - Downregulated ITGBL1 enhances Wnt/PCP signaling (deduced on the basis of ATF2 activity – increased in ITGBL1 knocked-down cells and decreased under rITGBL1 treatment). - Downregulation of ITGBL1 is mediated by miR-576-5p - Conclusion: ITGBL1 is proposed as a novel tumor suppressor in NSCLC. Down-regulation of ITGBL1 was linked with promotion of cell invasion through the Wnt/PCP signaling pathway | - This study suggests that ITGBL1 has a tumor suppressor activity in NSCLC, opposite to other cancers |
| 6 | Pulmonary fibrosis  Song et al, 2019 [6] | - RNA sequencing, chromatin immunoprecipitation (ChIP)-qPCR, CRISPR-Cas9 technology, and promoter activity analysis showed that the fibrotic function of a novel lncRNA - lncITPF depends on its host gene ITGBL1, but they did not share the same promoter and were not co-transcribed. - RNA-protein pull-down, liquid chromatography-mass spectrometry (LC-MS), and protein-RNA immunoprecipitation showed that lncITPF regulated H3 and H4 histone acetylation in the ITGBL1 promoter by targeting heterogeneous nuclear ribonucleoprotein L. | - On western blots ITGBL1 is indicated at a molecular mass of 88 kDa (Abcam Ab, no cat#) |
| 7 | Liver fibrosis Wang et al, 2017 [7] | - Gene expression profile analysis in HBV-related liver fibrosis samples indicated ITGBL1 as a most significantly associated with disease severity (Liver biopsy from 136 chronically HBV-infected patients). - GSEA indicated that TGFβ signaling and epithelial mesenchymal transition (EMT) played important roles in initiating and promoting fibrotic progression. - The expression of TGFβ1 was significantly upregulated in hepatoma cell lines with an increasing amount of transfected ITGBL1 plasmid. Conditioned medium from these cells significantly up-regulated TGF-β and α-SMA expression in hepatocyte stellate cells activation. - Conclusion: ITGBL1 is an upstream regulator of liver fibrosis via upregulation of TGF β1. |  |
| 8 | Liver cirrhosis/ chronic hepatitis, Xu et al, 2016 [8] | - Microarray analysis of 40 samples from patients with chronic hepatitis B revealed six-gene signature (ITGBL1, CD24, CXCL6, EHF, LUM and SOX9) able to predict the progression of cirrhosis. - ITGBL1 highly overexpressed in stage 4 cirrhosis as compared to lower stages. |  |
| 9 | Hepatocellular carcinoma (HCC)  Huang et al, 2020 [9] | - ITGBL1 expression (WB) is increased in HCC as compared to the adjacent normal tissues (22 pairs of tumor and adjacent non-tumor tissues). - Higher ITGBL1 expression is significantly related with shorter survival (60 HCC patients). - ITGBL1 overexpression promote HCC cells migration and invasion in vitro and in vivo. - ITGBL1 overexpression stimulates the EMT (increased expression of KRT17, Snail, Slug, and Vimentin, and the downregulation of Claudin-1) in HCC cells - ITGBL1 overexpression stimulates the TGF-β/Smad signaling pathway, while the inhibition of the TGF-β/Smad signaling pathway downregulates the ITGBL1-mediated EMT. | - Continuation of the work by Wang et al, 2017 (ITGBL1 presumed important on the basis of previous gene expression profiling experiment) - RNA-seq – comparison of *ITGBL1*-overexpressing and control cell line |
| 10 | Prostate cancer (PC)  Li et al, 2019 [10] | - ITGBL1 is upregulated in PC tissues as compared to the adjacent normal tissue (WB) - ITGBL1 expression level associated with lymph node mets, Gleason score, and stage (174 PC tissues). - Upregulation of ITGBL1 enhances invasion, migration, and EMT in PCa cells. Downregulating ITGBL1 exhibited an opposite effect. - ITGBL1 stimulates NF-kB signaling (luciferase assay) - Cellular invasion and migration stimulated by ITGBL1 was abrogated by NF-κB signaling inhibitors. | - In opinion of our pathologist, image described as non-lymph nodes metastatic prostate cancer (non-LNM) resembles normal prostate tissue (small, regular nuclei, normal nucleus-to-cytoplasm ratio, etc.) IHC Ab: not defined in Methods section, HE-stained tissue sections not shown |
| 11 | Breast cancer  (BC)  Li et al., 2015 [11] | - In breast cancer ITGBL1 is co-expressed with genes related to bone remodeling and bone metastasis: osteoblast specific transcription factor Runx2, cadherins, integrins, collagens, osteoblast-specific factor 2 (OSF-2), osteoglycin (OGN), TGFβ3 and bone morphogenetic protein 1 (BMP1). - High ITGBL1 expression in primary tumor is related with bone metastases (RT-PCR, 88 BC samples). - ITGBL1 expressing BC cells have equal tumorigenicity as control cells but generate higher number of bone metastasis in nude mice. - ITGBL1 is engaged in TGFβ signaling (ITGBL1 increased the expression and secretion of TGFβ1 and TGFβ3 in BC cells, inhibition of the TGFβ pathway by SB431542 weakened the ITGBL1-induced bone metastatic potential of BC cells). - ITGBL1 expression is regulated by Runx2 transcription factor. ITGBL1 positively regulated proliferation-related genes KI67 and CCNE, suggesting that ITGBL1 can confer proliferation advantage to tumor cells in the bone microenvironment. - Conclusion: bone metastases driven by ITGBL1 may result from activation of the TGFβ signaling pathway | - We could not find description of WB methodology, WB Ab not indicated. |
| 12 | Cartilage/ osteoarthritis (OA)  Song et al., 2018 [12] | - *Itgbl1* is predominantly and temporally expressed in chondrogenic precartilage in Xenopus (RNA-seq experiments, in-situ hybridization). - ITGBL1 depletion inhibits cartilage-specific ECM deposition in Xenopus embryos. - ITGBL1 function is necessary for chondrogenic differentiation of human bone marrow–derived mesenchymal stem cells. - ITGBL1 inhibits integrin-ECM interactions (prostate cancer cells PC3 with siRNA silenced ITGBL1). - ITGBL1 promotes chondrogenesis via integrin inactivation. - ITGBL1 is secreted and physically interacts with integrins to down-regulate their activity. - ITGBL1 depletion results in OA-like cartilage damage in knee joints, ITGBL1 overexpression protects cartilage tissue from OA development (mouse model). - Conclusion: ITGBL1 functions as an inhibitor of integrin-ECM interaction that is critical for both cartilage formation and OA development. | - Physical interactions between ITGBL1 and integrins - Important: Binding was observed in the presence, but not in the absence of CA2+ |
| 13 | CRC  Ji et al., 2020 <https://www.nature.com/articles/s41467-020-14869-x.pdf> [13] | - Primary CRC tumors release ITGBL1-rich extracellular vesicles (EVs) to the circulation to activate resident fibroblasts in remote organs. - ITGBL1-enriched EVs stimulate the TNFAIP3-mediated NF-κB signaling pathway to activate fibroblasts. Activated fibroblasts produce pro-inflammatory cytokines to promote metastatic cancer growth. - Genetic gain and loss-of-function experiments showed that RUNX2 may regulate ITGBL1 expression - Co-IP and immunoblot experiments discovered TNFAIP3 as a strong binding partner of ITGBL1 in WI-38 and LX-2 cells. Immunofluenrescence analysis showed co-location of ITGBL1 and TNFAIP3 - Co-IP in combination with LC-MS/MS (or with western blot) showed no direct binding between ITGBL1 and integrins. - KD of integrin β1 barely affected the regulatory effect of ITGBL1 on the NF-κB signaling pathway. - Silencing of TNFAIP3 decreased ITGBL1-induced phosphorylation of inhibitor of IKKα/β, and phosphorylated NF-κB, but increased IκBα. Luciferase test confirmed the regulatory effect of TNFAIP3 on the NF-κB signaling pathway. - Conclusion: fibroblasts become activated via the ITGBL1-TNFAIP3-NF-κB signaling pathway. | - No direct interactions between ITGBL1 and integrins - Our note: possibly due to absence of Ca2+ ions! |
| 14 | CRC  Qi et al. 2020 <https://www.ncbi.nlm.nih.gov/pmc/articles/PMC7076154/pdf/fonc-10-00259.pdf> [14] | - ITGBL1 expression was increasing with the development of CRC (higher expression in higher stages). - Gene Set Enrichment Analysis (GSEA) indicated that a high expression of ITGBL1 in CRC was linked to Wnt signaling pathway, cell polarity, and tissue development. - Low expression of ITGBL1 was related to cellular respiration, electron transfer chain, and oxidative phosphorylation. - An interaction network of ITGBL1 and Wnt signaling proteins was constructed and revealed that β-catenin interacted with multiple extracellular Wnt signals and could bind to ITGBL1. - ITGBL1 in CRC may affect extracellular Wnt signals via β-catenin. | - Entirely computational study |

1. Sun, L.; Wang, D.F.; Li, X.T.; Zhang, L.L.; Zhang, H.; Zhang, Y.J. Extracellular matrix protein ITGBL1 promotes ovarian cancer cell migration and adhesion through Wnt/PCP signaling and FAK/SRC pathway. *Biomed Pharmacother* **2016**, *81*, 145-151, doi:10.1016/j.biopha.2016.03.053.

2. Song, J.D.; Yang, P.X.; Lu, J.J. Upregulation of ITGBL1 predicts poor prognosis and promotes chemoresistance in ovarian cancer. *Cancer Biomark* **2020**, *27*, 51-61, doi:10.3233/Cbm-190460.

3. Qiu, X.; Feng, J.R.; Qiu, J.; Liu, L.; Xie, Y.; Zhang, Y.P.; Liu, J.; Zhao, Q. ITGBL1 promotes migration, invasion and predicts a poor prognosis in colorectal cancer. *Biomed Pharmacother* **2018**, *104*, 172-180, doi:10.1016/j.biopha.2018.05.033.

4. Li, R.K.; Zhuang, C.; Jiang, S.H.; Du, N.; Zhao, W.Y.; Tu, L.; Cao, H.; Zhang, Z.G.; Chen, X.F. ITGBL1 Predicts a Poor Prognosis and Correlates EMT Phenotype in Gastric Cancer. *J Cancer* **2017**, *8*, 3764-3773, doi:10.7150/jca.20900.

5. Gan, X.; Liu, Z.; Tong, B.; Zhou, J. Epigenetic downregulated ITGBL1 promotes non-small cell lung cancer cell invasion through Wnt/PCP signaling. *Tumour biology : the journal of the International Society for Oncodevelopmental Biology and Medicine* **2015**, 10.1007/s13277-015-3919-8, doi:10.1007/s13277-015-3919-8.

6. Song, X.D.; Xu, P.; Meng, C.; Song, C.G.; Blackwell, T.S.; Li, R.R.; Li, H.B.; Zhang, J.J.; Lv, C.J. lncITPF Promotes Pulmonary Fibrosis by Targeting hnRNP-L Depending on Its Host Gene ITGBL1. *Mol Ther* **2019**, *27*, 380-393, doi:10.1016/j.ymthe.2018.08.026.

7. Wang, M.; Gong, Q.; Zhang, J.; Chen, L.; Zhang, Z.; Lu, L.; Yu, D.; Han, Y.; Zhang, D.; Chen, P., et al. Characterization of gene expression profiles in HBV-related liver fibrosis patients and identification of ITGBL1 as a key regulator of fibrogenesis. *Sci Rep* **2017**, *7*, 43446, doi:10.1038/srep43446.

8. Xu, M.Y.; Qu, Y.; Li, Z.; Li, F.; Xiao, C.Y.; Lu, L.G. A 6 gene signature identifies the risk of developing cirrhosis in patients with chronic hepatitis B. *Frontiers in bioscience* **2016**, *21*, 479-486.

9. Huang, W.; Yu, D.; Wang, M.; Han, Y.; Lin, J.; Wei, D.; Cai, J.; Li, B.; Chen, P.; Zhang, X. ITGBL1 promotes cell migration and invasion through stimulating the TGF-beta signalling pathway in hepatocellular carcinoma. *Cell Prolif* **2020**, *53*, e12836, doi:10.1111/cpr.12836.

10. Li, W.Z.; Li, S.R.; Yang, J.; Cui, C.Y.; Yu, M.; Zhang, Y.D. ITGBL1 promotes EMT, invasion and migration by activating NF-kappa B signaling pathway in prostate cancer. *Oncotargets Ther* **2019**, *12*, 3753-3763, doi:10.2147/Ott.S200082.

11. Li, X.Q.; Du, X.; Li, D.M.; Kong, P.Z.; Sun, Y.; Liu, P.F.; Wang, Q.S.; Feng, Y.M. ITGBL1 Is a Runx2 Transcriptional Target and Promotes Breast Cancer Bone Metastasis by Activating the TGF beta Signaling Pathway. *Cancer research* **2015**, *75*, 3302-3313, doi:10.1158/0008-5472.Can-15-0240.

12. Song, E.K.; Jeon, J.; Jang, D.G.; Kim, H.E.; Sim, H.J.; Kwon, K.Y.; Medina-Ruiz, S.; Jang, H.J.; Lee, A.R.; Rho, J.G., et al. ITGBL1 modulates integrin activity to promote cartilage formation and protect against arthritis. *Sci Transl Med* **2018**, *10*, doi:ARTN eaam7486

10.1126/scitranslmed.aam7486.

13. Ji, Q.; Zhou, L.H.; Sui, H.; Yang, L.; Wu, X.N.; Song, Q.; Jia, R.; Li, R.X.; Sun, J.; Wang, Z.Y., et al. Primary tumors release ITGBL1-rich extracellular vesicles to promote distal metastatic tumor growth through fibroblast-niche formation. *Nature Communications* **2020**, *11*, doi:10.1038/s41467-020-14869-x.

14. Qi, L.; Song, F.Y.; Ding, Y.Q. Regulatory Mechanism of ITGBL1 in the Metastasis of Colorectal Cancer. *Front Oncol* **2020**, *10*, doi:ARTN 259

10.3389/fonc.2020.00259.
