# Supplementary material for: Evaluation of the Role of ITGBL1 in Ovarian Cancer
Source: Cancers (Basel). 2020 Sep 19;12(9):2676. doi: 10.3390/cancers12092676 (PMC7563769; doi:10.3390/cancers12092676)

**Supplementary Material 2. RT-PCR detection of different ITGBL1 mRNA isoforms**

**in wild-type and genetically modified cell lines – unprocessed gel images**

**A and B – detection of variant 1 and 2 mRNAs.** A – original gel, B – the same gel with indicated areas that were cropped and included in Figure 1C (main text)**.**

**A.**

**B.**


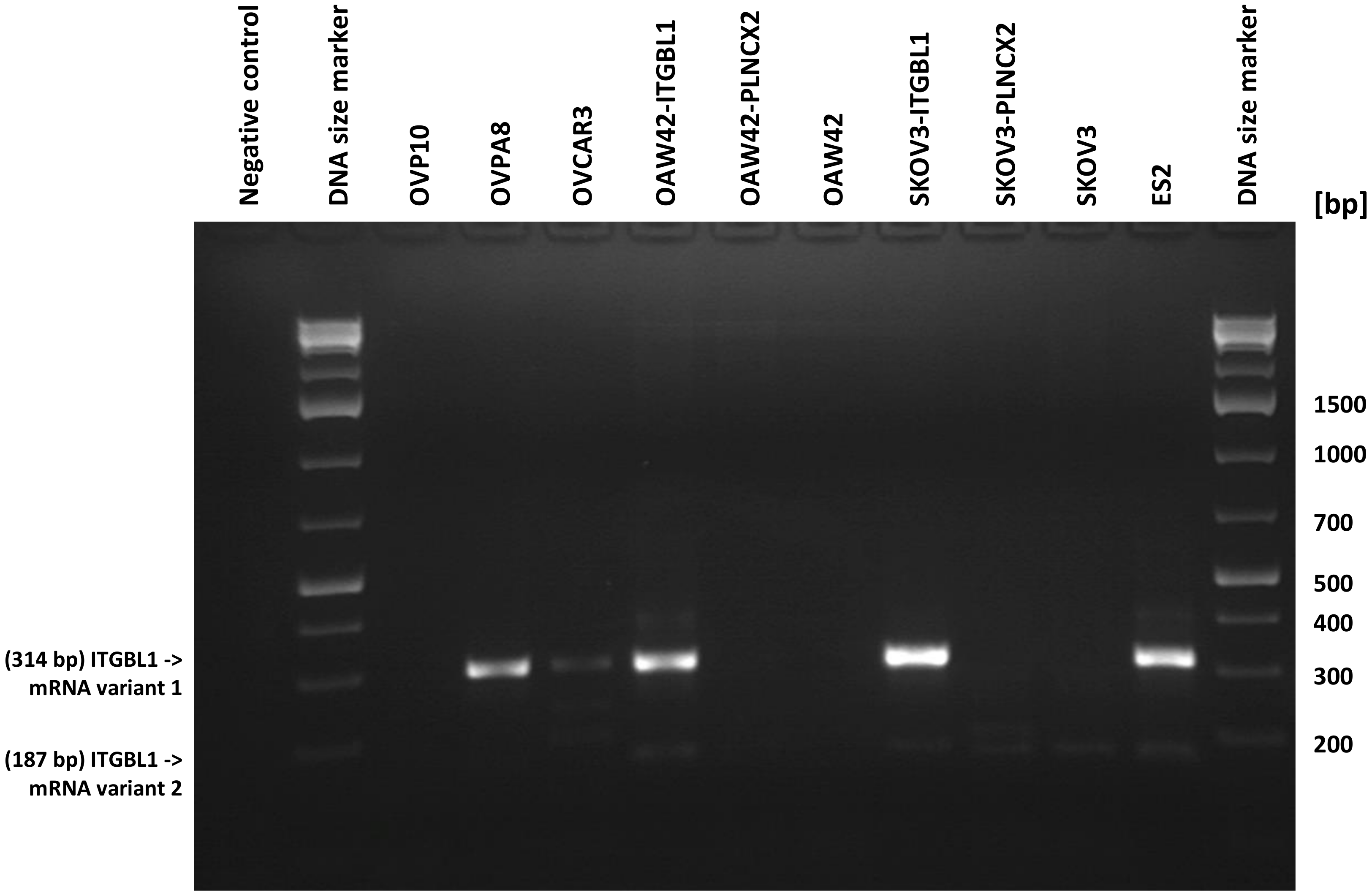

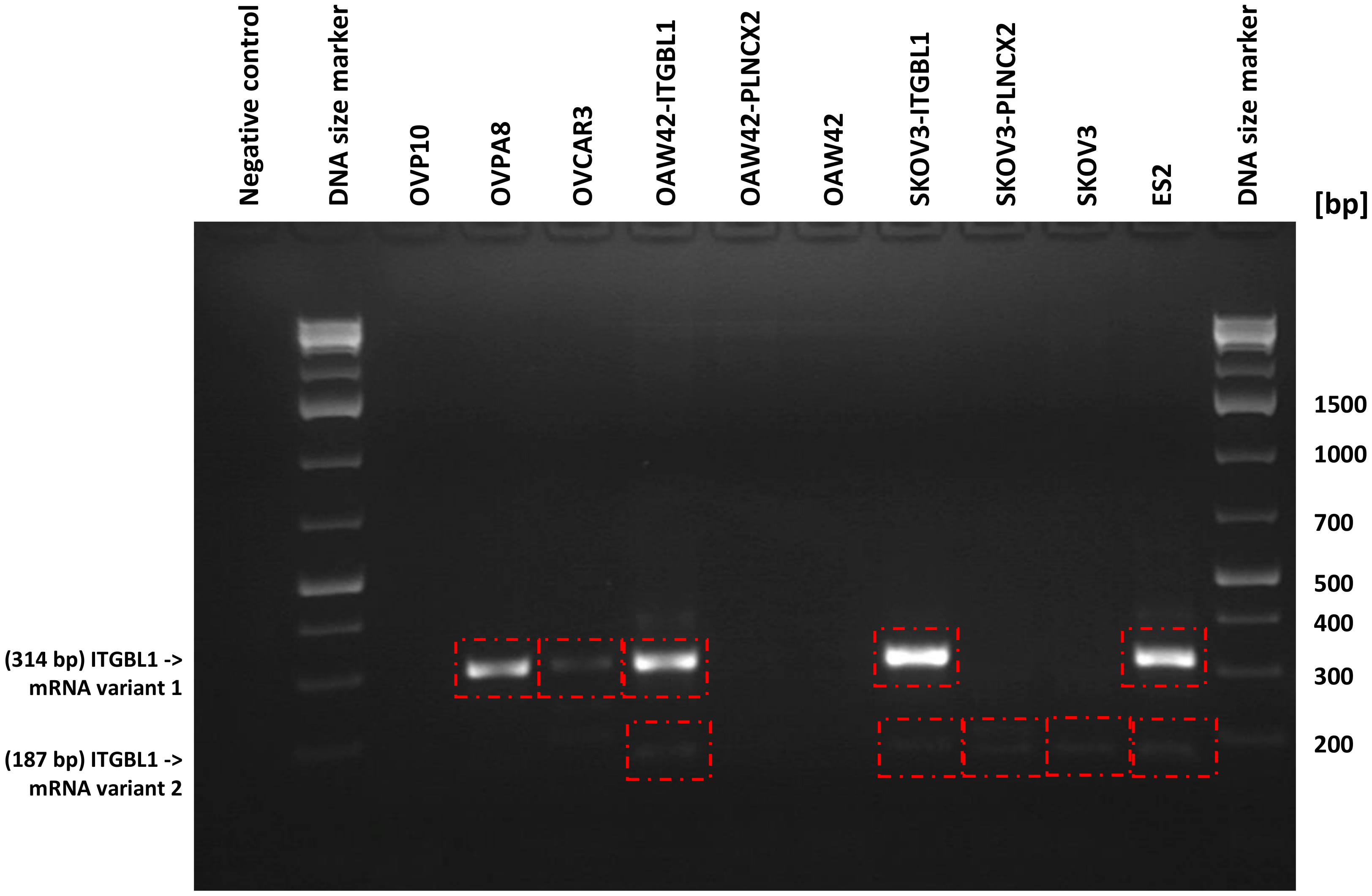


**C and D –** **detection of** **variant 1 and 2 mRNAs together with 18S rRNA (reference).** Due to similar size, 18S rRNA and ITGBL1 variant 2 mRNA could not be distinguished.

**D.**


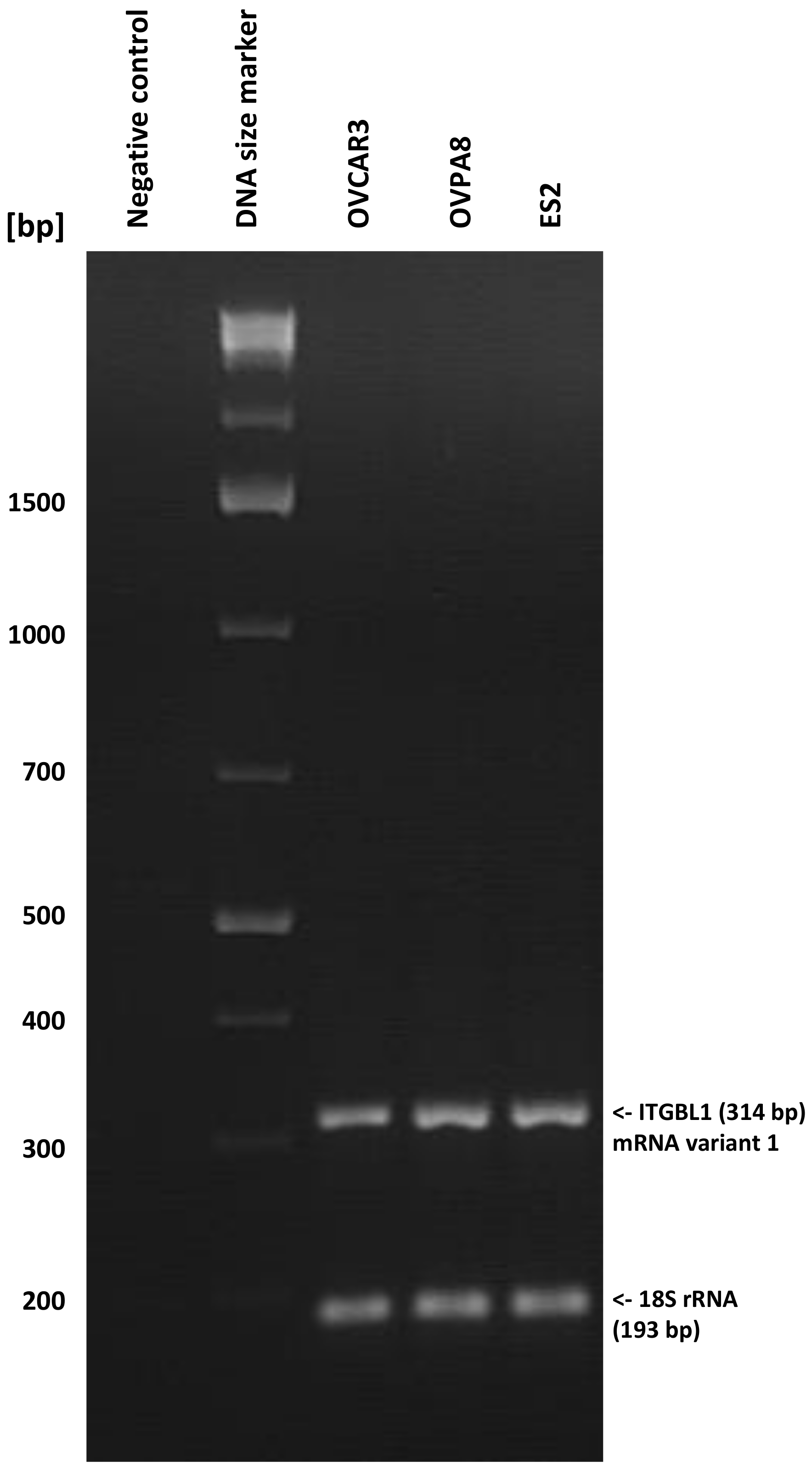


**C.**


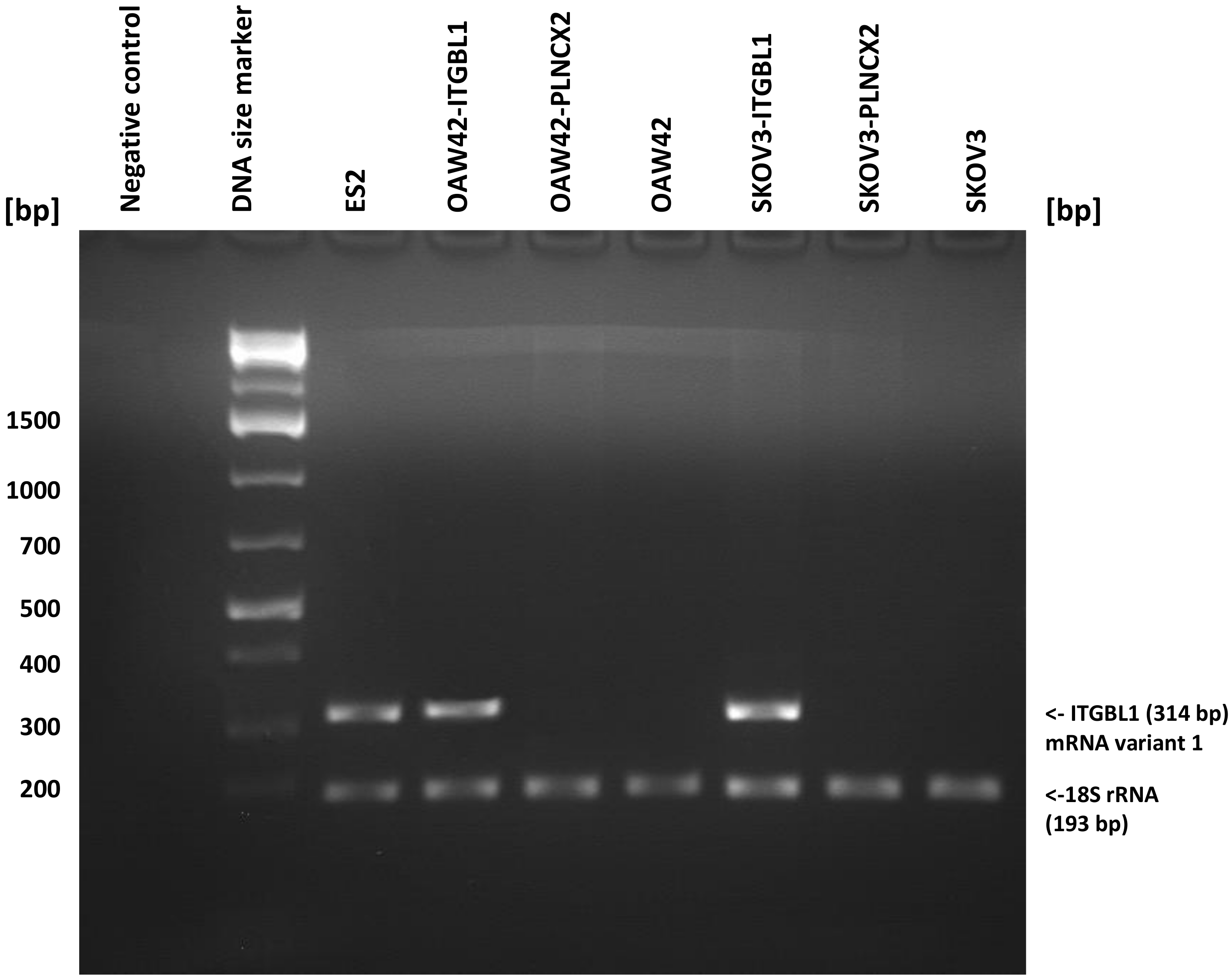


**E -** concurrent amplification of all ITGBL1 mRNA isoforms in wild-type and genetically modified cell lines included in Figure 2B (main text).


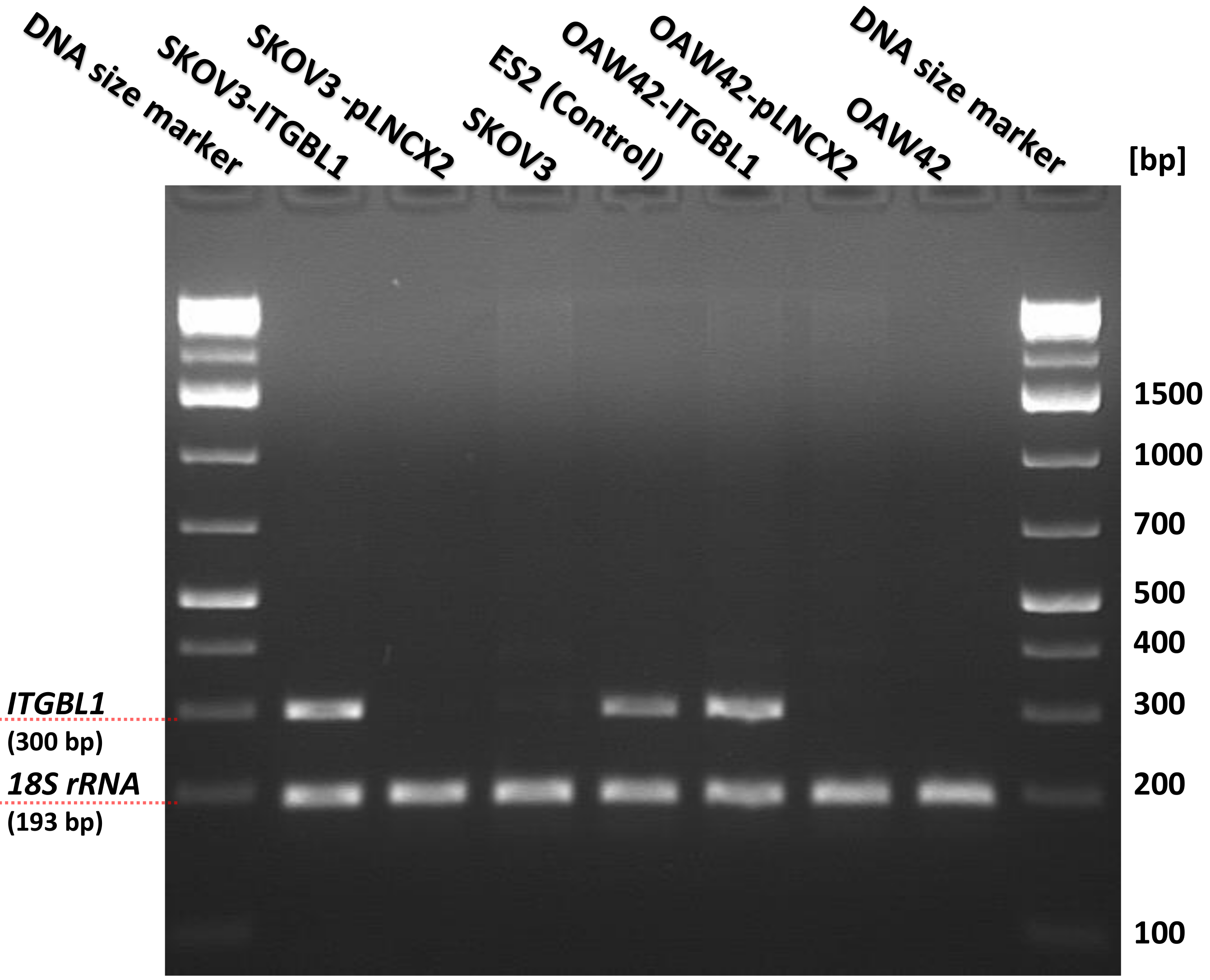


**E.**

**F and G - detection of variant 3 mRNA.** F – original gel, G – the same gel with indicated areas that were cropped and included in Figure 1C (main text)**.**

**F.**

**G.**


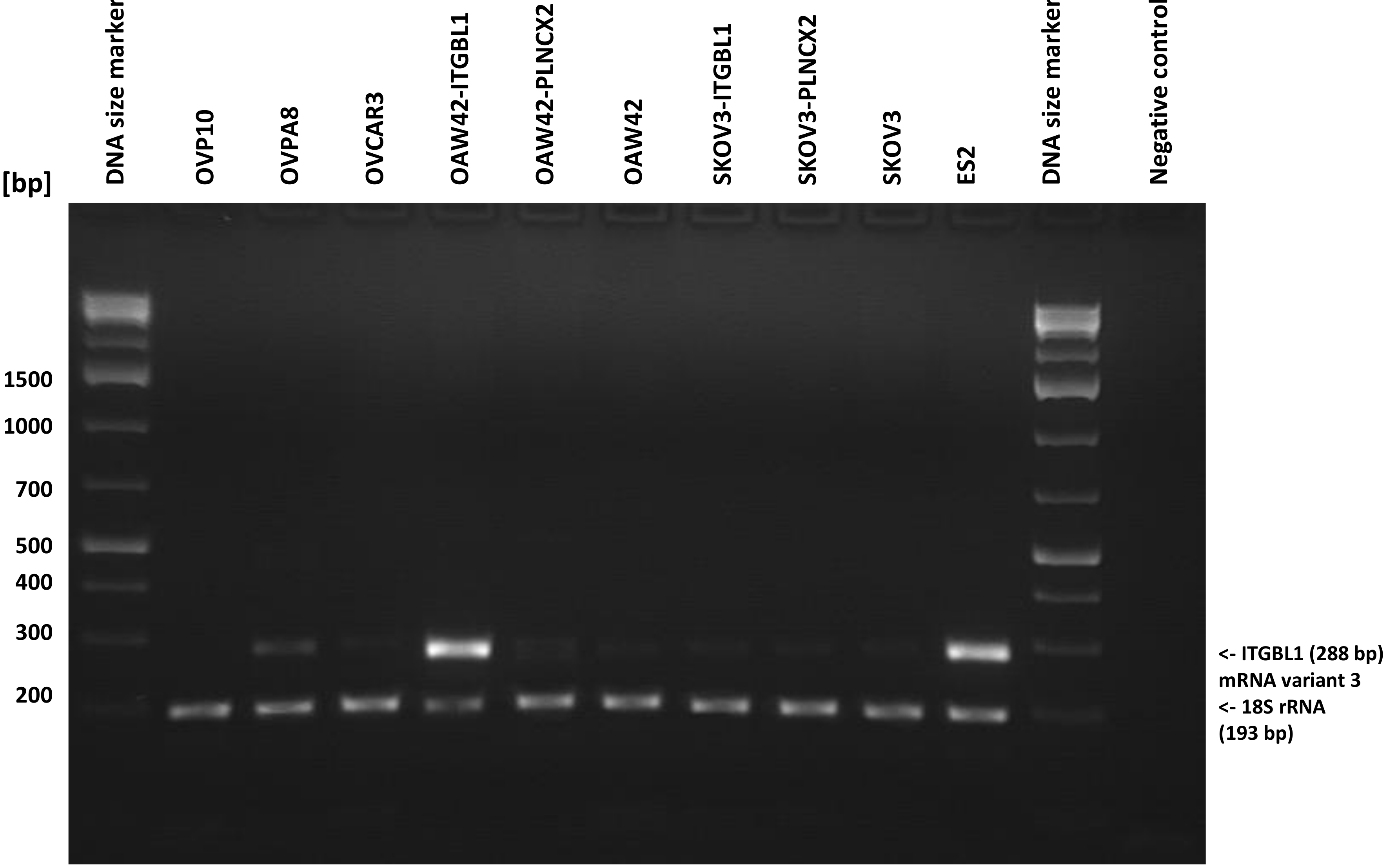

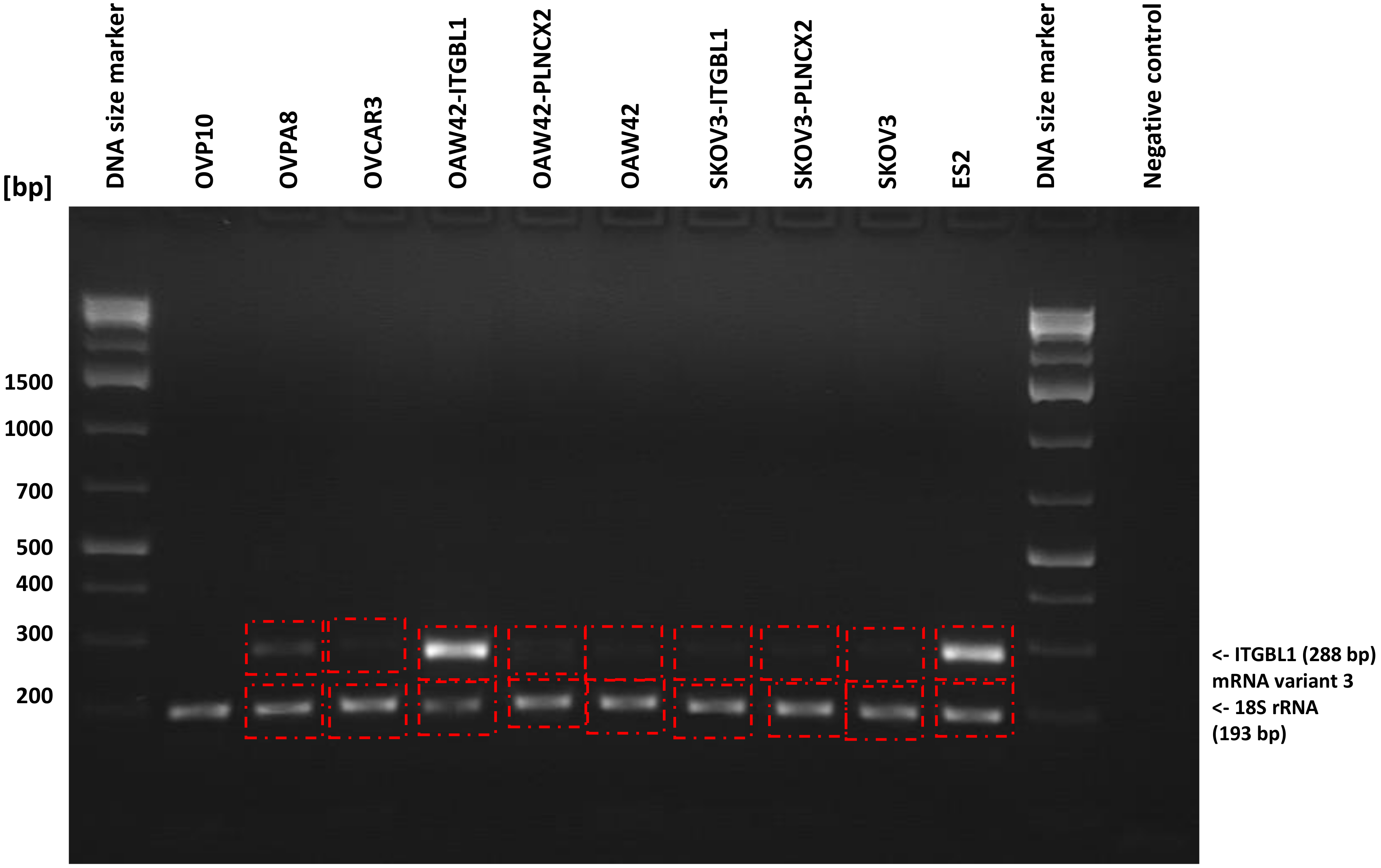


**H and I - detection of variant 4 mRNA.** H – original gel, I – the same gel with indicated areas that were cropped and included in Figure 1C (main text)**.**

**I.**

**H.**


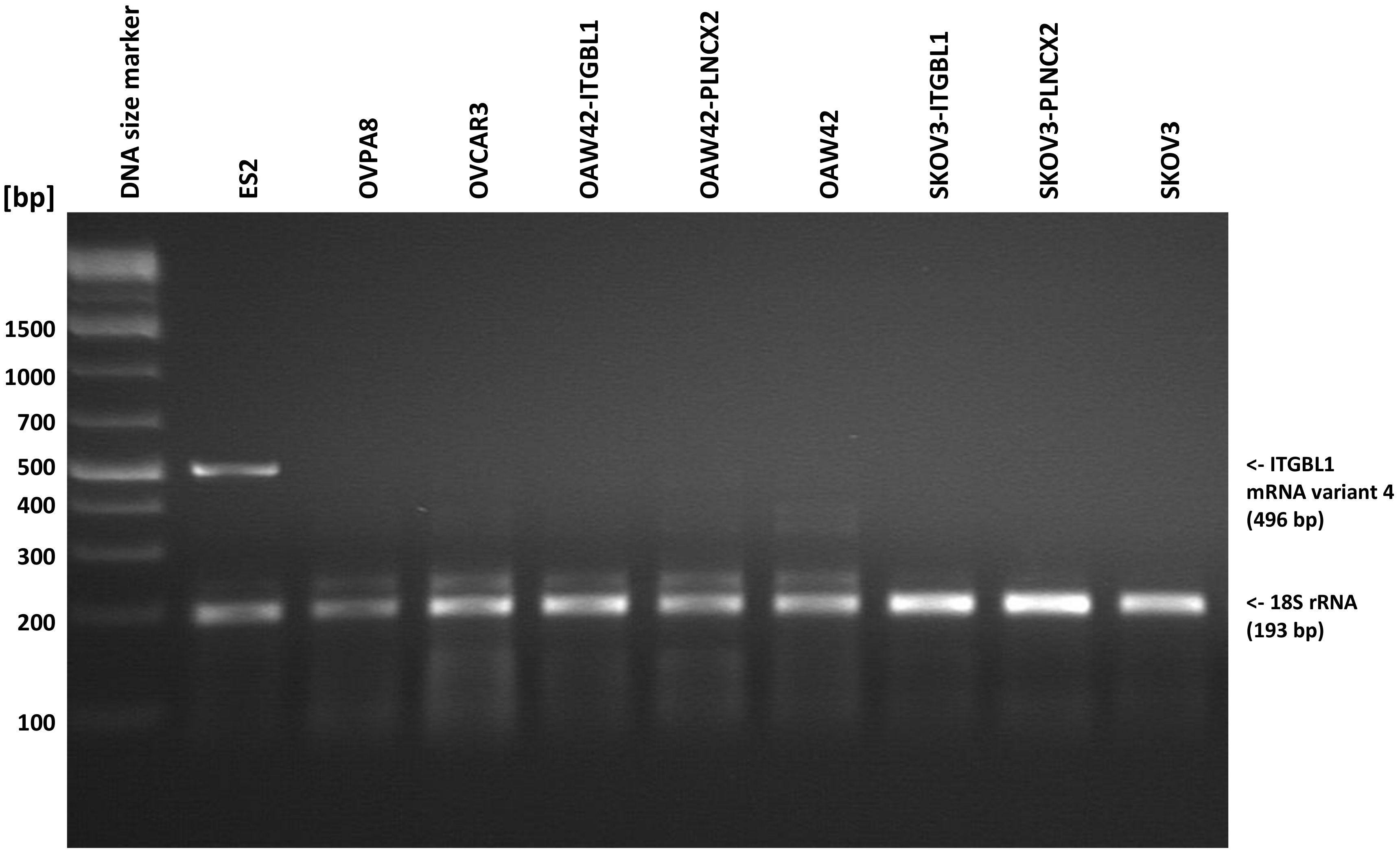

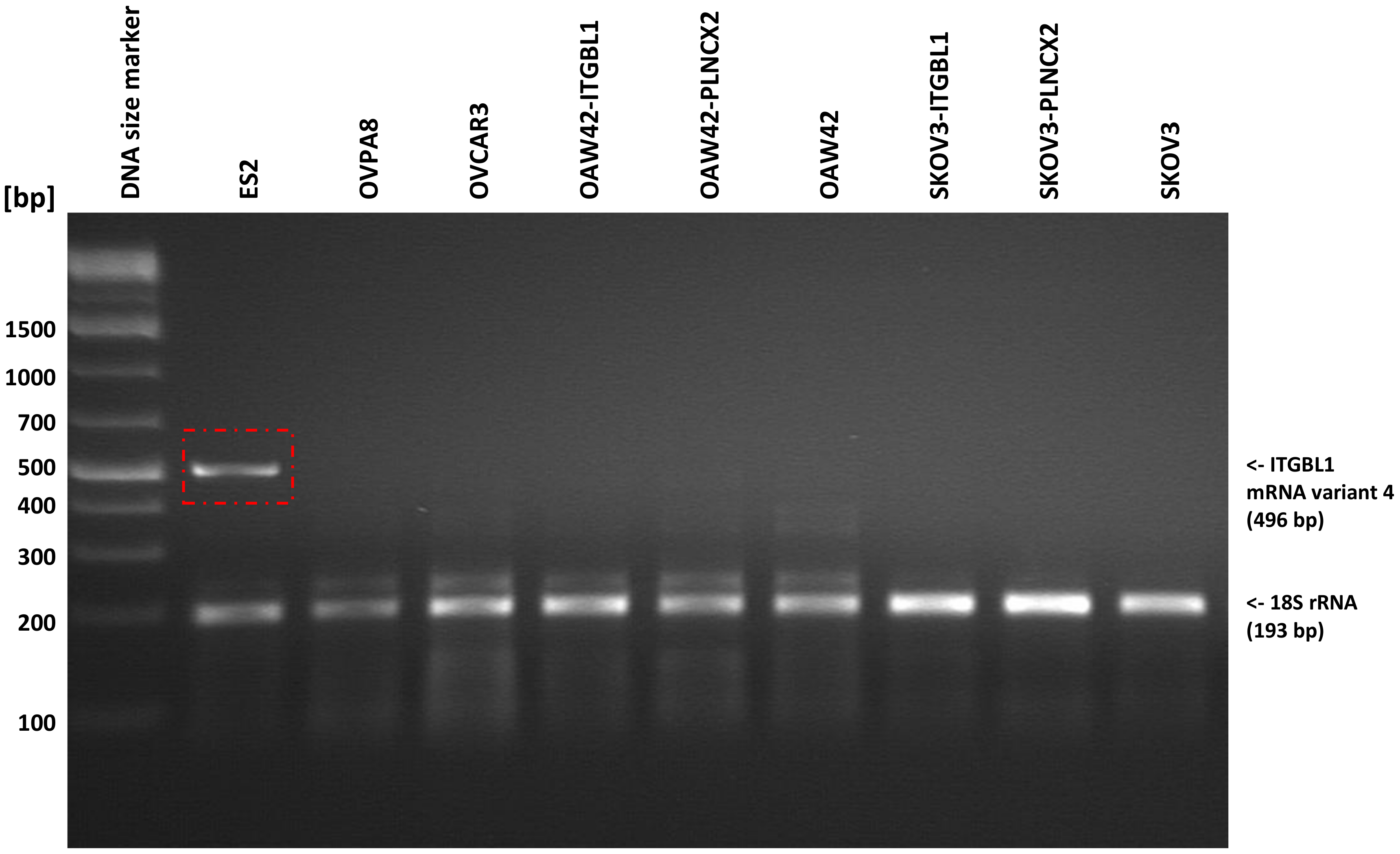

Supplement: Supplementary file 1 [file cancers-12-02676-s001.zip › Suplement ITGBL1 10.09.2020/2. Supplementary Material 2. Semi-quantitative PCR.docx]
