# Supplementary material for: Evaluation of the Role of ITGBL1 in Ovarian Cancer
Source: Cancers (Basel). 2020 Sep 19;12(9):2676. doi: 10.3390/cancers12092676 (PMC7563769; doi:10.3390/cancers12092676)

**Supplementary Material 3. Western blotting:**

**WB results & unprocessed original autoradiograms (immunoblots)**

**Supplementary Material 3A. Western blot detection of ITGBL1 with different antibodies:** (**A**, **B**) **Sigma-Aldrich** (cat. no HPA005676; Saint Louis, MO, USA) (antibody dilution – 1:750. Protein amount per sample – 50 µg), (**C**) **ProSci** (cat. no 29-712; Poway, CA, USA) (antibody dilution – 1:300. Protein amount per sample – 50 µg), (**D**) **ABGENT** (cat. no Ap8781c; San Diego, CA, USA) (antibody dilution - 1:300. Protein amount per sample - 50 µg), (**E**) **Thermo Fisher Scientific** (cat. no PA5-42123; Waltham, MA, USA) (antibody dilution - 1:1000. Protein amount per sample - 50 µg). (**C**, **D**, **E**) With all three antibodies (C, D, E) we were unable to obtain a band corresponding to ITGBL1 (predicted size - 54 kDa). With Sigma-Aldrich HPA005676 antibody we could detect ITGBL1 in the concentrated culture medium (B) but not in whole-cell extracts (A). Based on RT-PCR results ES2 cell line producing the highest levels of *ITGBL1* mRNA was used as a positive control.


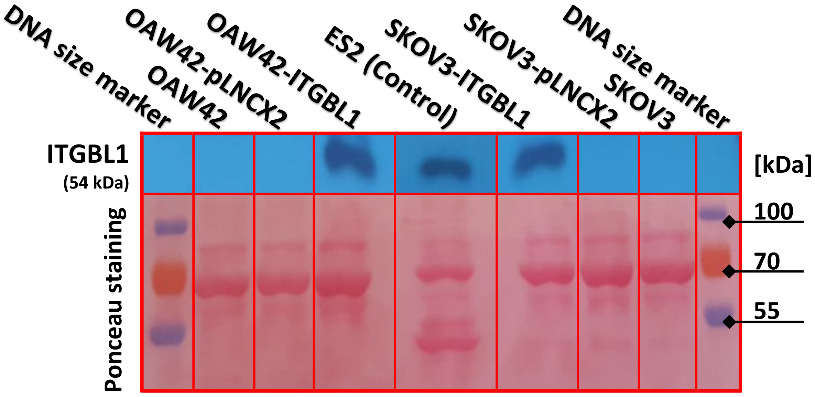


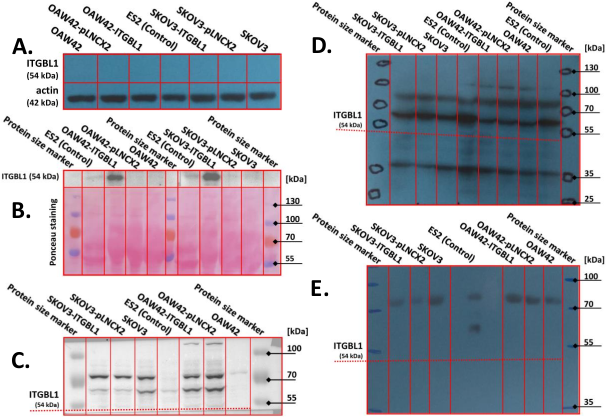


**Supplementary Material 3B. Unprocessed original autoradiograms (immunoblots)** (**A**, **C**, **E**) included in Figure 2C and D as well (**A**, **C-G**) in Supplementary Material 3A. (**B**) Original scan of immunoblot A showing distribution of protein size marker.


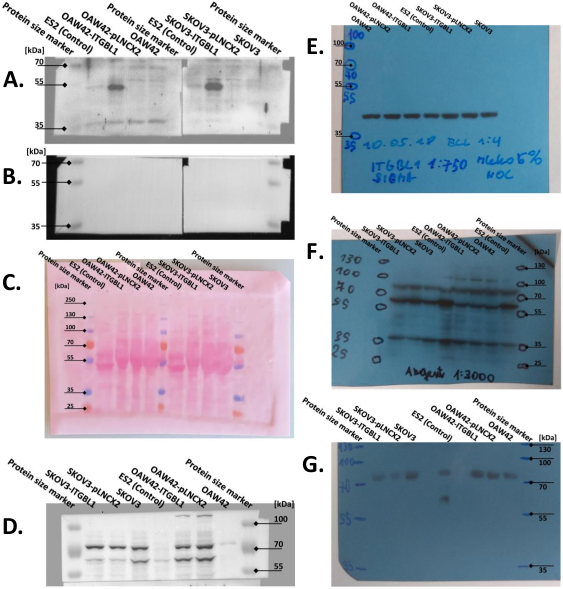

Supplement: Supplementary file 1 [file cancers-12-02676-s001.zip › Suplement ITGBL1 10.09.2020/3. Supplementary Material 3. Western blotting.docx]
