# Supplementary material for: Evaluation of the Role of ITGBL1 in Ovarian Cancer
Source: Cancers (Basel). 2020 Sep 19;12(9):2676. doi: 10.3390/cancers12092676 (PMC7563769; doi:10.3390/cancers12092676)
Supplement: Supplementary file 1 [file cancers-12-02676-s001.zip › Suplement ITGBL1 10.09.2020/4. Supplementary Material 4. Proliferation.docx]

**Supplementary Material 4. Cell proliferation assay and cell-cycle phases distribution**


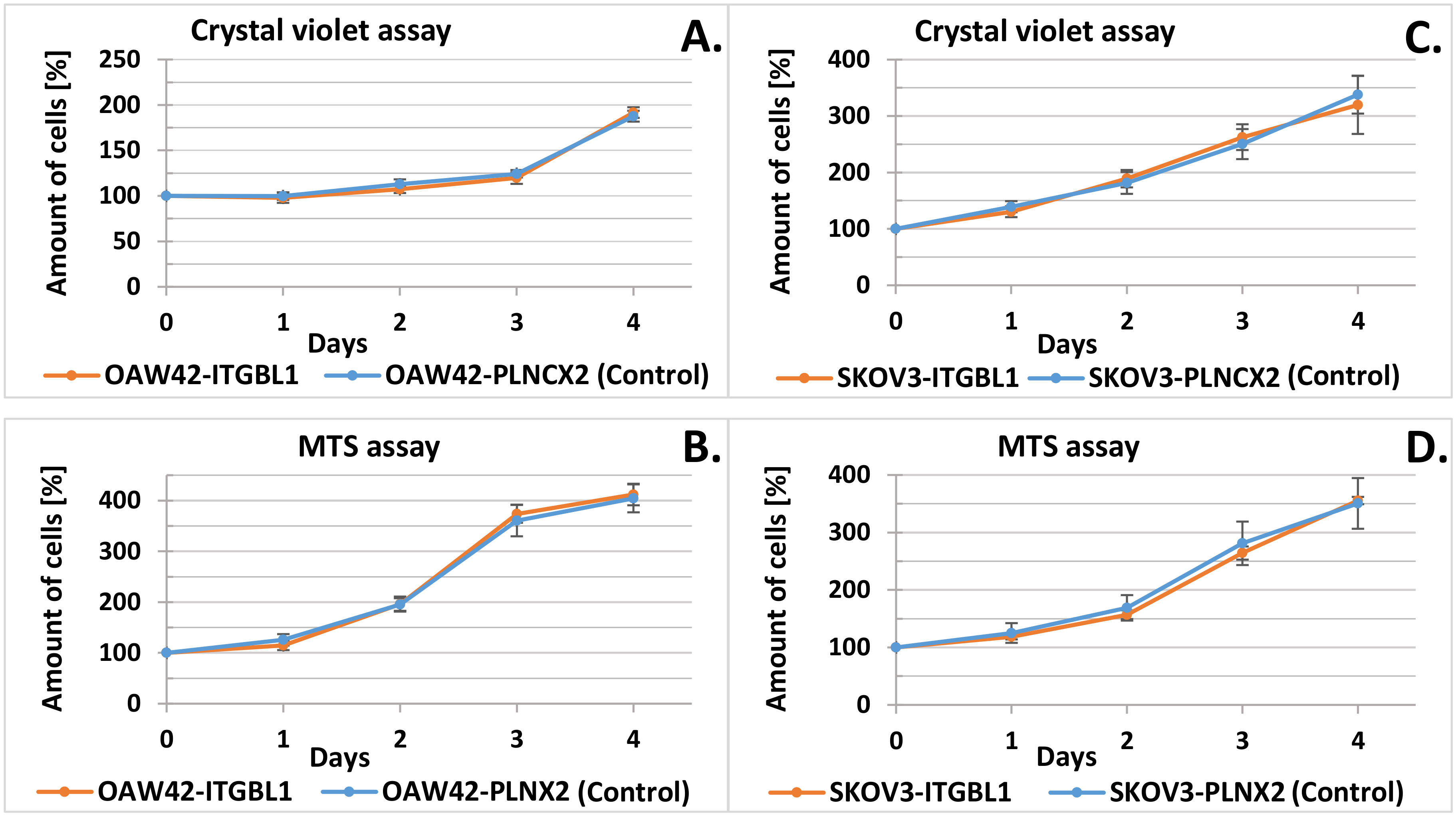


**Supplementary Material 4.1.** **Comparison of proliferation rate between *ITGBL1*-overexpressing and control cells.** (**A**) and (**C**) – Crystal violet assay. (**B**) and (**D**) – MTS assay. Y-axis represents percentage of cells at an indicated time point (mean ± SD, n = 2, each in 18 technical repeats). The absorbance of crystal violet or formazan released from cells at day 0 was assumed as 100%.


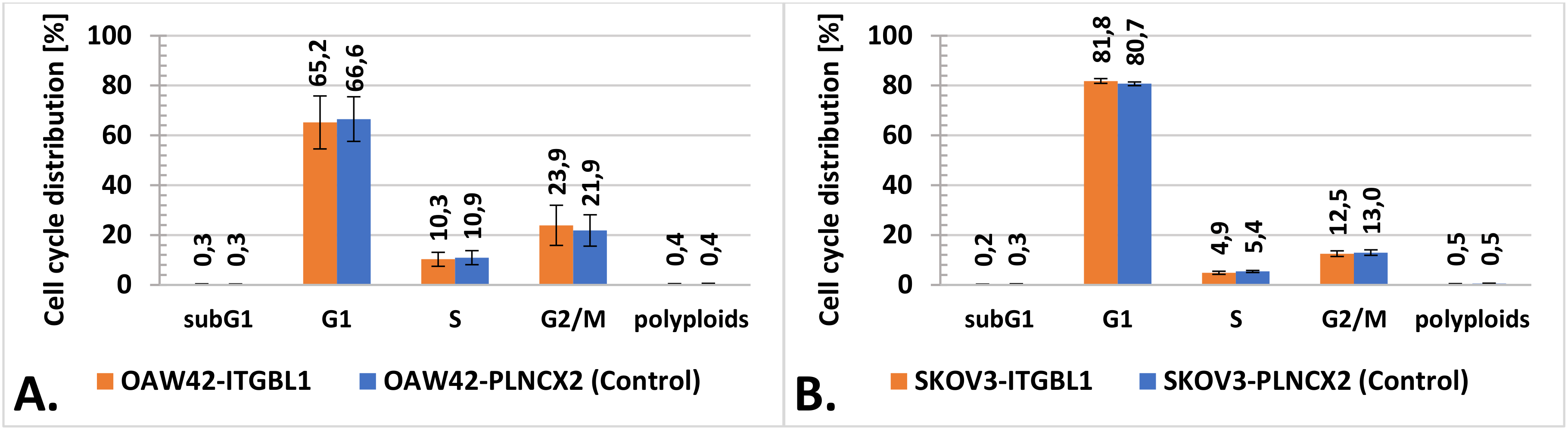


**Supplementary Material 4.2.** **Cell-cycle analysis** **in *ITGBL1*-overexpressing and control cells.** Flow-cytometry analysis using propidium iodide staining. Graphs show distribution of cell cycle phases at 48 hours after seeding (mean ± SD, n = 2, each in 6 technical repeats). (**A**) – OAW42, (**B**) – SKOV3.
