# Supplementary material for: Evaluation of the Role of ITGBL1 in Ovarian Cancer
Source: Cancers (Basel). 2020 Sep 19;12(9):2676. doi: 10.3390/cancers12092676 (PMC7563769; doi:10.3390/cancers12092676)
Supplement: Supplementary file 1 [file cancers-12-02676-s001.zip › Suplement ITGBL1 10.09.2020/5. Supplementary Material 5. PCA.docx]

**Supplementary Material 5. PCA:**

**Principal Component Analysis, hierarchical clustering and signaling pathway analysis in the cell lines with and without ITGBL1 overexpression**

**Supplementary Material 5A. Principal Component Analysis (PCA) performed on all cell lines** (SKOV3 and OAW42; each cell line in a wild-type variant, as well as with an empty pLNCX1, and with ITGBL1 overexpression). The major difference is between cell lines (OAW42 versus SKOV3; PC1, 74% variability); SKOV3-pLNCX2 samples are slight outliers (PC2, 13% variability).

**PCA all cell lines**


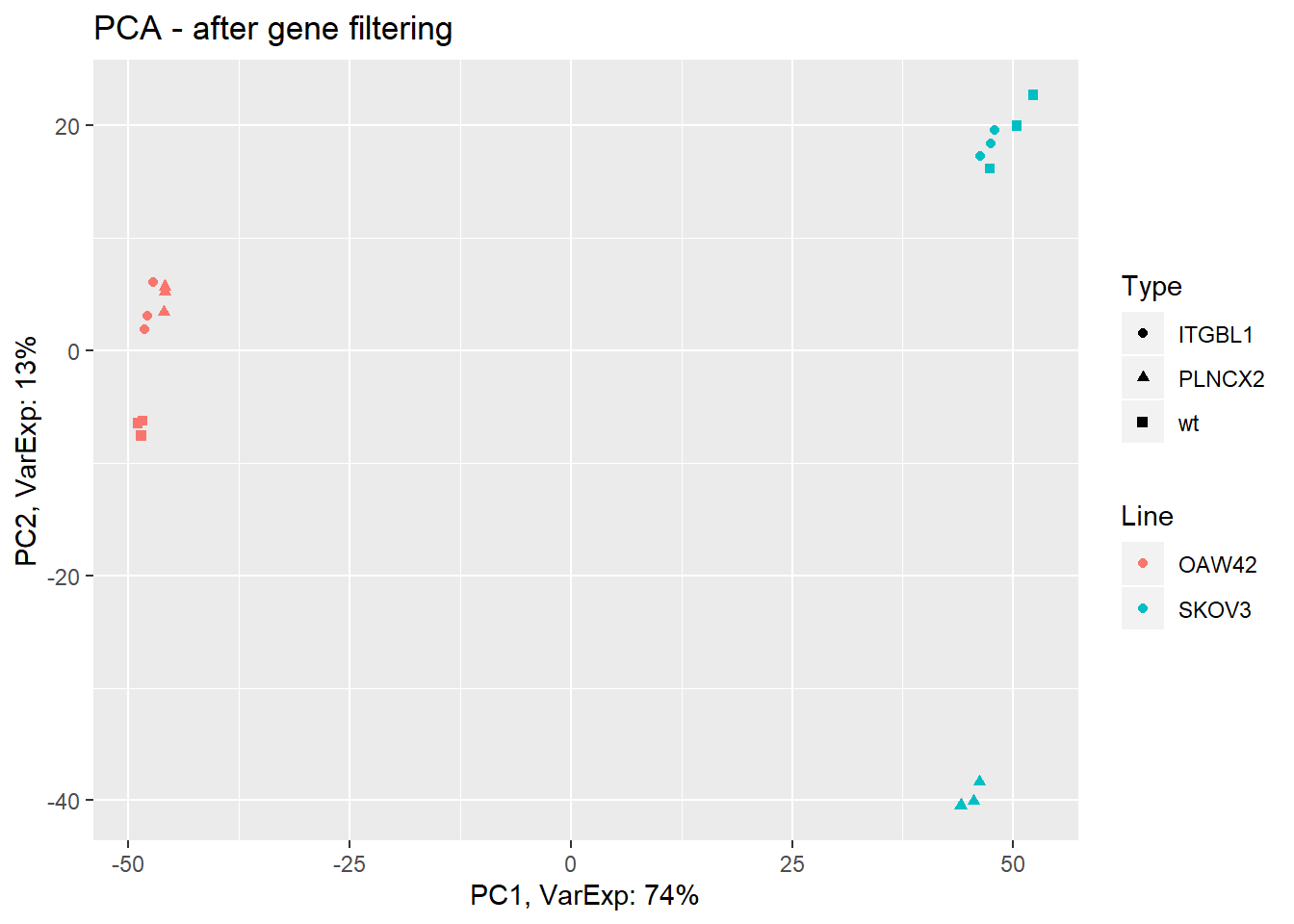


**Supplementary Material 5B. Hierarchical clustering of the samples based on the expression of genes from first and second principal component.** PC1 corresponds to the difference between OAW42 and SKOV3 cell lines. PC2 also distinguishes between cell lines and indicates that OAW42-pLNCX2 cells are different from other OAW42 variants.

**PC1 PC2**


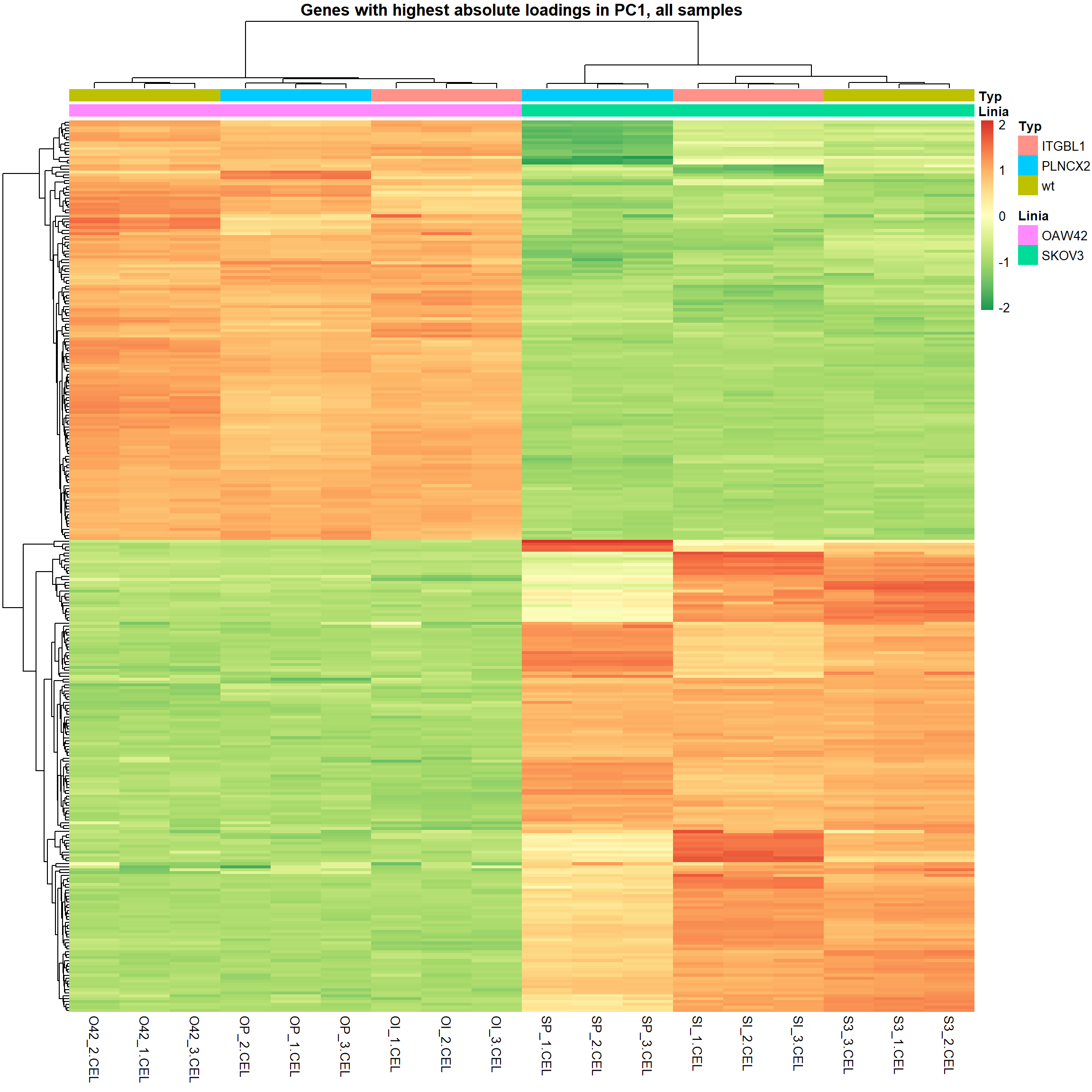

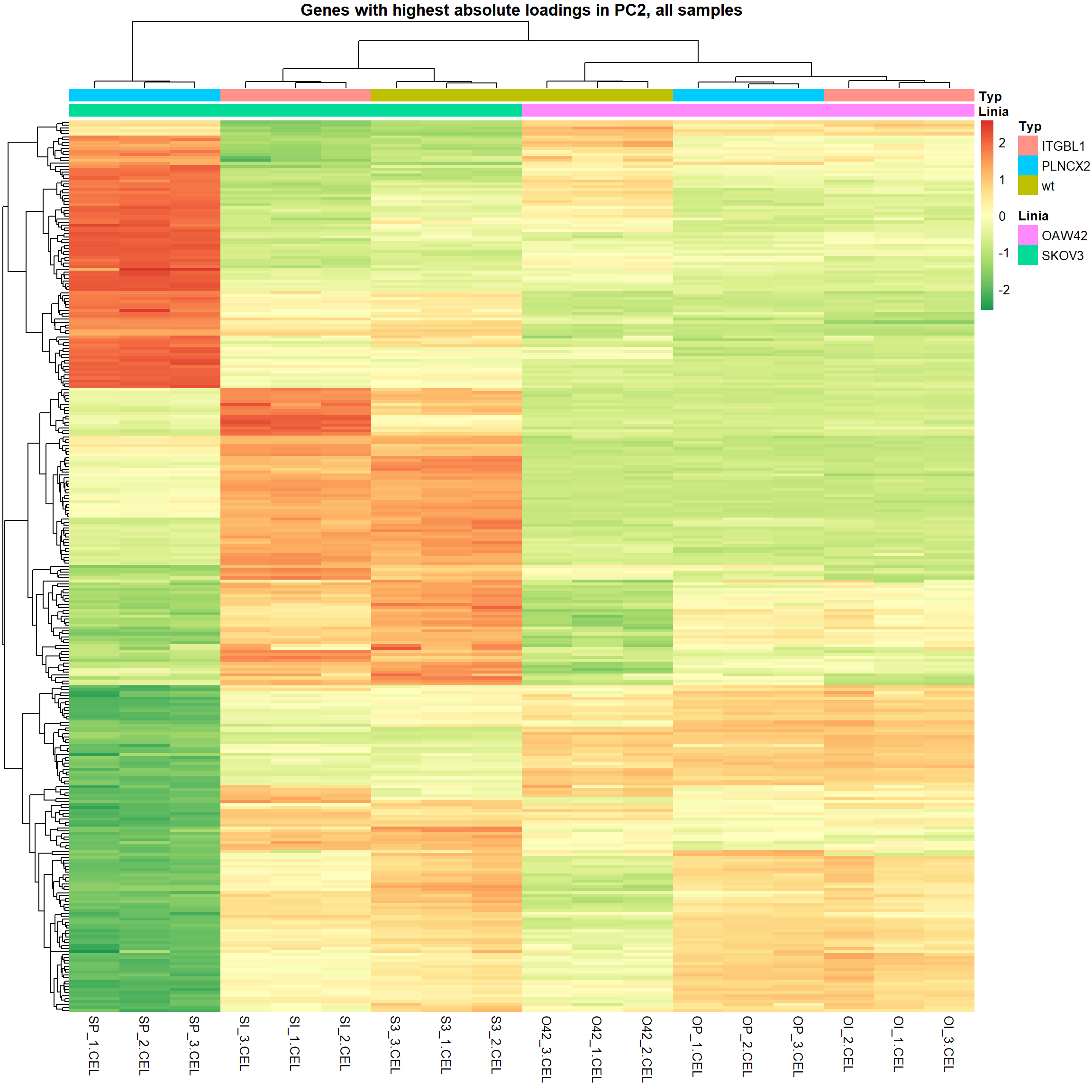


**Supplementary Material 5C. PCA performed on OAW42 cell line variants** (wild-type, with an empty pLNCX1, and with ITGBL1 overexpression).

**PCA OAW42**


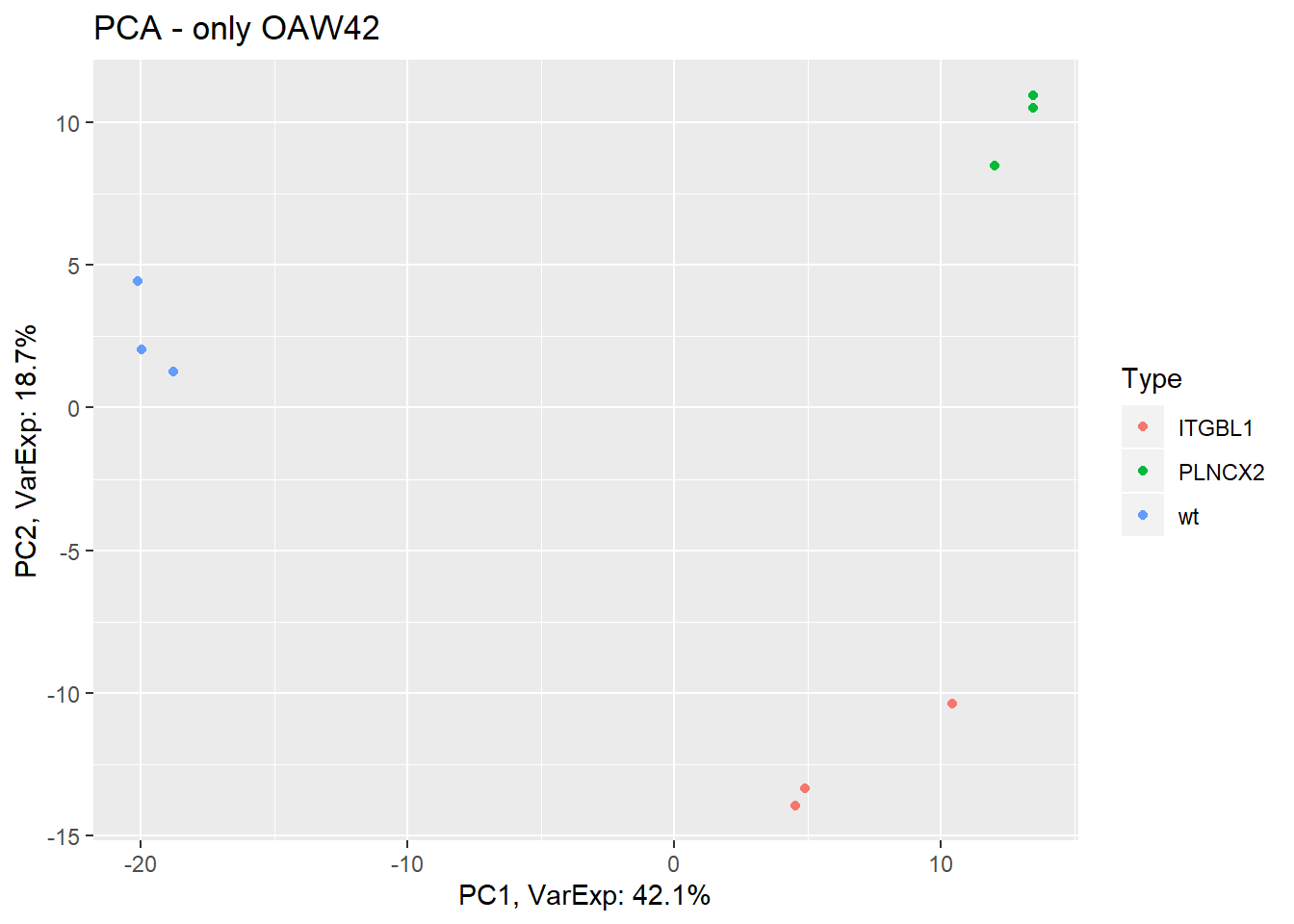


**Supplementary Material 5D. Hierarchical clustering of the samples based on the expression of genes from first and second principal component** (PCA performed on the OAW42 cell line variants: wild-type, with an empty pLNCX2, and with ITGBL1 overexpression). PC1 corresponds to the difference between wt OAW42 and other samples. PC2 differentiates between OAW42 cells with ITGBL1 overexpression and two types of control cells (OAW42-pLNCX1 and wt OAW42).

**PC1 PC2**


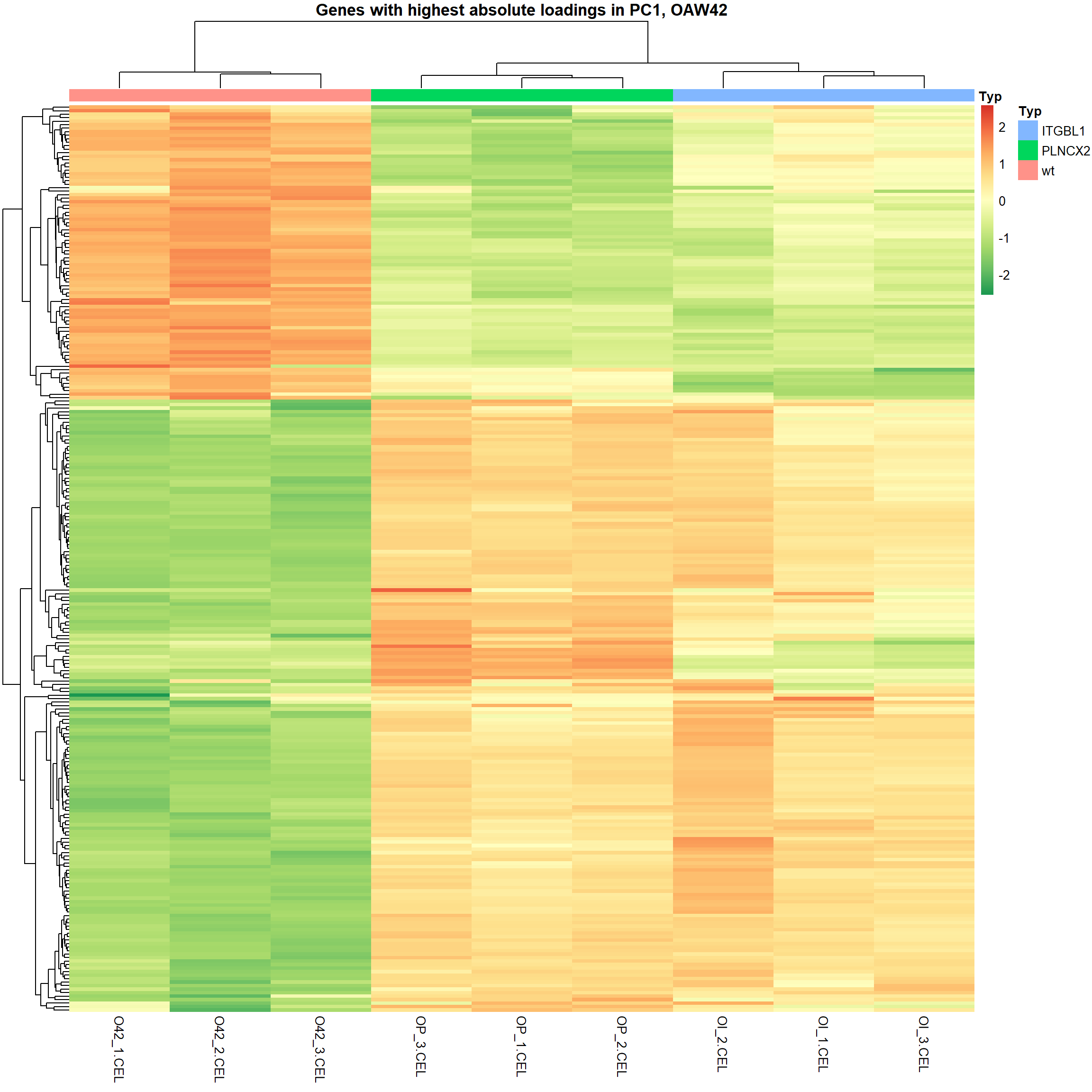


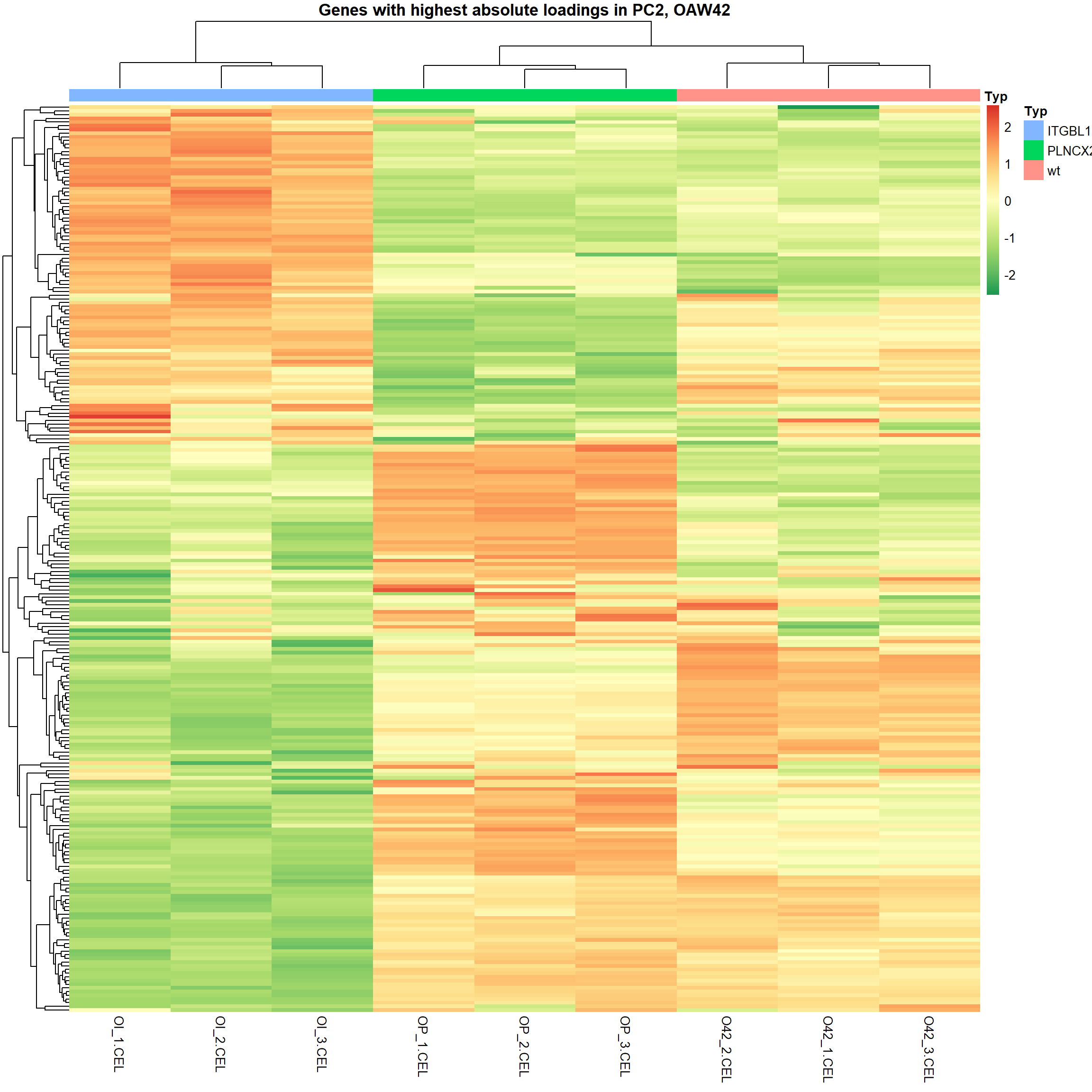


**Supplementary Material 5E. Signaling pathways affected by changed expression of the genes from PC2.** Genes accounting for second principal component (PC2) in the analysis performed on the OAW42 cell line variants (wild-type, with an empty pLNCX2, and with ITGBL1 overexpression) were used for signaling pathways analysis. Seventy two pathways were significantly affected, among them 21 pathways related with extracellular matrix structure and function, integrin signaling, adhesion, motility, etc. (marked in green).

**List of signaling pathways selected with PC2 gene set**

| **No** | **Pathway** | **Gene set length** | **Genes in gene set** | **Genes in gene set** | **p-value** |
| --- | --- | --- | --- | --- | --- |
| 1 | [NABA_MATRISOME](http://software.broadinstitute.org/gsea/msigdb/cards/NABA_MATRISOME.html) | 1026 | 28 | CELA3A, C1QL1, FSTL1, EFEMP1, FN1, HRG, INHBB, LAMA4, MMP7, NELL2, NID1, PI3, SERPINE2, P3H2, SULF2, RELN, SEMA6A, S100A2, S100A4, S100P, CCL20, SEMA3F, SEMA4A, SFRP1, SLIT3, SPP1, TGM2, TNFSF10 | 1,02E-05 |
| 2 | [REACTOME_SYNTHESIS_OF_BILE_ACIDS_AND_BILE_SALTS_VIA_27_HYDROXYCHOLESTEROL](http://software.broadinstitute.org/gsea/msigdb/cards/REACTOME_SYNTHESIS_OF_BILE_ACIDS_AND_BILE_SALTS_VIA_27_HYDROXYCHOLESTEROL.html) | 15 | 4 | AKR1C1, AKR1C2, AKR1C3, NR1H4 | 1,80E-05 |
| 3 | [KEGG_ECM_RECEPTOR_INTERACTION](http://software.broadinstitute.org/gsea/msigdb/cards/KEGG_ECM_RECEPTOR_INTERACTION.html) | 84 | 7 | FN1, ITGA3, ITGB4, LAMA4, RELN, SPP1, CD44 | 4,04E-05 |
| 4 | [PID_INTEGRIN1_PATHWAY](http://software.broadinstitute.org/gsea/msigdb/cards/PID_INTEGRIN1_PATHWAY.html) | 66 | 6 | FN1, ITGA3, LAMA4, NID1, SPP1, TGM2 | 8,91E-05 |
| 5 | [REACTOME_SYNTHESIS_OF_BILE_ACIDS_AND_BILE_SALTS_VIA_7ALPHA_HYDROXYCHOLESTEROL](http://software.broadinstitute.org/gsea/msigdb/cards/REACTOME_SYNTHESIS_OF_BILE_ACIDS_AND_BILE_SALTS_VIA_7ALPHA_HYDROXYCHOLESTEROL.html) | 24 | 4 | AKR1C1, AKR1C2, AKR1C3, NR1H4 | 0,0001 |
| 6 | [PID_INTEGRIN_A9B1_PATHWAY](http://software.broadinstitute.org/gsea/msigdb/cards/PID_INTEGRIN_A9B1_PATHWAY.html) | 25 | 4 | CSF2RA, FN1, SPP1, TGM2 | 0,0002 |
| 7 | [NABA_MATRISOME_ASSOCIATED](http://software.broadinstitute.org/gsea/msigdb/cards/NABA_MATRISOME_ASSOCIATED.html) | 751 | 20 | CELA3A, C1QL1, FSTL1, HRG, INHBB, MMP7, PI3, SERPINE2, P3H2, SULF2, SEMA6A, S100A2, S100A4, S100P, CCL20, SEMA3F, SEMA4A, SFRP1, TGM2, TNFSF10 | 0,0003 |
| 8 | [REACTOME_LAMININ_INTERACTIONS](http://software.broadinstitute.org/gsea/msigdb/cards/REACTOME_LAMININ_INTERACTIONS.html) | 30 | 4 | ITGA3, ITGB4, LAMA4, NID1 | 0,0003 |
| 9 | [REACTOME_SYNTHESIS_OF_BILE_ACIDS_AND_BILE_SALTS_VIA_24_HYDROXYCHOLESTEROL](http://software.broadinstitute.org/gsea/msigdb/cards/REACTOME_SYNTHESIS_OF_BILE_ACIDS_AND_BILE_SALTS_VIA_24_HYDROXYCHOLESTEROL.html) | 14 | 3 | AKR1C1, AKR1C2, AKR1C3 | 0,0004 |
| 10 | [REACTOME_SYNTHESIS_OF_BILE_ACIDS_AND_BILE_SALTS](http://software.broadinstitute.org/gsea/msigdb/cards/REACTOME_SYNTHESIS_OF_BILE_ACIDS_AND_BILE_SALTS.html) | 34 | 4 | AKR1C1, AKR1C2, AKR1C3, NR1H4 | 0,0005 |
| 11 | [REACTOME_BILE_ACID_AND_BILE_SALT_METABOLISM](http://software.broadinstitute.org/gsea/msigdb/cards/REACTOME_BILE_ACID_AND_BILE_SALT_METABOLISM.html) | 43 | 4 | AKR1C1, AKR1C2, AKR1C3, NR1H4 | 0,0013 |
| 12 | [NABA_ECM_GLYCOPROTEINS](http://software.broadinstitute.org/gsea/msigdb/cards/NABA_ECM_GLYCOPROTEINS.html) | 196 | 8 | EFEMP1, FN1, LAMA4, NELL2, NID1, RELN, SLIT3, SPP1 | 0,0016 |
| 13 | [REACTOME_EXTRACELLULAR_MATRIX_ORGANIZATION](http://software.broadinstitute.org/gsea/msigdb/cards/REACTOME_EXTRACELLULAR_MATRIX_ORGANIZATION.html) | 301 | 10 | EFEMP1, FN1, ITGA3, ITGB4, LAMA4, MMP7, NID1, P3H2, SPP1, CD44 | 0,0020 |
| 14 | [KEGG_NICOTINATE_AND_NICOTINAMIDE_METABOLISM](http://software.broadinstitute.org/gsea/msigdb/cards/KEGG_NICOTINATE_AND_NICOTINAMIDE_METABOLISM.html) | 24 | 3 | NNMT, NT5E, ENPP1 | 0,0023 |
| 15 | [KEGG_SMALL_CELL_LUNG_CANCER](http://software.broadinstitute.org/gsea/msigdb/cards/KEGG_SMALL_CELL_LUNG_CANCER.html) | 84 | 5 | FN1, BIRC3, ITGA3, LAMA4, NFKBIA | 0,0024 |
| 16 | [PID_TAP63_PATHWAY](http://software.broadinstitute.org/gsea/msigdb/cards/PID_TAP63_PATHWAY.html) | 54 | 4 | GPX2, ITGA3, ITGB4, S100A2 | 0,0030 |
| 17 | [PID_REELIN_PATHWAY](http://software.broadinstitute.org/gsea/msigdb/cards/PID_REELIN_PATHWAY.html) | 28 | 3 | ITGA3, MAP1B, RELN | 0,0036 |
| 18 | [BIOCARTA_DEATH_PATHWAY](http://software.broadinstitute.org/gsea/msigdb/cards/BIOCARTA_DEATH_PATHWAY.html) | 29 | 3 | BIRC3, NFKBIA, TNFSF10 | 0,0039 |
| 19 | [REACTOME_MET_ACTIVATES_PTK2_SIGNALING](http://software.broadinstitute.org/gsea/msigdb/cards/REACTOME_MET_ACTIVATES_PTK2_SIGNALING.html) | 30 | 3 | FN1, ITGA3, LAMA4 | 0,0043 |
| 20 | [PID_AVB3_OPN_PATHWAY](http://software.broadinstitute.org/gsea/msigdb/cards/PID_AVB3_OPN_PATHWAY.html) | 31 | 3 | NFKBIA, SPP1, CD44 | 0,0048 |
| 21 | [NABA_ECM_REGULATORS](http://software.broadinstitute.org/gsea/msigdb/cards/NABA_ECM_REGULATORS.html) | 238 | 8 | CELA3A, HRG, MMP7, PI3, SERPINE2, P3H2, SULF2, TGM2 | 0,0051 |
| 22 | [BIOCARTA_NPP1_PATHWAY](http://software.broadinstitute.org/gsea/msigdb/cards/BIOCARTA_NPP1_PATHWAY.html) | 10 | 2 | ENPP1, SPP1 | 0,0052 |
| 23 | [REACTOME_REGULATION_OF_COMMISSURAL_AXON_PATHFINDING_BY_SLIT_AND_ROBO](http://software.broadinstitute.org/gsea/msigdb/cards/REACTOME_REGULATION_OF_COMMISSURAL_AXON_PATHFINDING_BY_SLIT_AND_ROBO.html) | 10 | 2 | NELL2, SLIT3 | 0,0052 |
| 24 | [REACTOME_UREA_CYCLE](http://software.broadinstitute.org/gsea/msigdb/cards/REACTOME_UREA_CYCLE.html) | 10 | 2 | SLC25A15, SLC25A2 | 0,0052 |
| 25 | [PID_HIV_NEF_PATHWAY](http://software.broadinstitute.org/gsea/msigdb/cards/PID_HIV_NEF_PATHWAY.html) | 35 | 3 | BIRC3, MAP3K5, NFKBIA | 0,0067 |
| 26 | [KEGG_FOCAL_ADHESION](http://software.broadinstitute.org/gsea/msigdb/cards/KEGG_FOCAL_ADHESION.html) | 199 | 7 | FN1, BIRC3, ITGA3, ITGB4, LAMA4, RELN, SPP1 | 0,0068 |
| 27 | [KEGG_METABOLISM_OF_XENOBIOTICS_BY_CYTOCHROME_P450](http://software.broadinstitute.org/gsea/msigdb/cards/KEGG_METABOLISM_OF_XENOBIOTICS_BY_CYTOCHROME_P450.html) | 70 | 4 | CYP2B6, AKR1C1, AKR1C2, AKR1C3 | 0,0075 |
| 28 | [REACTOME_HSF1_DEPENDENT_TRANSACTIVATION](http://software.broadinstitute.org/gsea/msigdb/cards/REACTOME_HSF1_DEPENDENT_TRANSACTIVATION.html) | 38 | 3 | CRYAB, HSPB8, HSPA1B | 0,0084 |
| 29 | [REACTOME_DISSOLUTION_OF_FIBRIN_CLOT](http://software.broadinstitute.org/gsea/msigdb/cards/REACTOME_DISSOLUTION_OF_FIBRIN_CLOT.html) | 13 | 2 | HRG, SERPINE2 | 0,0087 |
| 30 | [REACTOME_PEPTIDE_HORMONE_BIOSYNTHESIS](http://software.broadinstitute.org/gsea/msigdb/cards/REACTOME_PEPTIDE_HORMONE_BIOSYNTHESIS.html) | 14 | 2 | INHBB, CGB5 | 0,0101 |
| 31 | [REACTOME_MET_PROMOTES_CELL_MOTILITY](http://software.broadinstitute.org/gsea/msigdb/cards/REACTOME_MET_PROMOTES_CELL_MOTILITY.html) | 41 | 3 | FN1, ITGA3, LAMA4 | 0,0104 |
| 32 | [BIOCARTA_CLASSIC_PATHWAY](http://software.broadinstitute.org/gsea/msigdb/cards/BIOCARTA_CLASSIC_PATHWAY.html) | 15 | 2 | C1S, C3 | 0,0116 |
| 33 | [NABA_CORE_MATRISOME](http://software.broadinstitute.org/gsea/msigdb/cards/NABA_CORE_MATRISOME.html) | 275 | 8 | EFEMP1, FN1, LAMA4, NELL2, NID1, RELN, SLIT3, SPP1 | 0,0117 |
| 34 | [PID_INTEGRIN3_PATHWAY](http://software.broadinstitute.org/gsea/msigdb/cards/PID_INTEGRIN3_PATHWAY.html) | 43 | 3 | FN1, LAMA4, SPP1 | 0,0119 |
| 35 | [REACTOME_REGULATION_OF_INSULIN_LIKE_GROWTH_FACTOR_IGF_TRANSPORT_AND_UPTAKE_BY_INSULIN_LIKE_GROWTH_FACTOR_BINDING_PROTEINS_IGFBPS](http://software.broadinstitute.org/gsea/msigdb/cards/REACTOME_REGULATION_OF_INSULIN_LIKE_GROWTH_FACTOR_IGF_TRANSPORT_AND_UPTAKE_BY_INSULIN_LIKE_GROWTH_FACTOR_BINDING_PROTEINS_IGFBPS.html) | 124 | 5 | CDH2, FSTL1, FN1, SPP1, C3 | 0,0123 |
| 36 | [KEGG_PANTOTHENATE_AND_COA_BIOSYNTHESIS](http://software.broadinstitute.org/gsea/msigdb/cards/KEGG_PANTOTHENATE_AND_COA_BIOSYNTHESIS.html) | 16 | 2 | ENPP1, VNN1 | 0,0132 |
| 37 | [PID_TNF_PATHWAY](http://software.broadinstitute.org/gsea/msigdb/cards/PID_TNF_PATHWAY.html) | 46 | 3 | TNIK, BIRC3, MAP3K5 | 0,0142 |
| 38 | [REACTOME_INTEGRIN_CELL_SURFACE_INTERACTIONS](http://software.broadinstitute.org/gsea/msigdb/cards/REACTOME_INTEGRIN_CELL_SURFACE_INTERACTIONS.html) | 85 | 4 | FN1, ITGA3, SPP1, CD44 | 0,0146 |
| 39 | [REACTOME_VITAMIN_B5_PANTOTHENATE_METABOLISM](http://software.broadinstitute.org/gsea/msigdb/cards/REACTOME_VITAMIN_B5_PANTOTHENATE_METABOLISM.html) | 17 | 2 | ENPP1, VNN1 | 0,0148 |
| 40 | [KEGG_APOPTOSIS](http://software.broadinstitute.org/gsea/msigdb/cards/KEGG_APOPTOSIS.html) | 87 | 4 | BIRC3, NFKBIA, TNFSF10, TNFRSF10C | 0,0158 |
| 41 | [BIOCARTA_41BB_PATHWAY](http://software.broadinstitute.org/gsea/msigdb/cards/BIOCARTA_41BB_PATHWAY.html) | 18 | 2 | MAP3K5, NFKBIA | 0,0165 |
| 42 | [REACTOME_OTHER_SEMAPHORIN_INTERACTIONS](http://software.broadinstitute.org/gsea/msigdb/cards/REACTOME_OTHER_SEMAPHORIN_INTERACTIONS.html) | 19 | 2 | SEMA6A, SEMA4A | 0,0184 |
| 43 | [REACTOME_METABOLISM_OF_VITAMINS_AND_COFACTORS](http://software.broadinstitute.org/gsea/msigdb/cards/REACTOME_METABOLISM_OF_VITAMINS_AND_COFACTORS.html) | 189 | 6 | AKR1C1, NNMT, NT5E, ENPP1, AKR1C3, VNN1 | 0,0188 |
| 44 | [REACTOME_DEGRADATION_OF_THE_EXTRACELLULAR_MATRIX](http://software.broadinstitute.org/gsea/msigdb/cards/REACTOME_DEGRADATION_OF_THE_EXTRACELLULAR_MATRIX.html) | 140 | 5 | FN1, MMP7, NID1, SPP1, CD44 | 0,0198 |
| 45 | [BIOCARTA_COMP_PATHWAY](http://software.broadinstitute.org/gsea/msigdb/cards/BIOCARTA_COMP_PATHWAY.html) | 20 | 2 | C1S, C3 | 0,0202 |
| 46 | [REACTOME_RIPK1_MEDIATED_REGULATED_NECROSIS](http://software.broadinstitute.org/gsea/msigdb/cards/REACTOME_RIPK1_MEDIATED_REGULATED_NECROSIS.html) | 20 | 2 | BIRC3, TNFSF10 | 0,0202 |
| 47 | [KEGG_STEROID_HORMONE_BIOSYNTHESIS](http://software.broadinstitute.org/gsea/msigdb/cards/KEGG_STEROID_HORMONE_BIOSYNTHESIS.html) | 55 | 3 | AKR1C1, AKR1C2, AKR1C3 | 0,0229 |
| 48 | [BIOCARTA_HIVNEF_PATHWAY](http://software.broadinstitute.org/gsea/msigdb/cards/BIOCARTA_HIVNEF_PATHWAY.html) | 56 | 3 | BIRC3, MAP3K5, NFKBIA | 0,0240 |
| 49 | [REACTOME_NITRIC_OXIDE_STIMULATES_GUANYLATE_CYCLASE](http://software.broadinstitute.org/gsea/msigdb/cards/REACTOME_NITRIC_OXIDE_STIMULATES_GUANYLATE_CYCLASE.html) | 22 | 2 | PDE10A, NOS1 | 0,0243 |
| 50 | [KEGG_ARACHIDONIC_ACID_METABOLISM](http://software.broadinstitute.org/gsea/msigdb/cards/KEGG_ARACHIDONIC_ACID_METABOLISM.html) | 58 | 3 | CYP2B6, GPX2, AKR1C3 | 0,0263 |
| 51 | [REACTOME_METABOLISM_OF_STEROIDS](http://software.broadinstitute.org/gsea/msigdb/cards/REACTOME_METABOLISM_OF_STEROIDS.html) | 151 | 5 | AKR1C1, AKR1C2, SCD, AKR1C3, NR1H4 | 0,0263 |
| 52 | [REACTOME_ARACHIDONIC_ACID_METABOLISM](http://software.broadinstitute.org/gsea/msigdb/cards/REACTOME_ARACHIDONIC_ACID_METABOLISM.html) | 59 | 3 | PTGR1, GPX2, AKR1C3 | 0,0275 |
| 53 | [REACTOME_NON_INTEGRIN_MEMBRANE_ECM_INTERACTIONS](http://software.broadinstitute.org/gsea/msigdb/cards/REACTOME_NON_INTEGRIN_MEMBRANE_ECM_INTERACTIONS.html) | 59 | 3 | FN1, ITGB4, LAMA4 | 0,0275 |
| 54 | [REACTOME_XENOBIOTICS](http://software.broadinstitute.org/gsea/msigdb/cards/REACTOME_XENOBIOTICS.html) | 24 | 2 | CYP2B6, AHR | 0,0286 |
| 55 | [REACTOME_TRAF6_MEDIATED_NF_KB_ACTIVATION](http://software.broadinstitute.org/gsea/msigdb/cards/REACTOME_TRAF6_MEDIATED_NF_KB_ACTIVATION.html) | 24 | 2 | DDX58, NFKBIA | 0,0286 |
| 56 | [REACTOME_PURINERGIC_SIGNALING_IN_LEISHMANIASIS_INFECTION](http://software.broadinstitute.org/gsea/msigdb/cards/REACTOME_PURINERGIC_SIGNALING_IN_LEISHMANIASIS_INFECTION.html) | 24 | 2 | NT5E, C3 | 0,0286 |
| 57 | [REACTOME_PHASE_I_FUNCTIONALIZATION_OF_COMPOUNDS](http://software.broadinstitute.org/gsea/msigdb/cards/REACTOME_PHASE_I_FUNCTIONALIZATION_OF_COMPOUNDS.html) | 105 | 4 | CYP2B6, AHR, MARC1, NR1H4 | 0,0292 |
| 58 | [REACTOME_TRANSPORT_OF_INORGANIC_CATIONS_ANIONS_AND_AMINO_ACIDS_OLIGOPEPTIDES](http://software.broadinstitute.org/gsea/msigdb/cards/REACTOME_TRANSPORT_OF_INORGANIC_CATIONS_ANIONS_AND_AMINO_ACIDS_OLIGOPEPTIDES.html) | 106 | 4 | SLC38A4, SLC1A1, SLC7A2, SLC12A3 | 0,0301 |
| 59 | [PID_INTEGRIN_CS_PATHWAY](http://software.broadinstitute.org/gsea/msigdb/cards/PID_INTEGRIN_CS_PATHWAY.html) | 26 | 2 | ITGA3, ITGB4 | 0,0332 |
| 60 | [REACTOME_CYTOCHROME_P450_ARRANGED_BY_SUBSTRATE_TYPE](http://software.broadinstitute.org/gsea/msigdb/cards/REACTOME_CYTOCHROME_P450_ARRANGED_BY_SUBSTRATE_TYPE.html) | 65 | 3 | CYP2B6, AHR, NR1H4 | 0,0352 |
| 61 | [REACTOME_ENDOGENOUS_STEROLS](http://software.broadinstitute.org/gsea/msigdb/cards/REACTOME_ENDOGENOUS_STEROLS.html) | 27 | 2 | AHR, NR1H4 | 0,0356 |
| 62 | [REACTOME_SYNDECAN_INTERACTIONS](http://software.broadinstitute.org/gsea/msigdb/cards/REACTOME_SYNDECAN_INTERACTIONS.html) | 27 | 2 | FN1, ITGB4 | 0,0356 |
| 63 | [REACTOME_BIOLOGICAL_OXIDATIONS](http://software.broadinstitute.org/gsea/msigdb/cards/REACTOME_BIOLOGICAL_OXIDATIONS.html) | 221 | 6 | CYP2B6, AHR, NNMT, MARC1, PAPSS2, NR1H4 | 0,0365 |
| 64 | [NABA_SECRETED_FACTORS](http://software.broadinstitute.org/gsea/msigdb/cards/NABA_SECRETED_FACTORS.html) | 343 | 8 | FSTL1, INHBB, S100A2, S100A4, S100P, CCL20, SFRP1, TNFSF10 | 0,0374 |
| 65 | [PID_TRAIL_PATHWAY](http://software.broadinstitute.org/gsea/msigdb/cards/PID_TRAIL_PATHWAY.html) | 28 | 2 | TNFSF10, TNFRSF10C | 0,0380 |
| 66 | [PID_LIS1_PATHWAY](http://software.broadinstitute.org/gsea/msigdb/cards/PID_LIS1_PATHWAY.html) | 28 | 2 | MAP1B, RELN | 0,0380 |
| 67 | [REACTOME_SELENOAMINO_ACID_METABOLISM](http://software.broadinstitute.org/gsea/msigdb/cards/REACTOME_SELENOAMINO_ACID_METABOLISM.html) | 118 | 4 | NNMT, RPS7, RPS10, PAPSS2 | 0,0420 |
| 68 | [REACTOME_SURFACTANT_METABOLISM](http://software.broadinstitute.org/gsea/msigdb/cards/REACTOME_SURFACTANT_METABOLISM.html) | 30 | 2 | CSF2RA, ZDHHC2 | 0,0431 |
| 69 | [PID_CD40_PATHWAY](http://software.broadinstitute.org/gsea/msigdb/cards/PID_CD40_PATHWAY.html) | 31 | 2 | BIRC3, NFKBIA | 0,0458 |
| 70 | [REACTOME_NICOTINATE_METABOLISM](http://software.broadinstitute.org/gsea/msigdb/cards/REACTOME_NICOTINATE_METABOLISM.html) | 31 | 2 | NNMT, NT5E | 0,0458 |
| 71 | [REACTOME_METABOLISM_OF_WATER_SOLUBLE_VITAMINS_AND_COFACTORS](http://software.broadinstitute.org/gsea/msigdb/cards/REACTOME_METABOLISM_OF_WATER_SOLUBLE_VITAMINS_AND_COFACTORS.html) | 123 | 4 | NNMT, NT5E, ENPP1, VNN1 | 0,0477 |
| 72 | [KEGG_ARRHYTHMOGENIC_RIGHT_VENTRICULAR_CARDIOMYOPATHY_ARVC](http://software.broadinstitute.org/gsea/msigdb/cards/KEGG_ARRHYTHMOGENIC_RIGHT_VENTRICULAR_CARDIOMYOPATHY_ARVC.html) | 74 | 3 | CDH2, ITGA3, ITGB4 | 0,0487 |

**Supplementary Material 5F. PCA performed on SKOV3 cell line variants** (wild-type, with an empty pLNCX1, and with ITGBL1 overexpression).

**PCA SKOV3**


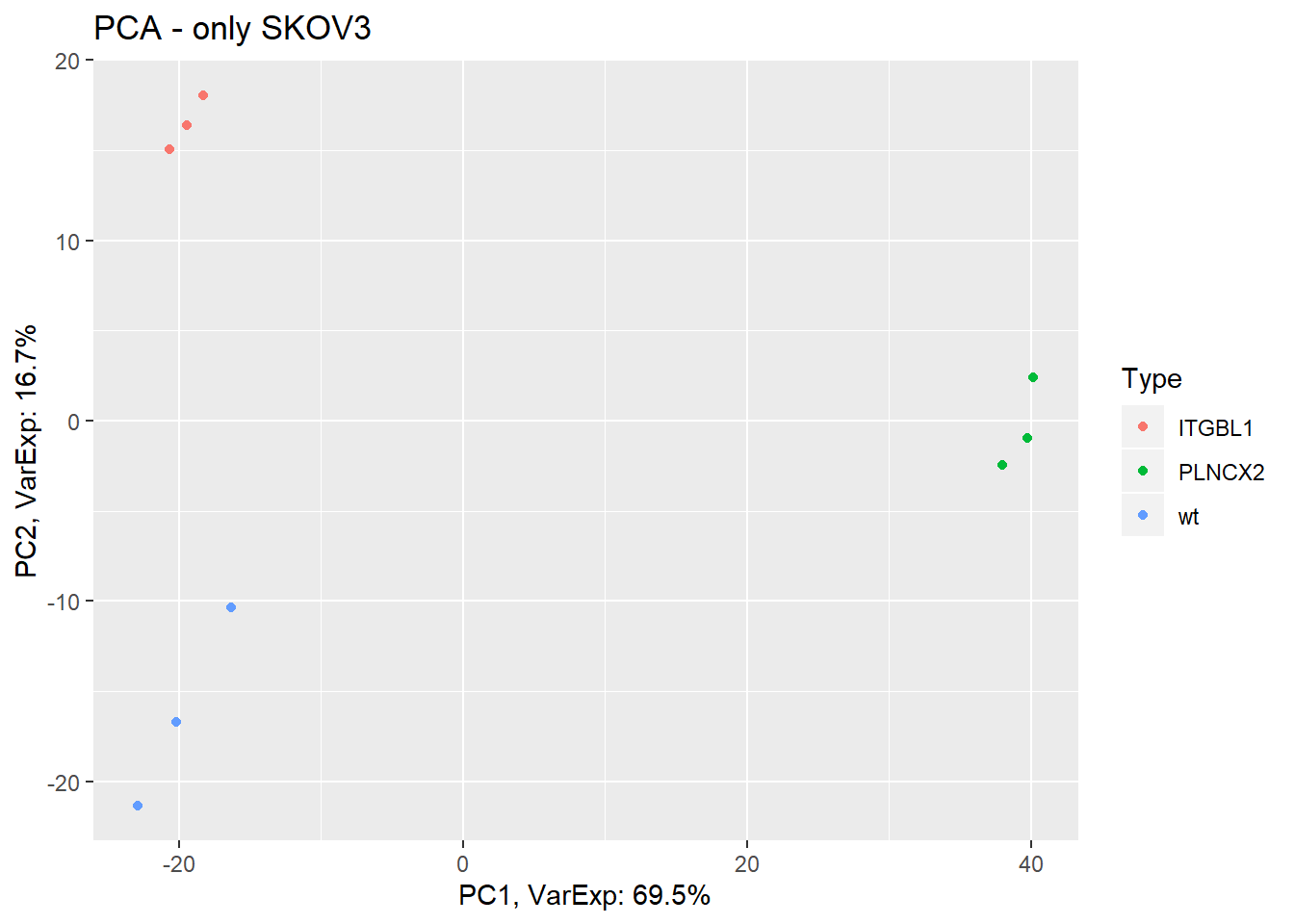


**Supplementary Material 5G. Hierarchical clustering of the samples based on the expression of genes from first and second principal component** (PCA performed on the SKOV3 cell line variants: wild-type, with an empty pLNCX2, and with ITGBL1 overexpression). PC1 corresponds to the difference between SKOV3-pLNCX2 and other samples. PC2 genes expression resulted in clustering control samples (OAW42-pLNCX1 and wt SKOV3) together and SKOV3-ITGBL1 samples apart.

**PC1 PC2**


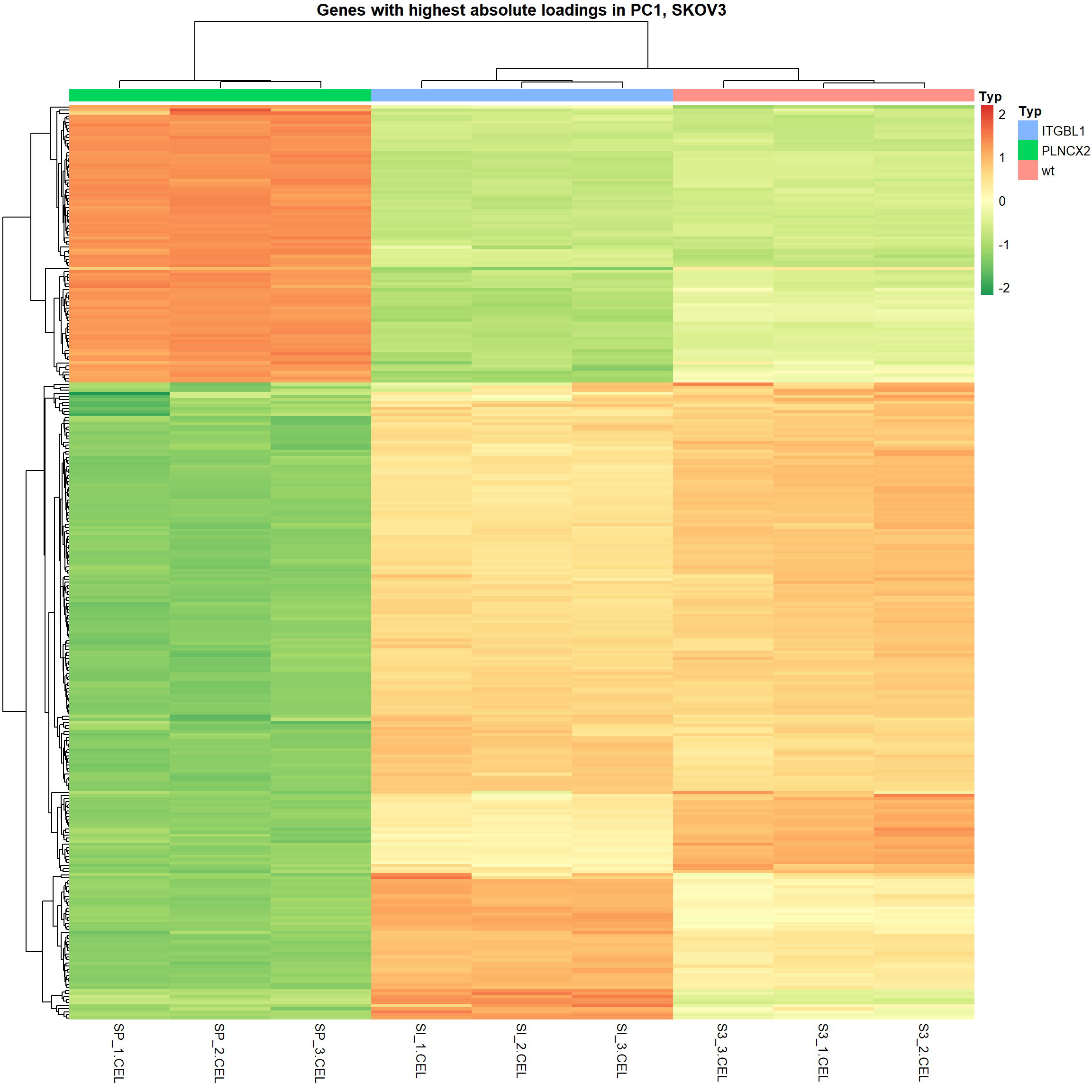

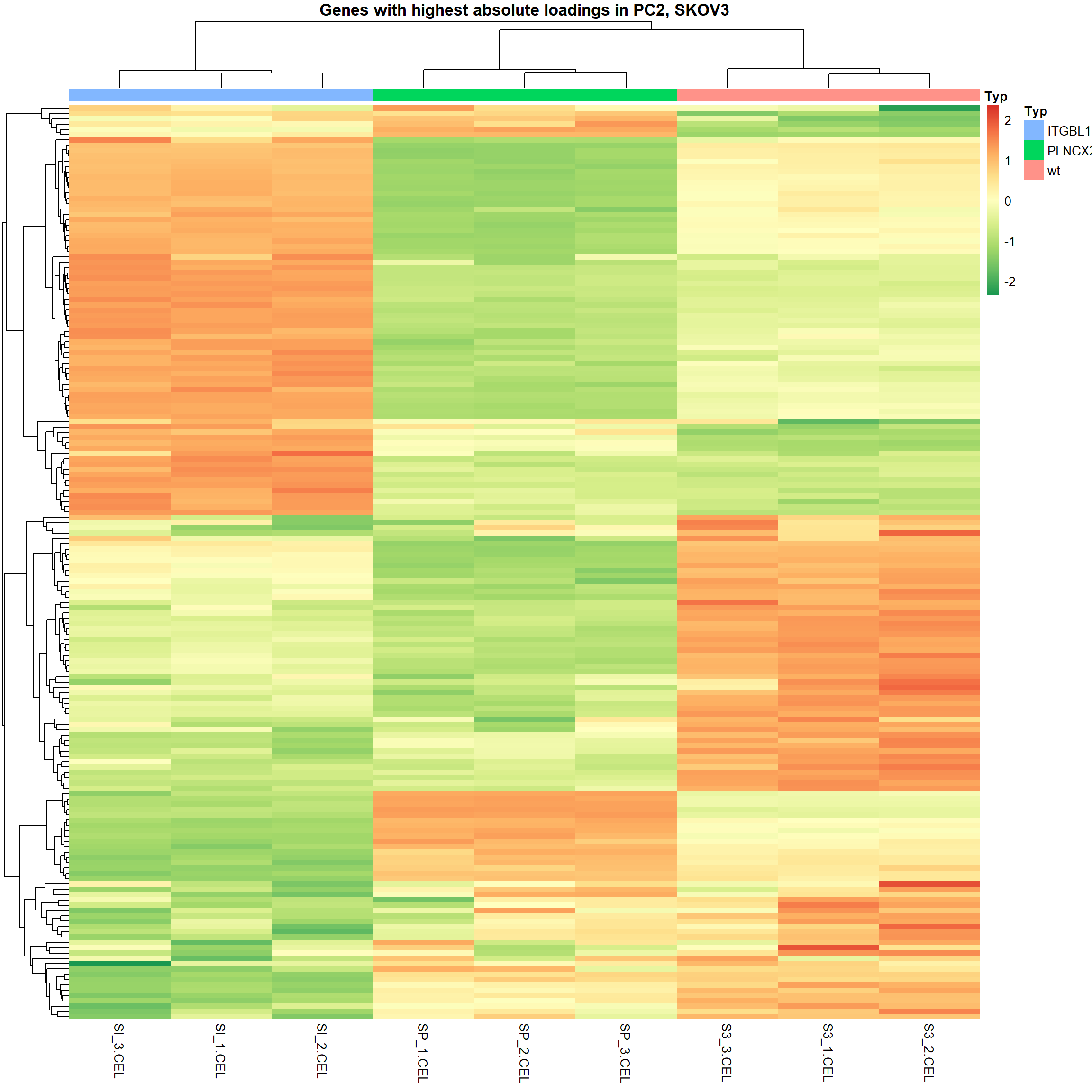


**Supplementary Material 5H. Signaling pathways affected by changed expression of the genes from PC2.** Genes accounting for second principal component (PC2) in the analysis performed on the SKOV3 cell line variants (wild-type, with an empty pLNCX2, and with ITGBL1 overexpression) were used for signaling pathways analysis. Among 44 significantly affected pathways, 18 were related with cellular communication, extracellular matrix structure and function, integrin signaling, etc. (marked in green).

**List of signaling pathways selected with PC2 gene set**

| **No** | **Pathway** | **Gene set length** | **Genes in gene set** | **Genes in gene set** | **p-value** |
| --- | --- | --- | --- | --- | --- |
| 1 | [REACTOME_CELL_JUNCTION_ORGANIZATION](http://software.broadinstitute.org/gsea/msigdb/cards/REACTOME_CELL_JUNCTION_ORGANIZATION.html) | 92 | 6 | CDH6, CLDN16, CADM1, LAMC2, CLDN10, CLDN1 | 8,00E-05 |
| 2 | [REACTOME_CELL_CELL_JUNCTION_ORGANIZATION](http://software.broadinstitute.org/gsea/msigdb/cards/REACTOME_CELL_CELL_JUNCTION_ORGANIZATION.html) | 65 | 5 | CDH6, CLDN16, CADM1, CLDN10, CLDN1 | 0,0001 |
| 3 | [REACTOME_CELL_CELL_COMMUNICATION](http://software.broadinstitute.org/gsea/msigdb/cards/REACTOME_CELL_CELL_COMMUNICATION.html) | 130 | 6 | CDH6, CLDN16, CADM1, LAMC2, CLDN10, CLDN1 | 0,0005 |
| 4 | [REACTOME_TIGHT_JUNCTION_INTERACTIONS](http://software.broadinstitute.org/gsea/msigdb/cards/REACTOME_TIGHT_JUNCTION_INTERACTIONS.html) | 30 | 3 | CLDN16, CLDN10, CLDN1 | 0,0016 |
| 5 | [REACTOME_OAS_ANTIVIRAL_RESPONSE](http://software.broadinstitute.org/gsea/msigdb/cards/REACTOME_OAS_ANTIVIRAL_RESPONSE.html) | 9 | 2 | OAS2, OAS3 | 0,0021 |
| 6 | [REACTOME_INTERFERON_ALPHA_BETA_SIGNALING](http://software.broadinstitute.org/gsea/msigdb/cards/REACTOME_INTERFERON_ALPHA_BETA_SIGNALING.html) | 70 | 4 | EGR1, IFI27, OAS2, OAS3 | 0,0021 |
| 7 | REACTOME_SLC_MEDIATED_TRANSMEMBRANE_TRANSPORT | 250 | 7 | SLC34A2, SLCO2B1, CP, SLC26A2, SLC44A2, SLC12A3, SLC16A2 | 0,0034 |
| 8 | [REACTOME_TRANSPORT_OF_ORGANIC_ANIONS](http://software.broadinstitute.org/gsea/msigdb/cards/REACTOME_TRANSPORT_OF_ORGANIC_ANIONS.html) | 12 | 2 | SLCO2B1, SLC16A2 | 0,0037 |
| 9 | [PID_SYNDECAN_1_PATHWAY](http://software.broadinstitute.org/gsea/msigdb/cards/PID_SYNDECAN_1_PATHWAY.html) | 46 | 3 | COL8A1, MMP1, MMP7 | 0,0054 |
| 10 | [REACTOME_SLC_TRANSPORTER_DISORDERS](http://software.broadinstitute.org/gsea/msigdb/cards/REACTOME_SLC_TRANSPORTER_DISORDERS.html) | 99 | 4 | SLC34A2, CP, SLC26A2, SLC12A3 | 0,0073 |
| 11 | REACTOME_INTERLEUKIN_4_AND_INTERLEUKIN_13_SIGNALING | 111 | 4 | S1PR1, IL1A, MMP1, SAA1 | 0,0108 |
| 12 | [BIOCARTA_NFKB_PATHWAY](http://software.broadinstitute.org/gsea/msigdb/cards/BIOCARTA_NFKB_PATHWAY.html) | 21 | 2 | IL1A, TNFAIP3 | 0,0113 |
| 13 | REACTOME_ASSEMBLY_OF_COLLAGEN_FIBRILS_AND_OTHER_MULTIMERIC_STRUCTURES | 61 | 3 | COL8A1, LAMC2, MMP7 | 0,0117 |
| 14 | REACTOME_COLLAGEN_DEGRADATION | 64 | 3 | COL8A1, MMP1, MMP7 | 0,0134 |
| 15 | [REACTOME_TRAF6_MEDIATED_NF_KB_ACTIVATION](http://software.broadinstitute.org/gsea/msigdb/cards/REACTOME_TRAF6_MEDIATED_NF_KB_ACTIVATION.html) | 24 | 2 | SAA1, IFIH1 | 0,0146 |
| 16 | [REACTOME_REGULATION_OF_INSULIN_LIKE_GROWTH_FACTOR_IGF_TRANSPORT_AND_UPTAKE_BY_INSULIN_LIKE_GROWTH_FACTOR_BINDING_PROTEINS_IGFBPS](http://software.broadinstitute.org/gsea/msigdb/cards/REACTOME_REGULATION_OF_INSULIN_LIKE_GROWTH_FACTOR_IGF_TRANSPORT_AND_UPTAKE_BY_INSULIN_LIKE_GROWTH_FACTOR_BINDING_PROTEINS_IGFBPS.html) | 124 | 4 | FSTL1, CP, AMTN, MMP1 | 0,0157 |
| 17 | KEGG_CELL_ADHESION_MOLECULES_CAMS | 133 | 4 | CLDN16, CADM1, CLDN10, CLDN1 | 0,0198 |
| 18 | NABA_CORE_MATRISOME | 275 | 6 | EDIL3, COL8A1, LAMC2, SRGN, SPARC, FNDC1 | 0,0202 |
| 19 | [REACTOME_INTERFERON_SIGNALING](http://software.broadinstitute.org/gsea/msigdb/cards/REACTOME_INTERFERON_SIGNALING.html) | 202 | 5 | TRIM6, EGR1, IFI27, OAS2, OAS3 | 0,0205 |
| 20 | [REACTOME_DDX58_IFIH1_MEDIATED_INDUCTION_OF_INTERFERON_ALPHA_BETA](http://software.broadinstitute.org/gsea/msigdb/cards/REACTOME_DDX58_IFIH1_MEDIATED_INDUCTION_OF_INTERFERON_ALPHA_BETA.html) | 78 | 3 | SAA1, IFIH1, TNFAIP3 | 0,0226 |
| 21 | REACTOME_DEGRADATION_OF_THE_EXTRACELLULAR_MATRIX | 140 | 4 | COL8A1, LAMC2, MMP1, MMP7 | 0,0234 |
| 22 | REACTOME_ADHERENS_JUNCTIONS_INTERACTIONS | 33 | 2 | CDH6, CADM1 | 0,0268 |
| 23 | [REACTOME_ACTIVATION_OF_MATRIX_METALLOPROTEINASES](http://software.broadinstitute.org/gsea/msigdb/cards/REACTOME_ACTIVATION_OF_MATRIX_METALLOPROTEINASES.html) | 33 | 2 | MMP1, MMP7 | 0,0268 |
| 24 | [NABA_MATRISOME](http://software.broadinstitute.org/gsea/msigdb/cards/NABA_MATRISOME.html) | 1026 | 14 | GPC6, EDIL3, FSTL1, COL8A1, IL1A, C1QTNF9B, LAMC2, MMP1, MMP7, SRGN, CCL20, SFRP1, SPARC, FNDC1 | 0,0281 |
| 25 | [REACTOME_NEGATIVE_REGULATORS_OF_DDX58_IFIH1_SIGNALING](http://software.broadinstitute.org/gsea/msigdb/cards/REACTOME_NEGATIVE_REGULATORS_OF_DDX58_IFIH1_SIGNALING.html) | 34 | 2 | IFIH1, TNFAIP3 | 0,0283 |
| 26 | [KEGG_PRION_DISEASES](http://software.broadinstitute.org/gsea/msigdb/cards/KEGG_PRION_DISEASES.html) | 35 | 2 | EGR1, IL1A | 0,0299 |
| 27 | REACTOME_COLLAGEN_FORMATION | 90 | 3 | COL8A1, LAMC2, MMP7 | 0,0326 |
| 28 | [REACTOME_CYTOKINE_SIGNALING_IN_IMMUNE_SYSTEM](http://software.broadinstitute.org/gsea/msigdb/cards/REACTOME_CYTOKINE_SIGNALING_IN_IMMUNE_SYSTEM.html) | 858 | 12 | TRIM6, S1PR1, EGR1, FGFR2, GFRA1, IFI27, IL1A, MMP1, OAS2, OAS3, SAA1, CCL20 | 0,0346 |
| 29 | [REACTOME_OVARIAN_TUMOR_DOMAIN_PROTEASES](http://software.broadinstitute.org/gsea/msigdb/cards/REACTOME_OVARIAN_TUMOR_DOMAIN_PROTEASES.html) | 38 | 2 | IFIH1, TNFAIP3 | 0,0348 |
| 30 | [REACTOME_INTERFERON_GAMMA_SIGNALING](http://software.broadinstitute.org/gsea/msigdb/cards/REACTOME_INTERFERON_GAMMA_SIGNALING.html) | 93 | 3 | TRIM6, OAS2, OAS3 | 0,0354 |
| 31 | [REACTOME_NGF_STIMULATED_TRANSCRIPTION](http://software.broadinstitute.org/gsea/msigdb/cards/REACTOME_NGF_STIMULATED_TRANSCRIPTION.html) | 39 | 2 | EGR1, F3 | 0,0364 |
| 32 | [REACTOME_ACTIVATION_OF_NOXA_AND_TRANSLOCATION_TO_MITOCHONDRIA](http://software.broadinstitute.org/gsea/msigdb/cards/REACTOME_ACTIVATION_OF_NOXA_AND_TRANSLOCATION_TO_MITOCHONDRIA.html) | 5 | 1 | PMAIP1 | 0,0380 |
| 33 | [REACTOME_EXTRINSIC_PATHWAY_OF_FIBRIN_CLOT_FORMATION](http://software.broadinstitute.org/gsea/msigdb/cards/REACTOME_EXTRINSIC_PATHWAY_OF_FIBRIN_CLOT_FORMATION.html) | 5 | 1 | F3 | 0,0380 |
| 34 | [REACTOME_SODIUM_COUPLED_PHOSPHATE_COTRANSPORTERS](http://software.broadinstitute.org/gsea/msigdb/cards/REACTOME_SODIUM_COUPLED_PHOSPHATE_COTRANSPORTERS.html) | 5 | 1 | SLC34A2 | 0,0380 |
| 35 | [REACTOME_G2_PHASE](http://software.broadinstitute.org/gsea/msigdb/cards/REACTOME_G2_PHASE.html) | 5 | 1 | CCNA1 | 0,0380 |
| 36 | [REACTOME_TYROSINE_CATABOLISM](http://software.broadinstitute.org/gsea/msigdb/cards/REACTOME_TYROSINE_CATABOLISM.html) | 5 | 1 | HGD | 0,0380 |
| 37 | [REACTOME_DISEASES_ASSOCIATED_WITH_GLYCOSAMINOGLYCAN_METABOLISM](http://software.broadinstitute.org/gsea/msigdb/cards/REACTOME_DISEASES_ASSOCIATED_WITH_GLYCOSAMINOGLYCAN_METABOLISM.html) | 41 | 2 | GPC6, SLC26A2 | 0,0399 |
| 38 | [REACTOME_TRANSPORT_OF_VITAMINS_NUCLEOSIDES_AND_RELATED_MOLECULES](http://software.broadinstitute.org/gsea/msigdb/cards/REACTOME_TRANSPORT_OF_VITAMINS_NUCLEOSIDES_AND_RELATED_MOLECULES.html) | 44 | 2 | SLCO2B1, SLC16A2 | 0,0454 |
| 39 | [REACTOME_TRANSPORT_AND_SYNTHESIS_OF_PAPS](http://software.broadinstitute.org/gsea/msigdb/cards/REACTOME_TRANSPORT_AND_SYNTHESIS_OF_PAPS.html) | 6 | 1 | SLC26A2 | 0,0454 |
| 40 | [REACTOME_SCAVENGING_BY_CLASS_B_RECEPTORS](http://software.broadinstitute.org/gsea/msigdb/cards/REACTOME_SCAVENGING_BY_CLASS_B_RECEPTORS.html) | 6 | 1 | SAA1 | 0,0454 |
| 41 | [REACTOME_DISORDERS_OF_TRANSMEMBRANE_TRANSPORTERS](http://software.broadinstitute.org/gsea/msigdb/cards/REACTOME_DISORDERS_OF_TRANSMEMBRANE_TRANSPORTERS.html) | 176 | 4 | SLC34A2, CP, SLC26A2, SLC12A3 | 0,0478 |
| 42 | [REACTOME_TRANSPORT_OF_INORGANIC_CATIONS_ANIONS_AND_AMINO_ACIDS_OLIGOPEPTIDES](http://software.broadinstitute.org/gsea/msigdb/cards/REACTOME_TRANSPORT_OF_INORGANIC_CATIONS_ANIONS_AND_AMINO_ACIDS_OLIGOPEPTIDES.html) | 106 | 3 | SLC34A2, SLC26A2, SLC12A3 | 0,0490 |
| 43 | PID_A6B1_A6B4_INTEGRIN_PATHWAY | 46 | 2 | IL1A, LAMC2 | 0,0492 |
| 44 | REACTOME_INTERLEUKIN_10_SIGNALING | 46 | 2 | IL1A, CCL20 | 0,0492 |

**Supplementary Material 5I. PCA performed on OAW42 cell line variants with an empty pLNCX2 (control) and with ITGBL1 overexpression.**

**PCA OAW42-PLNCX2 vs OAW42-ITGBL1**


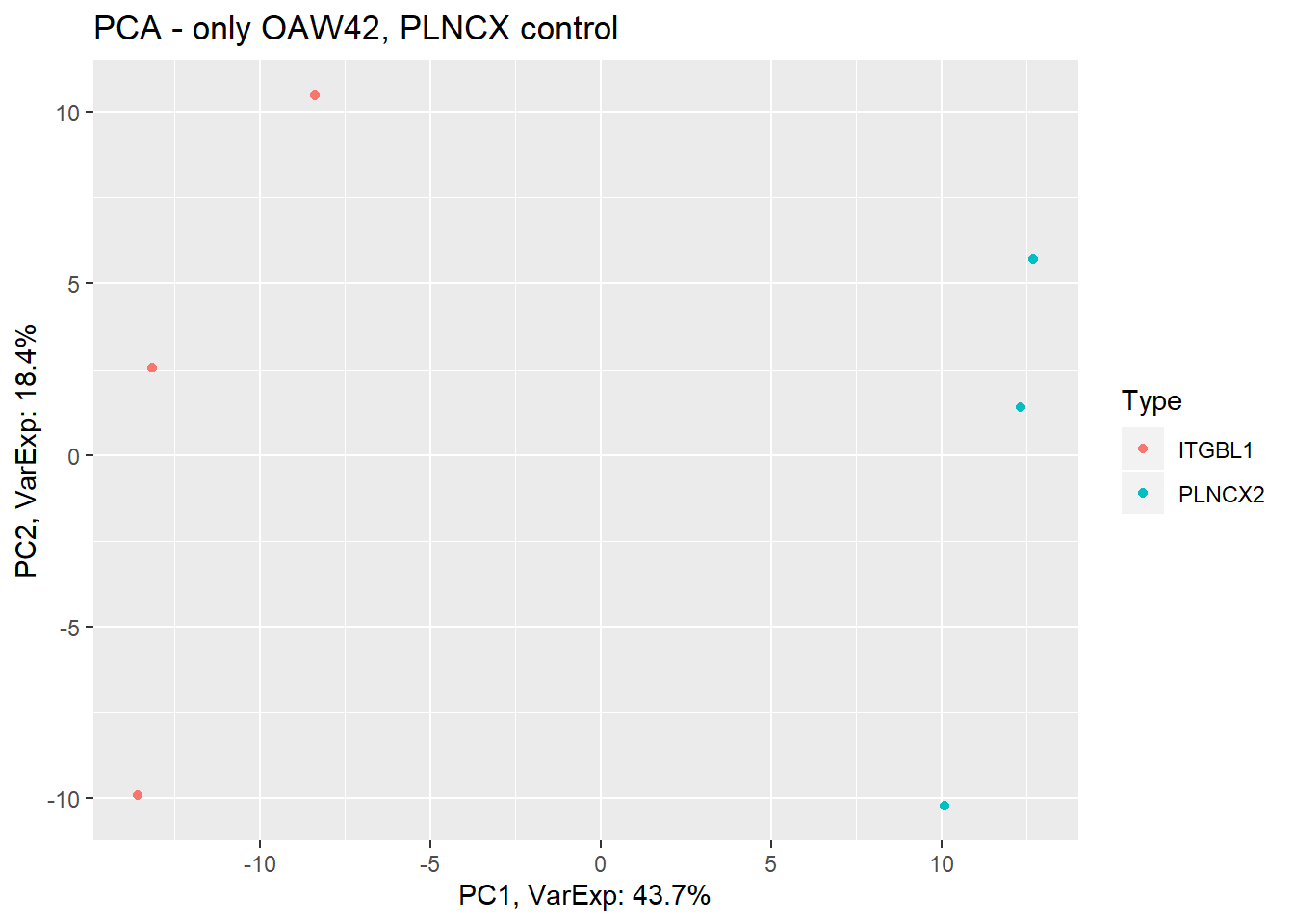


**Supplementary Material 5J. Hierarchical clustering of the samples based on the expression of genes from first principal component** (PCA performed on the OAW42 cell line with an empty pLNCX1, and with ITGBL1 overexpression). PC1 differentiates between OAW42 cells with overexpression of ITGBL1 and control OAW42-pLNCX1.

**PC1**


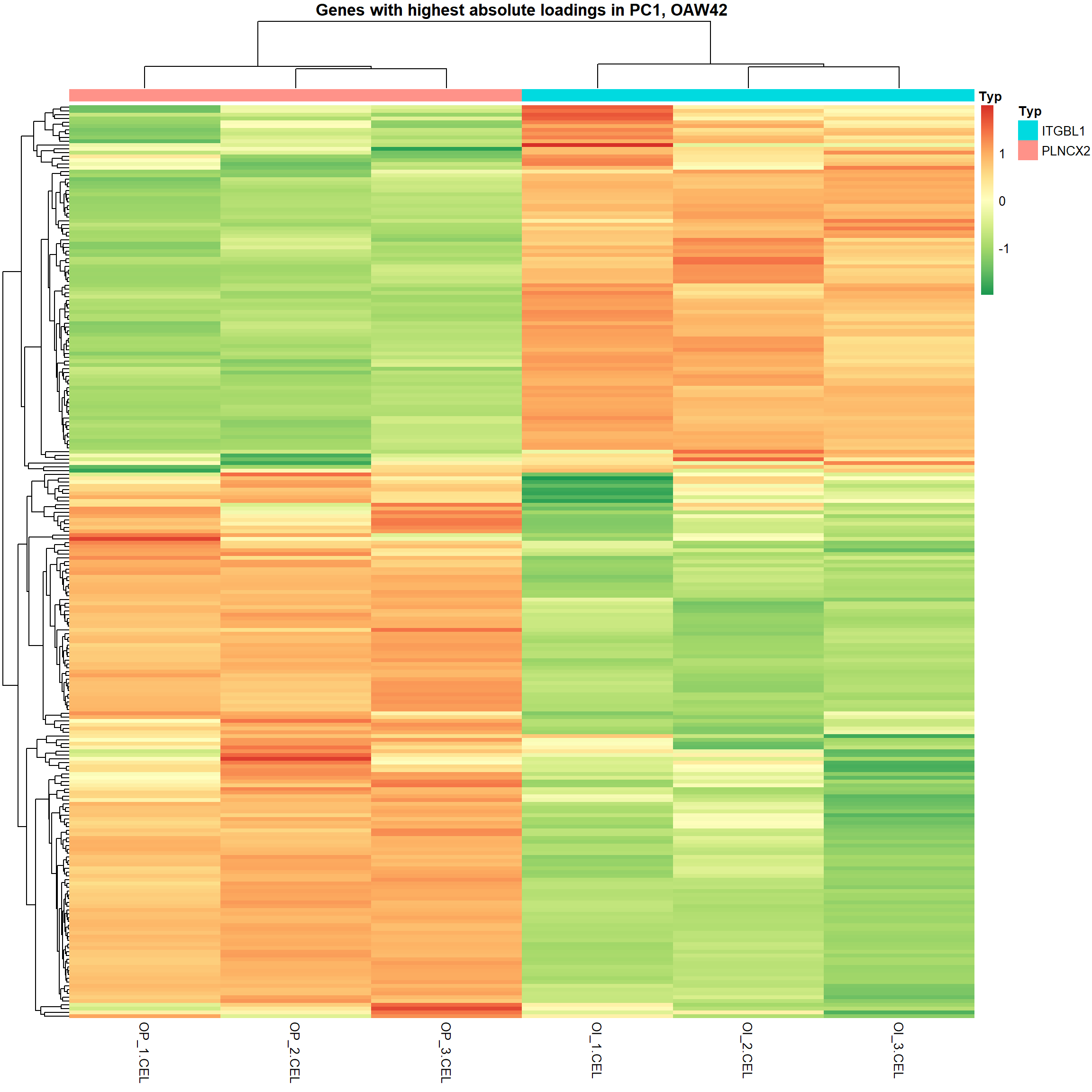


**Supplementary Material 5K. Signaling pathways affected by changed expression of the genes from PC1** (PCA performed on OAW42 cells with an empty pLNCX2 and with ITGBL1 overexpression). Out of 76 significant pathways 22 are related with ECM, integrin signaling, focal adhesion, cellular motility, etc).

**List of signaling pathways selected with PC1 gene set**

| **No** | **Pathway** | **Gene set length** | **Genes in gene set** | **Genes in gene set** | **p-value** |
| --- | --- | --- | --- | --- | --- |
| 1 | [KEGG_ECM_RECEPTOR_INTERACTION](http://software.broadinstitute.org/gsea/msigdb/cards/KEGG_ECM_RECEPTOR_INTERACTION.html) | 84 | 8 | FN1, ITGA3, ITGB4, LAMA4, RELN, SPP1, CD36, CD44 | 3,59E-06 |
| 2 | [REACTOME_SYNTHESIS_OF_BILE_ACIDS_AND_BILE_SALTS_VIA_27_HYDROXYCHOLESTEROL](http://software.broadinstitute.org/gsea/msigdb/cards/REACTOME_SYNTHESIS_OF_BILE_ACIDS_AND_BILE_SALTS_VIA_27_HYDROXYCHOLESTEROL.html) | 15 | 4 | AKR1C1, AKR1C2, AKR1C3, NR1H4 | 1,66E-05 |
| 3 | [REACTOME_SYNTHESIS_OF_BILE_ACIDS_AND_BILE_SALTS_VIA_7ALPHA_HYDROXYCHOLESTEROL](http://software.broadinstitute.org/gsea/msigdb/cards/REACTOME_SYNTHESIS_OF_BILE_ACIDS_AND_BILE_SALTS_VIA_7ALPHA_HYDROXYCHOLESTEROL.html) | 24 | 4 | AKR1C1, AKR1C2, AKR1C3, NR1H4 | 0,0001 |
| 4 | [REACTOME_LAMININ_INTERACTIONS](http://software.broadinstitute.org/gsea/msigdb/cards/REACTOME_LAMININ_INTERACTIONS.html) | 30 | 4 | ITGA3, ITGB4, LAMA4, NID1 | 0,0003 |
| 5 | [NABA_MATRISOME](http://software.broadinstitute.org/gsea/msigdb/cards/NABA_MATRISOME.html) | 1026 | 24 | CELA3A, C1QL1, FSTL1, EFEMP1, FN1, HRG, INHBB, LAMA4, MMP7, NELL2, NID1, SERPINF1, PI3, SERPINE2, P3H2, SULF2, RELN, S100A2, S100A4, S100P, CCL20, SEMA4A, SLIT3, SPP1 | 0,0003 |
| 6 | [REACTOME_SYNTHESIS_OF_BILE_ACIDS_AND_BILE_SALTS_VIA_24_HYDROXYCHOLESTEROL](http://software.broadinstitute.org/gsea/msigdb/cards/REACTOME_SYNTHESIS_OF_BILE_ACIDS_AND_BILE_SALTS_VIA_24_HYDROXYCHOLESTEROL.html) | 14 | 3 | AKR1C1, AKR1C2, AKR1C3 | 0,0004 |
| 7 | [REACTOME_SYNTHESIS_OF_BILE_ACIDS_AND_BILE_SALTS](http://software.broadinstitute.org/gsea/msigdb/cards/REACTOME_SYNTHESIS_OF_BILE_ACIDS_AND_BILE_SALTS.html) | 34 | 4 | AKR1C1, AKR1C2, AKR1C3, NR1H4 | 0,0005 |
| 8 | [PID_INTEGRIN1_PATHWAY](http://software.broadinstitute.org/gsea/msigdb/cards/PID_INTEGRIN1_PATHWAY.html) | 66 | 5 | FN1, ITGA3, LAMA4, NID1, SPP1 | 0,0007 |
| 9 | [KEGG_METABOLISM_OF_XENOBIOTICS_BY_CYTOCHROME_P450](http://software.broadinstitute.org/gsea/msigdb/cards/KEGG_METABOLISM_OF_XENOBIOTICS_BY_CYTOCHROME_P450.html) | 70 | 5 | CYP2B6, CYP2C9, AKR1C1, AKR1C2, AKR1C3 | 0,0010 |
| 10 | [REACTOME_BILE_ACID_AND_BILE_SALT_METABOLISM](http://software.broadinstitute.org/gsea/msigdb/cards/REACTOME_BILE_ACID_AND_BILE_SALT_METABOLISM.html) | 43 | 4 | AKR1C1, AKR1C2, AKR1C3, NR1H4 | 0,0012 |
| 11 | [NABA_ECM_GLYCOPROTEINS](http://software.broadinstitute.org/gsea/msigdb/cards/NABA_ECM_GLYCOPROTEINS.html) | 196 | 8 | EFEMP1, FN1, LAMA4, NELL2, NID1, RELN, SLIT3, SPP1 | 0,0014 |
| 12 | [REACTOME_EXTRACELLULAR_MATRIX_ORGANIZATION](http://software.broadinstitute.org/gsea/msigdb/cards/REACTOME_EXTRACELLULAR_MATRIX_ORGANIZATION.html) | 301 | 10 | EFEMP1, FN1, ITGA3, ITGB4, LAMA4, MMP7, NID1, P3H2, SPP1, CD44 | 0,0017 |
| 13 | [REACTOME_XENOBIOTICS](http://software.broadinstitute.org/gsea/msigdb/cards/REACTOME_XENOBIOTICS.html) | 24 | 3 | CYP2B6, CYP2C9, AHR | 0,0021 |
| 14 | [KEGG_SMALL_CELL_LUNG_CANCER](http://software.broadinstitute.org/gsea/msigdb/cards/KEGG_SMALL_CELL_LUNG_CANCER.html) | 84 | 5 | FN1, BIRC3, ITGA3, LAMA4, NFKBIA | 0,0022 |
| 15 | [REACTOME_REGULATION_OF_INSULIN_LIKE_GROWTH_FACTOR_IGF_TRANSPORT_AND_UPTAKE_BY_INSULIN_LIKE_GROWTH_FACTOR_BINDING_PROTEINS_IGFBPS](http://software.broadinstitute.org/gsea/msigdb/cards/REACTOME_REGULATION_OF_INSULIN_LIKE_GROWTH_FACTOR_IGF_TRANSPORT_AND_UPTAKE_BY_INSULIN_LIKE_GROWTH_FACTOR_BINDING_PROTEINS_IGFBPS.html) | 124 | 6 | CDH2, FSTL1, FN1, SPP1, C3, APOL1 | 0,0023 |
| 16 | [PID_TAP63_PATHWAY](http://software.broadinstitute.org/gsea/msigdb/cards/PID_TAP63_PATHWAY.html) | 54 | 4 | GPX2, ITGA3, ITGB4, S100A2 | 0,0028 |
| 17 | [PID_REELIN_PATHWAY](http://software.broadinstitute.org/gsea/msigdb/cards/PID_REELIN_PATHWAY.html) | 28 | 3 | ITGA3, MAP1B, RELN | 0,0034 |
| 18 | [KEGG_ARACHIDONIC_ACID_METABOLISM](http://software.broadinstitute.org/gsea/msigdb/cards/KEGG_ARACHIDONIC_ACID_METABOLISM.html) | 58 | 4 | CYP2B6, CYP2C9, GPX2, AKR1C3 | 0,0036 |
| 19 | [REACTOME_ARACHIDONIC_ACID_METABOLISM](http://software.broadinstitute.org/gsea/msigdb/cards/REACTOME_ARACHIDONIC_ACID_METABOLISM.html) | 59 | 4 | CYP2C9, PTGR1, GPX2, AKR1C3 | 0,0038 |
| 20 | [REACTOME_MET_ACTIVATES_PTK2_SIGNALING](http://software.broadinstitute.org/gsea/msigdb/cards/REACTOME_MET_ACTIVATES_PTK2_SIGNALING.html) | 30 | 3 | FN1, ITGA3, LAMA4 | 0,0041 |
| 21 | [PID_AVB3_OPN_PATHWAY](http://software.broadinstitute.org/gsea/msigdb/cards/PID_AVB3_OPN_PATHWAY.html) | 31 | 3 | NFKBIA, SPP1, CD44 | 0,0045 |
| 22 | [NABA_ECM_REGULATORS](http://software.broadinstitute.org/gsea/msigdb/cards/NABA_ECM_REGULATORS.html) | 238 | 8 | CELA3A, HRG, MMP7, SERPINF1, PI3, SERPINE2, P3H2, SULF2 | 0,0045 |
| 23 | [REACTOME_REGULATION_OF_COMMISSURAL_AXON_PATHFINDING_BY_SLIT_AND_ROBO](http://software.broadinstitute.org/gsea/msigdb/cards/REACTOME_REGULATION_OF_COMMISSURAL_AXON_PATHFINDING_BY_SLIT_AND_ROBO.html) | 10 | 2 | NELL2, SLIT3 | 0,0050 |
| 24 | [REACTOME_UREA_CYCLE](http://software.broadinstitute.org/gsea/msigdb/cards/REACTOME_UREA_CYCLE.html) | 10 | 2 | SLC25A15, SLC25A2 | 0,0050 |
| 25 | [REACTOME_AMINO_ACID_TRANSPORT_ACROSS_THE_PLASMA_MEMBRANE](http://software.broadinstitute.org/gsea/msigdb/cards/REACTOME_AMINO_ACID_TRANSPORT_ACROSS_THE_PLASMA_MEMBRANE.html) | 33 | 3 | SLC38A4, SLC7A2, SLC43A1 | 0,0054 |
| 26 | [REACTOME_CYTOCHROME_P450_ARRANGED_BY_SUBSTRATE_TYPE](http://software.broadinstitute.org/gsea/msigdb/cards/REACTOME_CYTOCHROME_P450_ARRANGED_BY_SUBSTRATE_TYPE.html) | 65 | 4 | CYP2B6, CYP2C9, AHR, NR1H4 | 0,0054 |
| 27 | [REACTOME_PHASE_I_FUNCTIONALIZATION_OF_COMPOUNDS](http://software.broadinstitute.org/gsea/msigdb/cards/REACTOME_PHASE_I_FUNCTIONALIZATION_OF_COMPOUNDS.html) | 105 | 5 | CYP2B6, CYP2C9, AHR, MARC1, NR1H4 | 0,0057 |
| 28 | [BIOCARTA_NUCLEARRS_PATHWAY](http://software.broadinstitute.org/gsea/msigdb/cards/BIOCARTA_NUCLEARRS_PATHWAY.html) | 34 | 3 | CYP2B6, CYP2C9, NR1H4 | 0,0058 |
| 29 | [REACTOME_TRANSPORT_OF_INORGANIC_CATIONS_ANIONS_AND_AMINO_ACIDS_OLIGOPEPTIDES](http://software.broadinstitute.org/gsea/msigdb/cards/REACTOME_TRANSPORT_OF_INORGANIC_CATIONS_ANIONS_AND_AMINO_ACIDS_OLIGOPEPTIDES.html) | 106 | 5 | SLC38A4, SLC1A1, SLC7A2, SLC12A3, SLC43A1 | 0,0059 |
| 30 | [REACTOME_CYP2E1_REACTIONS](http://software.broadinstitute.org/gsea/msigdb/cards/REACTOME_CYP2E1_REACTIONS.html) | 11 | 2 | CYP2B6, CYP2C9 | 0,0060 |
| 31 | [KEGG_FOCAL_ADHESION](http://software.broadinstitute.org/gsea/msigdb/cards/KEGG_FOCAL_ADHESION.html) | 199 | 7 | FN1, BIRC3, ITGA3, ITGB4, LAMA4, RELN, SPP1 | 0,0061 |
| 32 | [PID_HIV_NEF_PATHWAY](http://software.broadinstitute.org/gsea/msigdb/cards/PID_HIV_NEF_PATHWAY.html) | 35 | 3 | BIRC3, MAP3K5, NFKBIA | 0,0063 |
| 33 | [NABA_MATRISOME_ASSOCIATED](http://software.broadinstitute.org/gsea/msigdb/cards/NABA_MATRISOME_ASSOCIATED.html) | 751 | 16 | CELA3A, C1QL1, FSTL1, HRG, INHBB, MMP7, SERPINF1, PI3, SERPINE2, P3H2, SULF2, S100A2, S100A4, S100P, CCL20, SEMA4A | 0,0079 |
| 34 | [REACTOME_HSF1_DEPENDENT_TRANSACTIVATION](http://software.broadinstitute.org/gsea/msigdb/cards/REACTOME_HSF1_DEPENDENT_TRANSACTIVATION.html) | 38 | 3 | HSPB8, HSPA1B, DNAJB1 | 0,0080 |
| 35 | [REACTOME_DISSOLUTION_OF_FIBRIN_CLOT](http://software.broadinstitute.org/gsea/msigdb/cards/REACTOME_DISSOLUTION_OF_FIBRIN_CLOT.html) | 13 | 2 | HRG, SERPINE2 | 0,0084 |
| 36 | [REACTOME_MET_PROMOTES_CELL_MOTILITY](http://software.broadinstitute.org/gsea/msigdb/cards/REACTOME_MET_PROMOTES_CELL_MOTILITY.html) | 41 | 3 | FN1, ITGA3, LAMA4 | 0,0099 |
| 37 | [NABA_CORE_MATRISOME](http://software.broadinstitute.org/gsea/msigdb/cards/NABA_CORE_MATRISOME.html) | 275 | 8 | EFEMP1, FN1, LAMA4, NELL2, NID1, RELN, SLIT3, SPP1 | 0,0104 |
| 38 | [REACTOME_BIOLOGICAL_OXIDATIONS](http://software.broadinstitute.org/gsea/msigdb/cards/REACTOME_BIOLOGICAL_OXIDATIONS.html) | 221 | 7 | CYP2B6, CYP2C9, AHR, NNMT, MARC1, PAPSS2, NR1H4 | 0,0105 |
| 39 | [BIOCARTA_CLASSIC_PATHWAY](http://software.broadinstitute.org/gsea/msigdb/cards/BIOCARTA_CLASSIC_PATHWAY.html) | 15 | 2 | C1S, C3 | 0,0112 |
| 40 | [PID_INTEGRIN3_PATHWAY](http://software.broadinstitute.org/gsea/msigdb/cards/PID_INTEGRIN3_PATHWAY.html) | 43 | 3 | FN1, LAMA4, SPP1 | 0,0112 |
| 41 | [REACTOME_FATTY_ACID_METABOLISM](http://software.broadinstitute.org/gsea/msigdb/cards/REACTOME_FATTY_ACID_METABOLISM.html) | 177 | 6 | CYP2C9, PTGR1, GPX2, FADS1, SCD, AKR1C3 | 0,0128 |
| 42 | [REACTOME_INTEGRIN_CELL_SURFACE_INTERACTIONS](http://software.broadinstitute.org/gsea/msigdb/cards/REACTOME_INTEGRIN_CELL_SURFACE_INTERACTIONS.html) | 85 | 4 | FN1, ITGA3, SPP1, CD44 | 0,0137 |
| 43 | [REACTOME_RESPONSE_TO_ELEVATED_PLATELET_CYTOSOLIC_CA2](http://software.broadinstitute.org/gsea/msigdb/cards/REACTOME_RESPONSE_TO_ELEVATED_PLATELET_CYTOSOLIC_CA2.html) | 132 | 5 | FN1, HRG, ORM1, TMSB4X, CD36 | 0,0145 |
| 44 | [BIOCARTA_41BB_PATHWAY](http://software.broadinstitute.org/gsea/msigdb/cards/BIOCARTA_41BB_PATHWAY.html) | 18 | 2 | MAP3K5, NFKBIA | 0,0159 |
| 45 | [REACTOME_NICOTINAMIDE_SALVAGING](http://software.broadinstitute.org/gsea/msigdb/cards/REACTOME_NICOTINAMIDE_SALVAGING.html) | 19 | 2 | NNMT, PARP8 | 0,0177 |
| 46 | [REACTOME_BIOSYNTHESIS_OF_SPECIALIZED_PRORESOLVING_MEDIATORS_SPMS](http://software.broadinstitute.org/gsea/msigdb/cards/REACTOME_BIOSYNTHESIS_OF_SPECIALIZED_PRORESOLVING_MEDIATORS_SPMS.html) | 19 | 2 | CYP2C9, PTGR1 | 0,0177 |
| 47 | [REACTOME_DEGRADATION_OF_THE_EXTRACELLULAR_MATRIX](http://software.broadinstitute.org/gsea/msigdb/cards/REACTOME_DEGRADATION_OF_THE_EXTRACELLULAR_MATRIX.html) | 140 | 5 | FN1, MMP7, NID1, SPP1, CD44 | 0,0183 |
| 48 | [REACTOME_SLC_MEDIATED_TRANSMEMBRANE_TRANSPORT](http://software.broadinstitute.org/gsea/msigdb/cards/REACTOME_SLC_MEDIATED_TRANSMEMBRANE_TRANSPORT.html) | 250 | 7 | SLC44A3, SLC38A4, SLC39A10, SLC1A1, SLC7A2, SLC12A3, SLC43A1 | 0,0194 |
| 49 | [BIOCARTA_COMP_PATHWAY](http://software.broadinstitute.org/gsea/msigdb/cards/BIOCARTA_COMP_PATHWAY.html) | 20 | 2 | C1S, C3 | 0,0195 |
| 50 | [KEGG_STEROID_HORMONE_BIOSYNTHESIS](http://software.broadinstitute.org/gsea/msigdb/cards/KEGG_STEROID_HORMONE_BIOSYNTHESIS.html) | 55 | 3 | AKR1C1, AKR1C2, AKR1C3 | 0,0217 |
| 51 | [BIOCARTA_HIVNEF_PATHWAY](http://software.broadinstitute.org/gsea/msigdb/cards/BIOCARTA_HIVNEF_PATHWAY.html) | 56 | 3 | BIRC3, MAP3K5, NFKBIA | 0,0228 |
| 52 | [KEGG_BIOSYNTHESIS_OF_UNSATURATED_FATTY_ACIDS](http://software.broadinstitute.org/gsea/msigdb/cards/KEGG_BIOSYNTHESIS_OF_UNSATURATED_FATTY_ACIDS.html) | 22 | 2 | FADS1, SCD | 0,0234 |
| 53 | [REACTOME_NITRIC_OXIDE_STIMULATES_GUANYLATE_CYCLASE](http://software.broadinstitute.org/gsea/msigdb/cards/REACTOME_NITRIC_OXIDE_STIMULATES_GUANYLATE_CYCLASE.html) | 22 | 2 | PDE10A, NOS1 | 0,0234 |
| 54 | [REACTOME_HSP90_CHAPERONE_CYCLE_FOR_STEROID_HORMONE_RECEPTORS_SHR](http://software.broadinstitute.org/gsea/msigdb/cards/REACTOME_HSP90_CHAPERONE_CYCLE_FOR_STEROID_HORMONE_RECEPTORS_SHR.html) | 57 | 3 | DNAJA1, HSPA1B, DNAJB1 | 0,0238 |
| 55 | [REACTOME_METABOLISM_OF_STEROIDS](http://software.broadinstitute.org/gsea/msigdb/cards/REACTOME_METABOLISM_OF_STEROIDS.html) | 151 | 5 | AKR1C1, AKR1C2, SCD, AKR1C3, NR1H4 | 0,0244 |
| 56 | [REACTOME_NERVOUS_SYSTEM_DEVELOPMENT](http://software.broadinstitute.org/gsea/msigdb/cards/REACTOME_NERVOUS_SYSTEM_DEVELOPMENT.html) | 580 | 12 | EFNB2, EPHA7, ALCAM, NELL2, RELN, PSMB9, ADGRG6, RPS7, RPS10, SEMA4A, SLIT3, NRP2 | 0,0246 |
| 57 | [REACTOME_NON_INTEGRIN_MEMBRANE_ECM_INTERACTIONS](http://software.broadinstitute.org/gsea/msigdb/cards/REACTOME_NON_INTEGRIN_MEMBRANE_ECM_INTERACTIONS.html) | 59 | 3 | FN1, ITGB4, LAMA4 | 0,0261 |
| 58 | [REACTOME_DISEASES_OF_IMMUNE_SYSTEM](http://software.broadinstitute.org/gsea/msigdb/cards/REACTOME_DISEASES_OF_IMMUNE_SYSTEM.html) | 24 | 2 | NFKBIA, CD36 | 0,0275 |
| 59 | [REACTOME_TRAF6_MEDIATED_NF_KB_ACTIVATION](http://software.broadinstitute.org/gsea/msigdb/cards/REACTOME_TRAF6_MEDIATED_NF_KB_ACTIVATION.html) | 24 | 2 | DDX58, NFKBIA | 0,0275 |
| 60 | [PID_INTEGRIN_A9B1_PATHWAY](http://software.broadinstitute.org/gsea/msigdb/cards/PID_INTEGRIN_A9B1_PATHWAY.html) | 25 | 2 | FN1, SPP1 | 0,0297 |
| 61 | [PID_INTEGRIN_CS_PATHWAY](http://software.broadinstitute.org/gsea/msigdb/cards/PID_INTEGRIN_CS_PATHWAY.html) | 26 | 2 | ITGA3, ITGB4 | 0,0319 |
| 62 | [REACTOME_HEMOSTASIS](http://software.broadinstitute.org/gsea/msigdb/cards/REACTOME_HEMOSTASIS.html) | 678 | 13 | PDE10A, FN1, ZFPM2, HRG, ITGA3, NOS1, ORM1, SERPINE2, TMSB4X, DOCK8, CD36, CD44, DOCK4 | 0,0338 |
| 63 | [REACTOME_ENDOGENOUS_STEROLS](http://software.broadinstitute.org/gsea/msigdb/cards/REACTOME_ENDOGENOUS_STEROLS.html) | 27 | 2 | AHR, NR1H4 | 0,0343 |
| 64 | [REACTOME_SYNDECAN_INTERACTIONS](http://software.broadinstitute.org/gsea/msigdb/cards/REACTOME_SYNDECAN_INTERACTIONS.html) | 27 | 2 | FN1, ITGB4 | 0,0343 |
| 65 | [PID_LIS1_PATHWAY](http://software.broadinstitute.org/gsea/msigdb/cards/PID_LIS1_PATHWAY.html) | 28 | 2 | MAP1B, RELN | 0,0366 |
| 66 | [REACTOME_ATTENUATION_PHASE](http://software.broadinstitute.org/gsea/msigdb/cards/REACTOME_ATTENUATION_PHASE.html) | 28 | 2 | HSPA1B, DNAJB1 | 0,0366 |
| 67 | [KEGG_PPAR_SIGNALING_PATHWAY](http://software.broadinstitute.org/gsea/msigdb/cards/KEGG_PPAR_SIGNALING_PATHWAY.html) | 69 | 3 | SORBS1, SCD, CD36 | 0,0389 |
| 68 | [BIOCARTA_DEATH_PATHWAY](http://software.broadinstitute.org/gsea/msigdb/cards/BIOCARTA_DEATH_PATHWAY.html) | 29 | 2 | BIRC3, NFKBIA | 0,0391 |
| 69 | [REACTOME_SELENOAMINO_ACID_METABOLISM](http://software.broadinstitute.org/gsea/msigdb/cards/REACTOME_SELENOAMINO_ACID_METABOLISM.html) | 118 | 4 | NNMT, RPS7, RPS10, PAPSS2 | 0,0395 |
| 70 | [REACTOME_REGULATION_OF_LIPID_METABOLISM_BY_PPARALPHA](http://software.broadinstitute.org/gsea/msigdb/cards/REACTOME_REGULATION_OF_LIPID_METABOLISM_BY_PPARALPHA.html) | 119 | 4 | AHR, FADS1, CD36, NR1H4 | 0,0405 |
| 71 | [PID_CD40_PATHWAY](http://software.broadinstitute.org/gsea/msigdb/cards/PID_CD40_PATHWAY.html) | 31 | 2 | BIRC3, NFKBIA | 0,0441 |
| 72 | [REACTOME_NICOTINATE_METABOLISM](http://software.broadinstitute.org/gsea/msigdb/cards/REACTOME_NICOTINATE_METABOLISM.html) | 31 | 2 | NNMT, PARP8 | 0,0441 |
| 73 | [REACTOME_HSF1_ACTIVATION](http://software.broadinstitute.org/gsea/msigdb/cards/REACTOME_HSF1_ACTIVATION.html) | 31 | 2 | HSPA1B, DNAJB1 | 0,0441 |
| 74 | [KEGG_ARRHYTHMOGENIC_RIGHT_VENTRICULAR_CARDIOMYOPATHY_ARVC](http://software.broadinstitute.org/gsea/msigdb/cards/KEGG_ARRHYTHMOGENIC_RIGHT_VENTRICULAR_CARDIOMYOPATHY_ARVC.html) | 74 | 3 | CDH2, ITGA3, ITGB4 | 0,0463 |
| 75 | [PID_INTEGRIN_A4B1_PATHWAY](http://software.broadinstitute.org/gsea/msigdb/cards/PID_INTEGRIN_A4B1_PATHWAY.html) | 33 | 2 | FN1, SPP1 | 0,0494 |
| 76 | [REACTOME_ADHERENS_JUNCTIONS_INTERACTIONS](http://software.broadinstitute.org/gsea/msigdb/cards/REACTOME_ADHERENS_JUNCTIONS_INTERACTIONS.html) | 33 | 2 | CDH2, CADM1 | 0,0494 |

**Supplementary Material 5L. Venn diagram showing common genes in the first four pathways (A) or first four pathways highlighted in green (B) or first four pathways related with bile acids/bile salts (C)** (Signaling pathways analysis based on PC1 genes from PCA performed on OAW42-PLNCX2 and OAW42-ITGBL1 samples).

**Set 1**

**Set 2**

**Set 3**

**Set 4**

**FN1, RELN, SPP1, CD36, CD44**

**0**

**0**

**AKR1C1, AKR1C2, AKR1C3, NR1H4**

**0**

**0**

**NID1**

**0**

**0**

**0**

**0**

**ITGA3, ITGB4, LAMA4**

**0**

**0**

**0**

**A.**

**CD36, CD44**

**Set 1**

**Set 4**

**Set 5**

**Set 8**

**ITGB4**

**0**

**0**

**FN1, SPP1**

**ITGA3**

**0**

**CELA3A, C1QL1, FSTL1, EFEMP1, HRG, INHBB, MMP7, NELL2SERPINF1, PI3, SERPINE2, P3H2, SULF2, S100A2, S100A4, S100P, CCL20, SEMA4A, SLIT3**

**LAMA4**

**0**

**NID1**

**0**

**RELN**

**0**

**0**

**B.**

**Set 2**

**Set 3**

**Set 6**

**Set 7**

**AKR1C1, AKR1C2, AKR1C3**

**NR1H4**

**0**

**0**

**0**

**0**

**0**

**0**

**0**

**0**

**0**

**0**

**0**

**0**

**0**

**C.**

**Supplementary Material 5M. PCA performed on SKOV3 cells with an empty pLNCX2 (control) and with ITGBL1 overexpression.**

**PCA SKOV3-PLNCX2 vs SKOV3-ITGBL1**


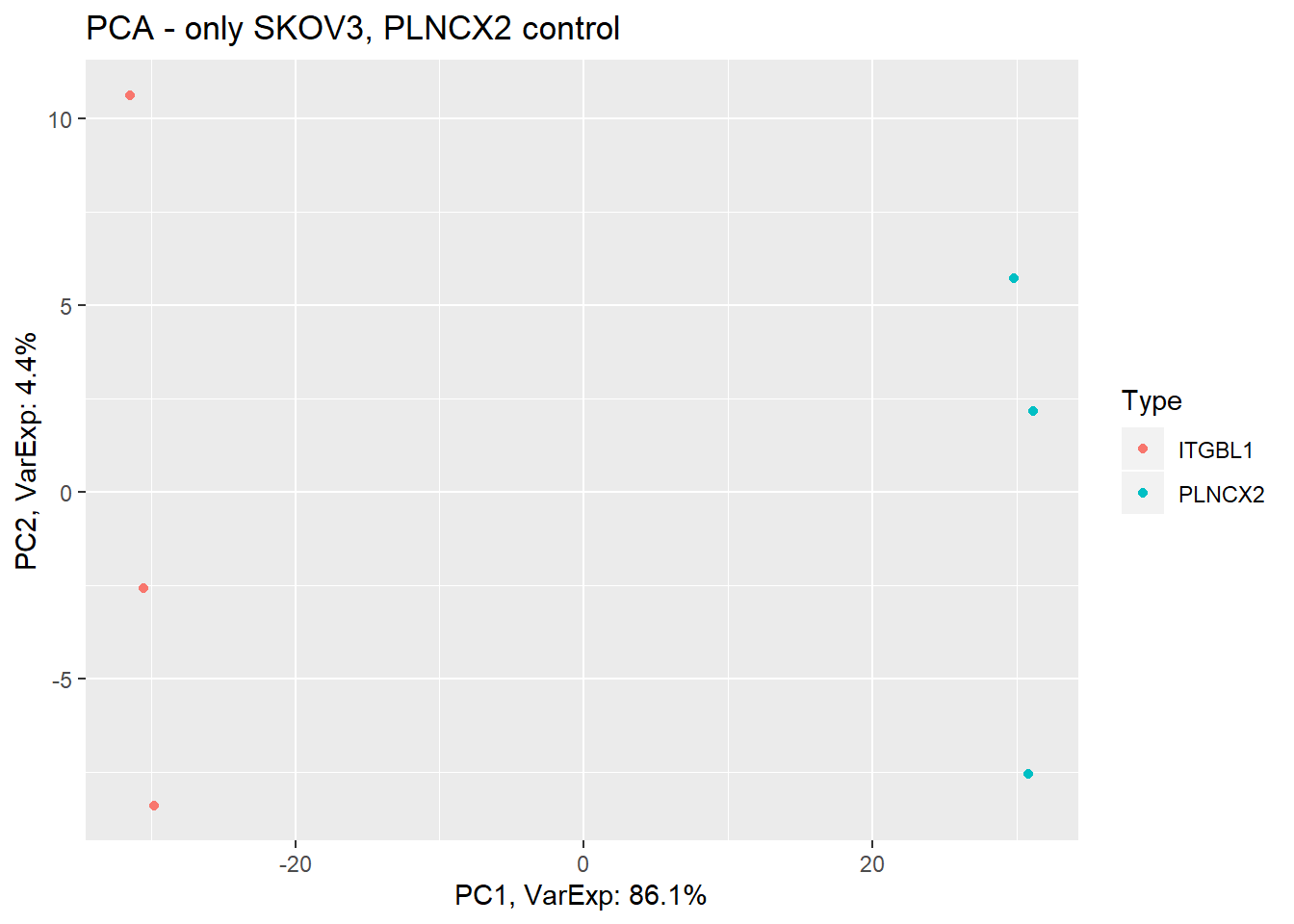


**Supplementary Material 5N. Hierarchical clustering of the samples based on the expression of genes from first principal component** (PCA performed on the SKOV3 cell line with an empty pLNCX2, and with ITGBL1 overexpression).

**PC1**


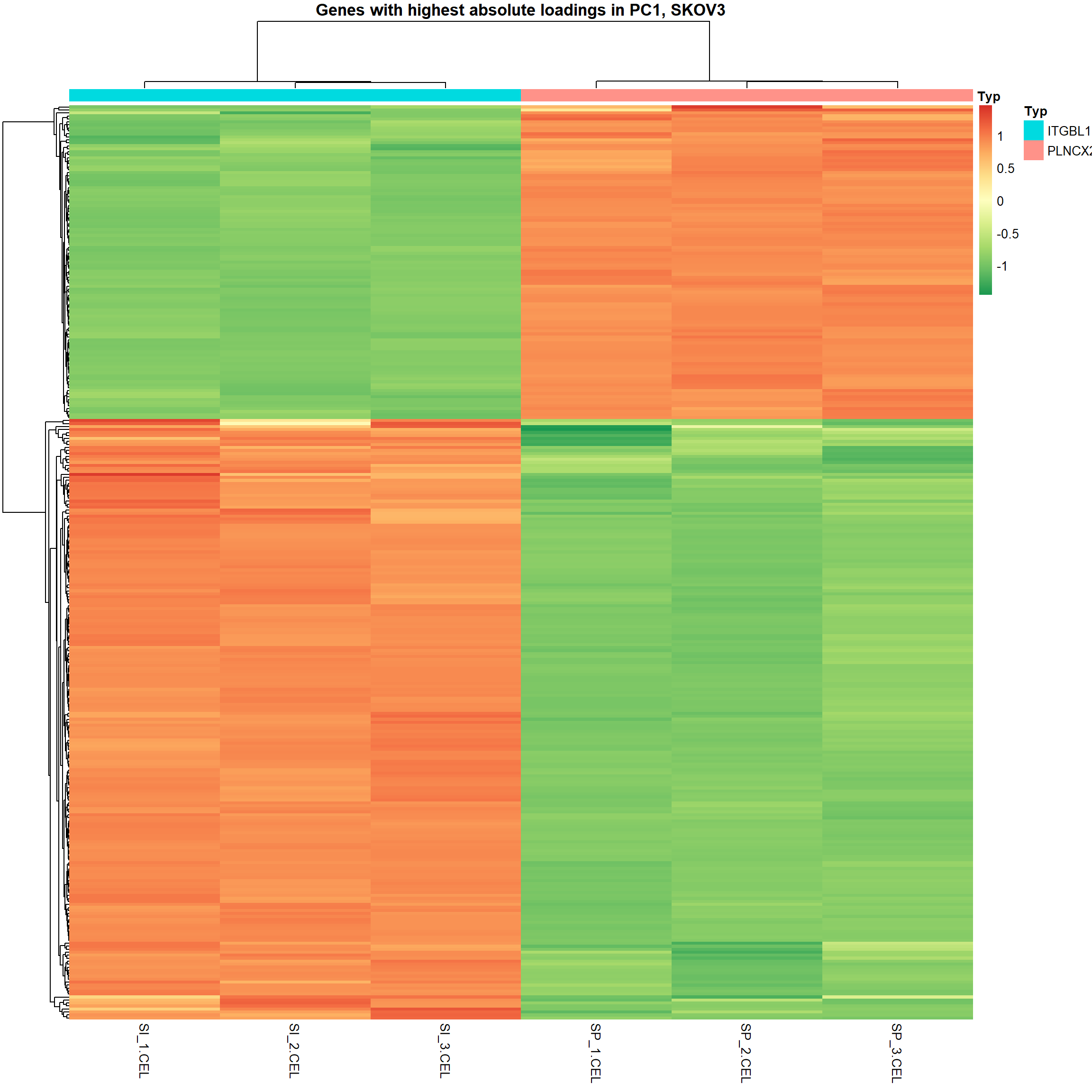


**Supplementary Material 5O. Signaling pathways affected by changed expression of the genes from PC1** (PCA performed on SKOV3 cells with an empty pLNCX2 and with ITGBL1 overexpression). Out of 146 significant pathways 44 are related with ECM, cellular communication, integrin signaling, focal adhesion, cellular motility, etc).

**List of signaling pathways selected with PC1 gene set**

| **No** | **Pathway** | **Gene set length** | **Genes in gene set** | **Genes in gene set** | **p-value** |
| --- | --- | --- | --- | --- | --- |
| 1 | [NABA_MATRISOME](http://software.broadinstitute.org/gsea/msigdb/cards/NABA_MATRISOME.html) | 1026 | 35 | GPC6, SEMA3C, ADAMTS5, FSTL1, COL8A1, VCAN, CCBE1, EREG, PCOLCE2, IL1A, AREG, C1QTNF9B, LAMB3, LOXL2, MMP1, MUC1, SERPINE1, CRIM1, SERPINB8, P3H2, SRGN, ADAMTS9, ADAMTSL3, S100P, SFRP1, SPARC, BTC, TGFBI, THBS1, VEGFA, INHBE, FNDC1, TNFSF10, ADAMTS1, GDF15 | 7,41E-07 |
| 2 | [REACTOME_EXTRACELLULAR_MATRIX_ORGANIZATION](http://software.broadinstitute.org/gsea/msigdb/cards/REACTOME_EXTRACELLULAR_MATRIX_ORGANIZATION.html) | 301 | 16 | ADAMTS5, COL8A1, VCAN, PCOLCE2, ITGA1, ITGB6, LAMB3, LOXL2, MMP1, SERPINE1, P3H2, ADAMTS9, SPARC, THBS1, ADAMTS1, CDH1 | 4,68E-06 |
| 3 | [REACTOME_TP53_REGULATES_TRANSCRIPTION_OF_GENES_INVOLVED_IN_G1_CELL_CYCLE_ARREST](http://software.broadinstitute.org/gsea/msigdb/cards/REACTOME_TP53_REGULATES_TRANSCRIPTION_OF_GENES_INVOLVED_IN_G1_CELL_CYCLE_ARREST.html) | 14 | 4 | CDK2, E2F8, CCNA1, CCNE2 | 3,14E-05 |
| 4 | [REACTOME_CELL_JUNCTION_ORGANIZATION](http://software.broadinstitute.org/gsea/msigdb/cards/REACTOME_CELL_JUNCTION_ORGANIZATION.html) | 92 | 8 | CDH2, CDH6, CLDN16, CLDN7, CADM1, KRT5, LAMB3, CDH1 | 3,96E-05 |
| 5 | [KEGG_P53_SIGNALING_PATHWAY](http://software.broadinstitute.org/gsea/msigdb/cards/KEGG_P53_SIGNALING_PATHWAY.html) | 68 | 7 | CDK2, GADD45A, SERPINE1, RRM2, THBS1, SESN2, CCNE2 | 4,08E-05 |
| 6 | [REACTOME_MITOTIC_G1_PHASE_AND_G1_S_TRANSITION](http://software.broadinstitute.org/gsea/msigdb/cards/REACTOME_MITOTIC_G1_PHASE_AND_G1_S_TRANSITION.html) | 149 | 10 | CDK2, MYBL2, MCM10, PRIM1, RRM2, SKP2, TYMS, CDC45, CCNA1, CCNE2 | 4,20E-05 |
| 7 | [PID_ERBB_NETWORK_PATHWAY](http://software.broadinstitute.org/gsea/msigdb/cards/PID_ERBB_NETWORK_PATHWAY.html) | 15 | 4 | ERBB4, EREG, AREG, BTC | 4,23E-05 |
| 8 | [REACTOME_TFAP2_AP_2_FAMILY_REGULATES_TRANSCRIPTION_OF_GROWTH_FACTORS_AND_THEIR_RECEPTORS](http://software.broadinstitute.org/gsea/msigdb/cards/REACTOME_TFAP2_AP_2_FAMILY_REGULATES_TRANSCRIPTION_OF_GROWTH_FACTORS_AND_THEIR_RECEPTORS.html) | 15 | 4 | ATAD2, KIT, VEGFA, CGB5 | 4,23E-05 |
| 9 | [NABA_MATRISOME_ASSOCIATED](http://software.broadinstitute.org/gsea/msigdb/cards/NABA_MATRISOME_ASSOCIATED.html) | 751 | 25 | GPC6, SEMA3C, ADAMTS5, FSTL1, CCBE1, EREG, IL1A, AREG, C1QTNF9B, LOXL2, MMP1, MUC1, SERPINE1, SERPINB8, P3H2, ADAMTS9, ADAMTSL3, S100P, SFRP1, BTC, VEGFA, INHBE, TNFSF10, ADAMTS1, GDF15 | 4,58E-05 |
| 10 | [REACTOME_TP53_REGULATES_TRANSCRIPTION_OF_CELL_CYCLE_GENES](http://software.broadinstitute.org/gsea/msigdb/cards/REACTOME_TP53_REGULATES_TRANSCRIPTION_OF_CELL_CYCLE_GENES.html) | 49 | 6 | CDK2, PLK2, GADD45A, E2F8, CCNA1, CCNE2 | 5,45E-05 |
| 11 | [PID_E2F_PATHWAY](http://software.broadinstitute.org/gsea/msigdb/cards/PID_E2F_PATHWAY.html) | 73 | 7 | CDK2, MYBL2, SERPINE1, RRM1, RRM2, TYMS, CCNE2 | 6,47E-05 |
| 12 | [REACTOME_TRANSCRIPTIONAL_REGULATION_BY_THE_AP_2_TFAP2_FAMILY_OF_TRANSCRIPTION_FACTORS](http://software.broadinstitute.org/gsea/msigdb/cards/REACTOME_TRANSCRIPTIONAL_REGULATION_BY_THE_AP_2_TFAP2_FAMILY_OF_TRANSCRIPTION_FACTORS.html) | 38 | 5 | ATAD2, KIT, MYBL2, VEGFA, CGB5 | 0,0002 |
| 13 | [REACTOME_DISEASES_OF_GLYCOSYLATION](http://software.broadinstitute.org/gsea/msigdb/cards/REACTOME_DISEASES_OF_GLYCOSYLATION.html) | 143 | 9 | GPC6, ADAMTS5, VCAN, GFPT1, MUC1, ADAMTS9, ADAMTSL3, THBS1, ADAMTS1 | 0,0002 |
| 14 | [REACTOME_O_GLYCOSYLATION_OF_TSR_DOMAIN_CONTAINING_PROTEINS](http://software.broadinstitute.org/gsea/msigdb/cards/REACTOME_O_GLYCOSYLATION_OF_TSR_DOMAIN_CONTAINING_PROTEINS.html) | 39 | 5 | ADAMTS5, ADAMTS9, ADAMTSL3, THBS1, ADAMTS1 | 0,0002 |
| 15 | [REACTOME_EGFR_INTERACTS_WITH_PHOSPHOLIPASE_C_GAMMA](http://software.broadinstitute.org/gsea/msigdb/cards/REACTOME_EGFR_INTERACTS_WITH_PHOSPHOLIPASE_C_GAMMA.html) | 9 | 3 | EREG, AREG, BTC | 0,0002 |
| 16 | [REACTOME_CELL_CELL_JUNCTION_ORGANIZATION](http://software.broadinstitute.org/gsea/msigdb/cards/REACTOME_CELL_CELL_JUNCTION_ORGANIZATION.html) | 65 | 6 | CDH2, CDH6, CLDN16, CLDN7, CADM1, CDH1 | 0,0003 |
| 17 | [BIOCARTA_SKP2E2F_PATHWAY](http://software.broadinstitute.org/gsea/msigdb/cards/BIOCARTA_SKP2E2F_PATHWAY.html) | 10 | 3 | CDK2, SKP2, CCNA1 | 0,0003 |
| 18 | [REACTOME_PI3K_EVENTS_IN_ERBB4_SIGNALING](http://software.broadinstitute.org/gsea/msigdb/cards/REACTOME_PI3K_EVENTS_IN_ERBB4_SIGNALING.html) | 10 | 3 | ERBB4, EREG, BTC | 0,0003 |
| 19 | [REACTOME_DISEASES_ASSOCIATED_WITH_O_GLYCOSYLATION_OF_PROTEINS](http://software.broadinstitute.org/gsea/msigdb/cards/REACTOME_DISEASES_ASSOCIATED_WITH_O_GLYCOSYLATION_OF_PROTEINS.html) | 68 | 6 | ADAMTS5, MUC1, ADAMTS9, ADAMTSL3, THBS1, ADAMTS1 | 0,0003 |
| 20 | [REACTOME_DNA_REPLICATION](http://software.broadinstitute.org/gsea/msigdb/cards/REACTOME_DNA_REPLICATION.html) | 127 | 8 | CDK2, GINS2, MCM10, PRIM1, SKP2, CDC45, CCNA1, CCNE2 | 0,0004 |
| 21 | [REACTOME_CELL_CELL_COMMUNICATION](http://software.broadinstitute.org/gsea/msigdb/cards/REACTOME_CELL_CELL_COMMUNICATION.html) | 130 | 8 | CDH2, CDH6, CLDN16, CLDN7, CADM1, KRT5, LAMB3, CDH1 | 0,0004 |
| 22 | [REACTOME_G0_AND_EARLY_G1](http://software.broadinstitute.org/gsea/msigdb/cards/REACTOME_G0_AND_EARLY_G1.html) | 27 | 4 | CDK2, MYBL2, CCNA1, CCNE2 | 0,0005 |
| 23 | [REACTOME_RESPONSE_TO_ELEVATED_PLATELET_CYTOSOLIC_CA2](http://software.broadinstitute.org/gsea/msigdb/cards/REACTOME_RESPONSE_TO_ELEVATED_PLATELET_CYTOSOLIC_CA2.html) | 132 | 8 | HSPA5, SERPINE1, GTPBP2, SRGN, SPARC, THBS1, TUBA4A, VEGFA | 0,0005 |
| 24 | [KEGG_GLUTATHIONE_METABOLISM](http://software.broadinstitute.org/gsea/msigdb/cards/KEGG_GLUTATHIONE_METABOLISM.html) | 50 | 5 | GCLC, GSTM3, IDH1, RRM1, RRM2 | 0,0006 |
| 25 | [REACTOME_DISEASES_OF_METABOLISM](http://software.broadinstitute.org/gsea/msigdb/cards/REACTOME_DISEASES_OF_METABOLISM.html) | 246 | 11 | GPC6, ADAMTS5, VCAN, GFPT1, GCLC, IDH1, MUC1, ADAMTS9, ADAMTSL3, THBS1, ADAMTS1 | 0,0006 |
| 26 | [REACTOME_G1_S_SPECIFIC_TRANSCRIPTION](http://software.broadinstitute.org/gsea/msigdb/cards/REACTOME_G1_S_SPECIFIC_TRANSCRIPTION.html) | 29 | 4 | RRM2, TYMS, CDC45, CCNA1 | 0,0006 |
| 27 | [SA_REG_CASCADE_OF_CYCLIN_EXPR](http://software.broadinstitute.org/gsea/msigdb/cards/SA_REG_CASCADE_OF_CYCLIN_EXPR.html) | 13 | 3 | CDK2, CCNA1, CCNE2 | 0,0007 |
| 28 | [REACTOME_ERBB2_ACTIVATES_PTK6_SIGNALING](http://software.broadinstitute.org/gsea/msigdb/cards/REACTOME_ERBB2_ACTIVATES_PTK6_SIGNALING.html) | 13 | 3 | ERBB4, EREG, BTC | 0,0007 |
| 29 | [REACTOME_SHC1_EVENTS_IN_ERBB4_SIGNALING](http://software.broadinstitute.org/gsea/msigdb/cards/REACTOME_SHC1_EVENTS_IN_ERBB4_SIGNALING.html) | 14 | 3 | ERBB4, EREG, BTC | 0,0008 |
| 30 | [REACTOME_SHC1_EVENTS_IN_EGFR_SIGNALING](http://software.broadinstitute.org/gsea/msigdb/cards/REACTOME_SHC1_EVENTS_IN_EGFR_SIGNALING.html) | 14 | 3 | EREG, AREG, BTC | 0,0008 |
| 31 | [REACTOME_NUCLEAR_SIGNALING_BY_ERBB4](http://software.broadinstitute.org/gsea/msigdb/cards/REACTOME_NUCLEAR_SIGNALING_BY_ERBB4.html) | 32 | 4 | ERBB4, EREG, SPARC, BTC | 0,0009 |
| 32 | [NABA_SECRETED_FACTORS](http://software.broadinstitute.org/gsea/msigdb/cards/NABA_SECRETED_FACTORS.html) | 343 | 13 | FSTL1, CCBE1, EREG, IL1A, AREG, C1QTNF9B, S100P, SFRP1, BTC, VEGFA, INHBE, TNFSF10, GDF15 | 0,0010 |
| 33 | [REACTOME_ERBB2_REGULATES_CELL_MOTILITY](http://software.broadinstitute.org/gsea/msigdb/cards/REACTOME_ERBB2_REGULATES_CELL_MOTILITY.html) | 15 | 3 | ERBB4, EREG, BTC | 0,0010 |
| 34 | [REACTOME_ACTIVATION_OF_THE_PRE_REPLICATIVE_COMPLEX](http://software.broadinstitute.org/gsea/msigdb/cards/REACTOME_ACTIVATION_OF_THE_PRE_REPLICATIVE_COMPLEX.html) | 33 | 4 | CDK2, MCM10, PRIM1, CDC45 | 0,0010 |
| 35 | [REACTOME_ADHERENS_JUNCTIONS_INTERACTIONS](http://software.broadinstitute.org/gsea/msigdb/cards/REACTOME_ADHERENS_JUNCTIONS_INTERACTIONS.html) | 33 | 4 | CDH2, CDH6, CADM1, CDH1 | 0,0010 |
| 36 | [REACTOME_GRB2_EVENTS_IN_ERBB2_SIGNALING](http://software.broadinstitute.org/gsea/msigdb/cards/REACTOME_GRB2_EVENTS_IN_ERBB2_SIGNALING.html) | 16 | 3 | ERBB4, EREG, BTC | 0,0013 |
| 37 | [REACTOME_PI3K_EVENTS_IN_ERBB2_SIGNALING](http://software.broadinstitute.org/gsea/msigdb/cards/REACTOME_PI3K_EVENTS_IN_ERBB2_SIGNALING.html) | 16 | 3 | ERBB4, EREG, BTC | 0,0013 |
| 38 | [KEGG_ONE_CARBON_POOL_BY_FOLATE](http://software.broadinstitute.org/gsea/msigdb/cards/KEGG_ONE_CARBON_POOL_BY_FOLATE.html) | 17 | 3 | MTHFD2, SHMT2, TYMS | 0,0015 |
| 39 | [REACTOME_GAB1_SIGNALOSOME](http://software.broadinstitute.org/gsea/msigdb/cards/REACTOME_GAB1_SIGNALOSOME.html) | 17 | 3 | EREG, AREG, BTC | 0,0015 |
| 40 | [REACTOME_ACTIVATION_OF_ATR_IN_RESPONSE_TO_REPLICATION_STRESS](http://software.broadinstitute.org/gsea/msigdb/cards/REACTOME_ACTIVATION_OF_ATR_IN_RESPONSE_TO_REPLICATION_STRESS.html) | 37 | 4 | CDK2, MCM10, CLSPN, CDC45 | 0,0016 |
| 41 | [REACTOME_REGULATION_OF_INSULIN_LIKE_GROWTH_FACTOR_IGF_TRANSPORT_AND_UPTAKE_BY_INSULIN_LIKE_GROWTH_FACTOR_BINDING_PROTEINS_IGFBPS](http://software.broadinstitute.org/gsea/msigdb/cards/REACTOME_REGULATION_OF_INSULIN_LIKE_GROWTH_FACTOR_IGF_TRANSPORT_AND_UPTAKE_BY_INSULIN_LIKE_GROWTH_FACTOR_BINDING_PROTEINS_IGFBPS.html) | 124 | 7 | CDH2, PRSS23, FSTL1, VCAN, AMTN, MMP1, STC2 | 0,0017 |
| 42 | [REACTOME_S_PHASE](http://software.broadinstitute.org/gsea/msigdb/cards/REACTOME_S_PHASE.html) | 161 | 8 | CDK2, ESCO2, GINS2, PRIM1, SKP2, CDC45, CCNA1, CCNE2 | 0,0018 |
| 43 | [REACTOME_G2_PHASE](http://software.broadinstitute.org/gsea/msigdb/cards/REACTOME_G2_PHASE.html) | 5 | 2 | CDK2, CCNA1 | 0,0018 |
| 44 | [PID_INTEGRIN1_PATHWAY](http://software.broadinstitute.org/gsea/msigdb/cards/PID_INTEGRIN1_PATHWAY.html) | 66 | 5 | ITGA1, LAMB3, TGFBI, THBS1, VEGFA | 0,0021 |
| 45 | [REACTOME_CELL_CYCLE_CHECKPOINTS](http://software.broadinstitute.org/gsea/msigdb/cards/REACTOME_CELL_CYCLE_CHECKPOINTS.html) | 291 | 11 | CDK2, ZWINT, CENPI, MCM10, CLSPN, CENPK, CDC45, BRIP1, CCNA1, CCNE2, EXO1 | 0,0024 |
| 46 | [KEGG_BLADDER_CANCER](http://software.broadinstitute.org/gsea/msigdb/cards/KEGG_BLADDER_CANCER.html) | 42 | 4 | MMP1, THBS1, VEGFA, CDH1 | 0,0026 |
| 47 | [REACTOME_CELL_CYCLE](http://software.broadinstitute.org/gsea/msigdb/cards/REACTOME_CELL_CYCLE.html) | 674 | 19 | CDK2, ZWINT, ESCO2, CENPI, MYBL2, GINS2, MCM10, PRIM1, RRM2, CLSPN, CENPK, SKP2, TUBA4A, TYMS, CDC45, BRIP1, CCNA1, CCNE2, EXO1 | 0,0026 |
| 48 | [REACTOME_HEMOSTASIS](http://software.broadinstitute.org/gsea/msigdb/cards/REACTOME_HEMOSTASIS.html) | 678 | 19 | CDK2, CXADR, GUCY1B1, HSPA5, ITGA1, MMP1, SERPINE1, SERPINB8, GTPBP2, SRGN, DOCK10, PSG1, PSG4, PSG5, PSG9, SPARC, THBS1, TUBA4A, VEGFA | 0,0028 |
| 49 | [REACTOME_SHC1_EVENTS_IN_ERBB2_SIGNALING](http://software.broadinstitute.org/gsea/msigdb/cards/REACTOME_SHC1_EVENTS_IN_ERBB2_SIGNALING.html) | 22 | 3 | ERBB4, EREG, BTC | 0,0033 |
| 50 | [REACTOME_DEGRADATION_OF_THE_EXTRACELLULAR_MATRIX](http://software.broadinstitute.org/gsea/msigdb/cards/REACTOME_DEGRADATION_OF_THE_EXTRACELLULAR_MATRIX.html) | 140 | 7 | ADAMTS5, COL8A1, LAMB3, MMP1, ADAMTS9, ADAMTS1, CDH1 | 0,0033 |
| 51 | [REACTOME_CONSTITUTIVE_SIGNALING_BY_ABERRANT_PI3K_IN_CANCER](http://software.broadinstitute.org/gsea/msigdb/cards/REACTOME_CONSTITUTIVE_SIGNALING_BY_ABERRANT_PI3K_IN_CANCER.html) | 75 | 5 | ERBB4, EREG, AREG, KIT, BTC | 0,0037 |
| 52 | [REACTOME_ESTROGEN_DEPENDENT_NUCLEAR_EVENTS_DOWNSTREAM_OF_ESR_MEMBRANE_SIGNALING](http://software.broadinstitute.org/gsea/msigdb/cards/REACTOME_ESTROGEN_DEPENDENT_NUCLEAR_EVENTS_DOWNSTREAM_OF_ESR_MEMBRANE_SIGNALING.html) | 24 | 3 | EREG, AREG, BTC | 0,0042 |
| 53 | [PID_AJDISS_2PATHWAY](http://software.broadinstitute.org/gsea/msigdb/cards/PID_AJDISS_2PATHWAY.html) | 48 | 4 | CDH2, GFRA1, ROBO1, CDH1 | 0,0042 |
| 54 | [REACTOME_O_LINKED_GLYCOSYLATION](http://software.broadinstitute.org/gsea/msigdb/cards/REACTOME_O_LINKED_GLYCOSYLATION.html) | 111 | 6 | ADAMTS5, MUC1, ADAMTS9, ADAMTSL3, THBS1, ADAMTS1 | 0,0043 |
| 55 | [REACTOME_SIGNALING_BY_EGFR_IN_CANCER](http://software.broadinstitute.org/gsea/msigdb/cards/REACTOME_SIGNALING_BY_EGFR_IN_CANCER.html) | 25 | 3 | EREG, AREG, BTC | 0,0047 |
| 56 | [REACTOME_CELL_CYCLE_MITOTIC](http://software.broadinstitute.org/gsea/msigdb/cards/REACTOME_CELL_CYCLE_MITOTIC.html) | 560 | 16 | CDK2, ZWINT, ESCO2, CENPI, MYBL2, GINS2, MCM10, PRIM1, RRM2, CENPK, SKP2, TUBA4A, TYMS, CDC45, CCNA1, CCNE2 | 0,0049 |
| 57 | [REACTOME_IRE1ALPHA_ACTIVATES_CHAPERONES](http://software.broadinstitute.org/gsea/msigdb/cards/REACTOME_IRE1ALPHA_ACTIVATES_CHAPERONES.html) | 50 | 4 | ERN1, GFPT1, HSPA5, WIPI1 | 0,0049 |
| 58 | [NABA_CORE_MATRISOME](http://software.broadinstitute.org/gsea/msigdb/cards/NABA_CORE_MATRISOME.html) | 275 | 10 | COL8A1, VCAN, PCOLCE2, LAMB3, CRIM1, SRGN, SPARC, TGFBI, THBS1, FNDC1 | 0,0049 |
| 59 | [REACTOME_SIGNALING_BY_ERBB2_IN_CANCER](http://software.broadinstitute.org/gsea/msigdb/cards/REACTOME_SIGNALING_BY_ERBB2_IN_CANCER.html) | 26 | 3 | ERBB4, EREG, BTC | 0,0053 |
| 60 | [NABA_ECM_REGULATORS](http://software.broadinstitute.org/gsea/msigdb/cards/NABA_ECM_REGULATORS.html) | 238 | 9 | ADAMTS5, LOXL2, MMP1, SERPINE1, SERPINB8, P3H2, ADAMTS9, ADAMTSL3, ADAMTS1 | 0,0058 |
| 61 | [REACTOME_INTEGRIN_CELL_SURFACE_INTERACTIONS](http://software.broadinstitute.org/gsea/msigdb/cards/REACTOME_INTEGRIN_CELL_SURFACE_INTERACTIONS.html) | 85 | 5 | COL8A1, ITGA1, ITGB6, THBS1, CDH1 | 0,0063 |
| 62 | [REACTOME_SIGNALING_BY_PTK6](http://software.broadinstitute.org/gsea/msigdb/cards/REACTOME_SIGNALING_BY_PTK6.html) | 54 | 4 | CDK2, ERBB4, EREG, BTC | 0,0064 |
| 63 | [BIOCARTA_G1_PATHWAY](http://software.broadinstitute.org/gsea/msigdb/cards/BIOCARTA_G1_PATHWAY.html) | 28 | 3 | CDK2, SKP2, CCNA1 | 0,0065 |
| 64 | [REACTOME_INTERCONVERSION_OF_NUCLEOTIDE_DI_AND_TRIPHOSPHATES](http://software.broadinstitute.org/gsea/msigdb/cards/REACTOME_INTERCONVERSION_OF_NUCLEOTIDE_DI_AND_TRIPHOSPHATES.html) | 29 | 3 | RRM1, RRM2, TYMS | 0,0072 |
| 65 | [REACTOME_DOWNREGULATION_OF_ERBB2_SIGNALING](http://software.broadinstitute.org/gsea/msigdb/cards/REACTOME_DOWNREGULATION_OF_ERBB2_SIGNALING.html) | 29 | 3 | ERBB4, EREG, BTC | 0,0072 |
| 66 | [KEGG_CELL_CYCLE](http://software.broadinstitute.org/gsea/msigdb/cards/KEGG_CELL_CYCLE.html) | 124 | 6 | CDK2, GADD45A, SKP2, CDC45, CCNA1, CCNE2 | 0,0074 |
| 67 | [REACTOME_ATF6_ATF6_ALPHA_ACTIVATES_CHAPERONE_GENES](http://software.broadinstitute.org/gsea/msigdb/cards/REACTOME_ATF6_ATF6_ALPHA_ACTIVATES_CHAPERONE_GENES.html) | 10 | 2 | DDIT3, HSPA5 | 0,0079 |
| 68 | [REACTOME_ORGANIC_CATION_TRANSPORT](http://software.broadinstitute.org/gsea/msigdb/cards/REACTOME_ORGANIC_CATION_TRANSPORT.html) | 10 | 2 | SLC22A15, SLC22A3 | 0,0079 |
| 69 | [REACTOME_COLLAGEN_FORMATION](http://software.broadinstitute.org/gsea/msigdb/cards/REACTOME_COLLAGEN_FORMATION.html) | 90 | 5 | COL8A1, PCOLCE2, LAMB3, LOXL2, P3H2 | 0,0080 |
| 70 | [REACTOME_SIGNALING_BY_ERBB4](http://software.broadinstitute.org/gsea/msigdb/cards/REACTOME_SIGNALING_BY_ERBB4.html) | 58 | 4 | ERBB4, EREG, SPARC, BTC | 0,0083 |
| 71 | [KEGG_GLYCINE_SERINE_AND_THREONINE_METABOLISM](http://software.broadinstitute.org/gsea/msigdb/cards/KEGG_GLYCINE_SERINE_AND_THREONINE_METABOLISM.html) | 31 | 3 | CTH, PHGDH, SHMT2 | 0,0087 |
| 72 | [REACTOME_EGFR_DOWNREGULATION](http://software.broadinstitute.org/gsea/msigdb/cards/REACTOME_EGFR_DOWNREGULATION.html) | 31 | 3 | EREG, AREG, BTC | 0,0087 |
| 73 | [REACTOME_UNFOLDED_PROTEIN_RESPONSE_UPR](http://software.broadinstitute.org/gsea/msigdb/cards/REACTOME_UNFOLDED_PROTEIN_RESPONSE_UPR.html) | 92 | 5 | DDIT3, ERN1, GFPT1, HSPA5, WIPI1 | 0,0088 |
| 74 | [REACTOME_SCF_SKP2_MEDIATED_DEGRADATION_OF_P27_P21](http://software.broadinstitute.org/gsea/msigdb/cards/REACTOME_SCF_SKP2_MEDIATED_DEGRADATION_OF_P27_P21.html) | 60 | 4 | CDK2, SKP2, CCNA1, CCNE2 | 0,0093 |
| 75 | [KEGG_ALANINE_ASPARTATE_AND_GLUTAMATE_METABOLISM](http://software.broadinstitute.org/gsea/msigdb/cards/KEGG_ALANINE_ASPARTATE_AND_GLUTAMATE_METABOLISM.html) | 32 | 3 | GFPT1, GLS, GOT1 | 0,0095 |
| 76 | [REACTOME_DNA_STRAND_ELONGATION](http://software.broadinstitute.org/gsea/msigdb/cards/REACTOME_DNA_STRAND_ELONGATION.html) | 32 | 3 | GINS2, PRIM1, CDC45 | 0,0095 |
| 77 | [REACTOME_TYPE_I_HEMIDESMOSOME_ASSEMBLY](http://software.broadinstitute.org/gsea/msigdb/cards/REACTOME_TYPE_I_HEMIDESMOSOME_ASSEMBLY.html) | 11 | 2 | KRT5, LAMB3 | 0,0095 |
| 78 | [KEGG_CELL_ADHESION_MOLECULES_CAMS](http://software.broadinstitute.org/gsea/msigdb/cards/KEGG_CELL_ADHESION_MOLECULES_CAMS.html) | 133 | 6 | CDH2, CLDN16, CLDN7, VCAN, CADM1, CDH1 | 0,0103 |
| 79 | [PID_MYC_REPRESS_PATHWAY](http://software.broadinstitute.org/gsea/msigdb/cards/PID_MYC_REPRESS_PATHWAY.html) | 63 | 4 | GADD45A, DDIT3, DKK1, SFRP1 | 0,0110 |
| 80 | [REACTOME_UNWINDING_OF_DNA](http://software.broadinstitute.org/gsea/msigdb/cards/REACTOME_UNWINDING_OF_DNA.html) | 12 | 2 | GINS2, CDC45 | 0,0113 |
| 81 | [REACTOME_GLUTATHIONE_SYNTHESIS_AND_RECYCLING](http://software.broadinstitute.org/gsea/msigdb/cards/REACTOME_GLUTATHIONE_SYNTHESIS_AND_RECYCLING.html) | 12 | 2 | GCLC, CHAC1 | 0,0113 |
| 82 | [REACTOME_ATF6_ATF6_ALPHA_ACTIVATES_CHAPERONES](http://software.broadinstitute.org/gsea/msigdb/cards/REACTOME_ATF6_ATF6_ALPHA_ACTIVATES_CHAPERONES.html) | 12 | 2 | DDIT3, HSPA5 | 0,0113 |
| 83 | [KEGG_PYRIMIDINE_METABOLISM](http://software.broadinstitute.org/gsea/msigdb/cards/KEGG_PYRIMIDINE_METABOLISM.html) | 98 | 5 | PRIM1, RRM1, RRM2, TYMS, UPP1 | 0,0114 |
| 84 | [REACTOME_PROCESSING_OF_DNA_DOUBLE_STRAND_BREAK_ENDS](http://software.broadinstitute.org/gsea/msigdb/cards/REACTOME_PROCESSING_OF_DNA_DOUBLE_STRAND_BREAK_ENDS.html) | 98 | 5 | CDK2, CLSPN, BRIP1, CCNA1, EXO1 | 0,0114 |
| 85 | [REACTOME_GLUTATHIONE_CONJUGATION](http://software.broadinstitute.org/gsea/msigdb/cards/REACTOME_GLUTATHIONE_CONJUGATION.html) | 36 | 3 | GCLC, GSTM3, CHAC1 | 0,0131 |
| 86 | [BIOCARTA_P27_PATHWAY](http://software.broadinstitute.org/gsea/msigdb/cards/BIOCARTA_P27_PATHWAY.html) | 13 | 2 | CDK2, SKP2 | 0,0133 |
| 87 | [REACTOME_DISSOLUTION_OF_FIBRIN_CLOT](http://software.broadinstitute.org/gsea/msigdb/cards/REACTOME_DISSOLUTION_OF_FIBRIN_CLOT.html) | 13 | 2 | SERPINE1, SERPINB8 | 0,0133 |
| 88 | [REACTOME_PI3K_AKT_SIGNALING_IN_CANCER](http://software.broadinstitute.org/gsea/msigdb/cards/REACTOME_PI3K_AKT_SIGNALING_IN_CANCER.html) | 102 | 5 | ERBB4, EREG, AREG, KIT, BTC | 0,0134 |
| 89 | [SIG_PIP3_SIGNALING_IN_CARDIAC_MYOCTES](http://software.broadinstitute.org/gsea/msigdb/cards/SIG_PIP3_SIGNALING_IN_CARDIAC_MYOCTES.html) | 67 | 4 | CDK2, EBP, GADD45A, ERBB4 | 0,0136 |
| 90 | [PID_ERBB4_PATHWAY](http://software.broadinstitute.org/gsea/msigdb/cards/PID_ERBB4_PATHWAY.html) | 38 | 3 | ERBB4, EREG, BTC | 0,0152 |
| 91 | [REACTOME_SMOOTH_MUSCLE_CONTRACTION](http://software.broadinstitute.org/gsea/msigdb/cards/REACTOME_SMOOTH_MUSCLE_CONTRACTION.html) | 38 | 3 | GUCY1B1, ITGA1, MYLK | 0,0152 |
| 92 | [REACTOME_PEPTIDE_HORMONE_BIOSYNTHESIS](http://software.broadinstitute.org/gsea/msigdb/cards/REACTOME_PEPTIDE_HORMONE_BIOSYNTHESIS.html) | 14 | 2 | INHBE, CGB5 | 0,0153 |
| 93 | [REACTOME_METABOLISM_OF_VITAMINS_AND_COFACTORS](http://software.broadinstitute.org/gsea/msigdb/cards/REACTOME_METABOLISM_OF_VITAMINS_AND_COFACTORS.html) | 189 | 7 | GPC6, MTHFD2, CYB5A, IDH1, ENPP1, MOCOS, SHMT2 | 0,0159 |
| 94 | [KEGG_TRYPTOPHAN_METABOLISM](http://software.broadinstitute.org/gsea/msigdb/cards/KEGG_TRYPTOPHAN_METABOLISM.html) | 40 | 3 | IDO1, ACAT2, ALDH7A1 | 0,0174 |
| 95 | [KEGG_PYRUVATE_METABOLISM](http://software.broadinstitute.org/gsea/msigdb/cards/KEGG_PYRUVATE_METABOLISM.html) | 40 | 3 | ACAT2, ALDH7A1, PCK2 | 0,0174 |
| 96 | [REACTOME_ORGANIC_CATION_ANION_ZWITTERION_TRANSPORT](http://software.broadinstitute.org/gsea/msigdb/cards/REACTOME_ORGANIC_CATION_ANION_ZWITTERION_TRANSPORT.html) | 15 | 2 | SLC22A15, SLC22A3 | 0,0176 |
| 97 | [REACTOME_NEGATIVE_REGULATION_OF_TCF_DEPENDENT_SIGNALING_BY_WNT_LIGAND_ANTAGONISTS](http://software.broadinstitute.org/gsea/msigdb/cards/REACTOME_NEGATIVE_REGULATION_OF_TCF_DEPENDENT_SIGNALING_BY_WNT_LIGAND_ANTAGONISTS.html) | 15 | 2 | DKK1, SFRP1 | 0,0176 |
| 98 | [REACTOME_RESPONSE_OF_EIF2AK1_HRI_TO_HEME_DEFICIENCY](http://software.broadinstitute.org/gsea/msigdb/cards/REACTOME_RESPONSE_OF_EIF2AK1_HRI_TO_HEME_DEFICIENCY.html) | 15 | 2 | DDIT3, CHAC1 | 0,0176 |
| 99 | [REACTOME_NEGATIVE_REGULATION_OF_THE_PI3K_AKT_NETWORK](http://software.broadinstitute.org/gsea/msigdb/cards/REACTOME_NEGATIVE_REGULATION_OF_THE_PI3K_AKT_NETWORK.html) | 110 | 5 | ERBB4, EREG, AREG, KIT, BTC | 0,0180 |
| 100 | [REACTOME_INTERLEUKIN_4_AND_INTERLEUKIN_13_SIGNALING](http://software.broadinstitute.org/gsea/msigdb/cards/REACTOME_INTERLEUKIN_4_AND_INTERLEUKIN_13_SIGNALING.html) | 111 | 5 | IL1A, LCN2, MMP1, MUC1, VEGFA | 0,0186 |
| 101 | [NABA_ECM_GLYCOPROTEINS](http://software.broadinstitute.org/gsea/msigdb/cards/NABA_ECM_GLYCOPROTEINS.html) | 196 | 7 | PCOLCE2, LAMB3, CRIM1, SPARC, TGFBI, THBS1, FNDC1 | 0,0190 |
| 102 | [BIOCARTA_P53_PATHWAY](http://software.broadinstitute.org/gsea/msigdb/cards/BIOCARTA_P53_PATHWAY.html) | 16 | 2 | CDK2, GADD45A | 0,0199 |
| 103 | [REACTOME_ECM_PROTEOGLYCANS](http://software.broadinstitute.org/gsea/msigdb/cards/REACTOME_ECM_PROTEOGLYCANS.html) | 76 | 4 | VCAN, ITGB6, SERPINE1, SPARC | 0,0207 |
| 104 | [PID_INTEGRIN3_PATHWAY](http://software.broadinstitute.org/gsea/msigdb/cards/PID_INTEGRIN3_PATHWAY.html) | 43 | 3 | TGFBI, THBS1, VEGFA | 0,0211 |
| 105 | [REACTOME_METABOLISM_OF_FOLATE_AND_PTERINES](http://software.broadinstitute.org/gsea/msigdb/cards/REACTOME_METABOLISM_OF_FOLATE_AND_PTERINES.html) | 17 | 2 | MTHFD2, SHMT2 | 0,0223 |
| 106 | [PID_P73PATHWAY](http://software.broadinstitute.org/gsea/msigdb/cards/PID_P73PATHWAY.html) | 79 | 4 | CDK2, SERPINE1, CCNE2, GDF15 | 0,0235 |
| 107 | [PID_BETA_CATENIN_NUC_PATHWAY](http://software.broadinstitute.org/gsea/msigdb/cards/PID_BETA_CATENIN_NUC_PATHWAY.html) | 80 | 4 | VCAN, DKK1, TLE4, CDH1 | 0,0245 |
| 108 | [BIOCARTA_MTA3_PATHWAY](http://software.broadinstitute.org/gsea/msigdb/cards/BIOCARTA_MTA3_PATHWAY.html) | 18 | 2 | TUBA4A, CDH1 | 0,0249 |
| 109 | [PID_A6B1_A6B4_INTEGRIN_PATHWAY](http://software.broadinstitute.org/gsea/msigdb/cards/PID_A6B1_A6B4_INTEGRIN_PATHWAY.html) | 46 | 3 | IL1A, LAMB3, CDH1 | 0,0252 |
| 110 | [PID_DELTA_NP63_PATHWAY](http://software.broadinstitute.org/gsea/msigdb/cards/PID_DELTA_NP63_PATHWAY.html) | 47 | 3 | HELLS, IL1A, KRT5 | 0,0267 |
| 111 | [REACTOME_METABOLISM_OF_WATER_SOLUBLE_VITAMINS_AND_COFACTORS](http://software.broadinstitute.org/gsea/msigdb/cards/REACTOME_METABOLISM_OF_WATER_SOLUBLE_VITAMINS_AND_COFACTORS.html) | 123 | 5 | MTHFD2, CYB5A, ENPP1, MOCOS, SHMT2 | 0,0276 |
| 112 | [REACTOME_PLATELET_ACTIVATION_SIGNALING_AND_AGGREGATION](http://software.broadinstitute.org/gsea/msigdb/cards/REACTOME_PLATELET_ACTIVATION_SIGNALING_AND_AGGREGATION.html) | 261 | 8 | HSPA5, SERPINE1, GTPBP2, SRGN, SPARC, THBS1, TUBA4A, VEGFA | 0,0284 |
| 113 | [KEGG_ECM_RECEPTOR_INTERACTION](http://software.broadinstitute.org/gsea/msigdb/cards/KEGG_ECM_RECEPTOR_INTERACTION.html) | 84 | 4 | ITGA1, ITGB6, LAMB3, THBS1 | 0,0286 |
| 114 | [KEGG_SMALL_CELL_LUNG_CANCER](http://software.broadinstitute.org/gsea/msigdb/cards/KEGG_SMALL_CELL_LUNG_CANCER.html) | 84 | 4 | CDK2, LAMB3, SKP2, CCNE2 | 0,0286 |
| 115 | [REACTOME_G2_M_CHECKPOINTS](http://software.broadinstitute.org/gsea/msigdb/cards/REACTOME_G2_M_CHECKPOINTS.html) | 168 | 6 | CDK2, MCM10, CLSPN, CDC45, BRIP1, EXO1 | 0,0289 |
| 116 | [REACTOME_DNA_REPLICATION_PRE_INITIATION](http://software.broadinstitute.org/gsea/msigdb/cards/REACTOME_DNA_REPLICATION_PRE_INITIATION.html) | 85 | 4 | CDK2, MCM10, PRIM1, CDC45 | 0,0297 |
| 117 | [REACTOME_CYCLIN_A_CDK2_ASSOCIATED_EVENTS_AT_S_PHASE_ENTRY](http://software.broadinstitute.org/gsea/msigdb/cards/REACTOME_CYCLIN_A_CDK2_ASSOCIATED_EVENTS_AT_S_PHASE_ENTRY.html) | 85 | 4 | CDK2, SKP2, CCNA1, CCNE2 | 0,0297 |
| 118 | [PID_ARF6_TRAFFICKING_PATHWAY](http://software.broadinstitute.org/gsea/msigdb/cards/PID_ARF6_TRAFFICKING_PATHWAY.html) | 49 | 3 | CPE, ITGA1, CDH1 | 0,0297 |
| 119 | [PID_FOXO_PATHWAY](http://software.broadinstitute.org/gsea/msigdb/cards/PID_FOXO_PATHWAY.html) | 49 | 3 | CDK2, GADD45A, SKP2 | 0,0297 |
| 120 | [REACTOME_TRANSCRIPTIONAL_REGULATION_BY_TP53](http://software.broadinstitute.org/gsea/msigdb/cards/REACTOME_TRANSCRIPTIONAL_REGULATION_BY_TP53.html) | 365 | 10 | CDK2, PLK2, GADD45A, GLS, E2F8, SESN2, BRIP1, CCNA1, CCNE2, EXO1 | 0,0302 |
| 121 | [REACTOME_DEFECTIVE_B4GALT7_CAUSES_EDS_PROGEROID_TYPE](http://software.broadinstitute.org/gsea/msigdb/cards/REACTOME_DEFECTIVE_B4GALT7_CAUSES_EDS_PROGEROID_TYPE.html) | 20 | 2 | GPC6, VCAN | 0,0304 |
| 122 | [REACTOME_TRANSPORT_OF_BILE_SALTS_AND_ORGANIC_ACIDS_METAL_IONS_AND_AMINE_COMPOUNDS](http://software.broadinstitute.org/gsea/msigdb/cards/REACTOME_TRANSPORT_OF_BILE_SALTS_AND_ORGANIC_ACIDS_METAL_IONS_AND_AMINE_COMPOUNDS.html) | 86 | 4 | SLC22A15, SLC44A2, SLC6A9, SLC22A3 | 0,0308 |
| 123 | [REACTOME_SIGNALING_BY_ERBB2](http://software.broadinstitute.org/gsea/msigdb/cards/REACTOME_SIGNALING_BY_ERBB2.html) | 50 | 3 | ERBB4, EREG, BTC | 0,0313 |
| 124 | [REACTOME_SIGNALING_BY_EGFR](http://software.broadinstitute.org/gsea/msigdb/cards/REACTOME_SIGNALING_BY_EGFR.html) | 50 | 3 | EREG, AREG, BTC | 0,0313 |
| 125 | [KEGG_ERBB_SIGNALING_PATHWAY](http://software.broadinstitute.org/gsea/msigdb/cards/KEGG_ERBB_SIGNALING_PATHWAY.html) | 87 | 4 | ERBB4, EREG, AREG, BTC | 0,0320 |
| 126 | [REACTOME_RHO_GTPASES_ACTIVATE_PAKS](http://software.broadinstitute.org/gsea/msigdb/cards/REACTOME_RHO_GTPASES_ACTIVATE_PAKS.html) | 21 | 2 | MYH10, MYLK | 0,0333 |
| 127 | [REACTOME_SWITCHING_OF_ORIGINS_TO_A_POST_REPLICATIVE_STATE](http://software.broadinstitute.org/gsea/msigdb/cards/REACTOME_SWITCHING_OF_ORIGINS_TO_A_POST_REPLICATIVE_STATE.html) | 90 | 4 | CDK2, SKP2, CCNA1, CCNE2 | 0,0356 |
| 128 | [KEGG_PATHWAYS_IN_CANCER](http://software.broadinstitute.org/gsea/msigdb/cards/KEGG_PATHWAYS_IN_CANCER.html) | 325 | 9 | CDK2, KIT, LAMB3, MMP1, SKP2, VEGFA, CCNA1, CCNE2, CDH1 | 0,0364 |
| 129 | [KEGG_ARGININE_AND_PROLINE_METABOLISM](http://software.broadinstitute.org/gsea/msigdb/cards/KEGG_ARGININE_AND_PROLINE_METABOLISM.html) | 54 | 3 | GLS, GOT1, ALDH7A1 | 0,0381 |
| 130 | [PID_TAP63_PATHWAY](http://software.broadinstitute.org/gsea/msigdb/cards/PID_TAP63_PATHWAY.html) | 54 | 3 | GADD45A, DHRS3, GDF15 | 0,0381 |
| 131 | [REACTOME_REGULATION_OF_TP53_ACTIVITY_THROUGH_PHOSPHORYLATION](http://software.broadinstitute.org/gsea/msigdb/cards/REACTOME_REGULATION_OF_TP53_ACTIVITY_THROUGH_PHOSPHORYLATION.html) | 92 | 4 | CDK2, BRIP1, CCNA1, EXO1 | 0,0381 |
| 132 | [KEGG_NITROGEN_METABOLISM](http://software.broadinstitute.org/gsea/msigdb/cards/KEGG_NITROGEN_METABOLISM.html) | 23 | 2 | CTH, GLS | 0,0394 |
| 133 | [KEGG_PROXIMAL_TUBULE_BICARBONATE_RECLAMATION](http://software.broadinstitute.org/gsea/msigdb/cards/KEGG_PROXIMAL_TUBULE_BICARBONATE_RECLAMATION.html) | 23 | 2 | GLS, PCK2 | 0,0394 |
| 134 | [BIOCARTA_CELLCYCLE_PATHWAY](http://software.broadinstitute.org/gsea/msigdb/cards/BIOCARTA_CELLCYCLE_PATHWAY.html) | 23 | 2 | CDK2, CCNA1 | 0,0394 |
| 135 | [PID_PRL_SIGNALING_EVENTS_PATHWAY](http://software.broadinstitute.org/gsea/msigdb/cards/PID_PRL_SIGNALING_EVENTS_PATHWAY.html) | 23 | 2 | CDK2, ITGA1 | 0,0394 |
| 136 | [REACTOME_DNA_REPAIR](http://software.broadinstitute.org/gsea/msigdb/cards/REACTOME_DNA_REPAIR.html) | 331 | 9 | CDK2, UBE2T, DTL, NEIL3, CLSPN, BRIP1, CCNA1, EXO1, PCLAF | 0,0401 |
| 137 | [PID_P53_DOWNSTREAM_PATHWAY](http://software.broadinstitute.org/gsea/msigdb/cards/PID_P53_DOWNSTREAM_PATHWAY.html) | 137 | 5 | VCAN, GADD45A, DKK1, SERPINE1, GDF15 | 0,0410 |
| 138 | [REACTOME_HOMOLOGY_DIRECTED_REPAIR](http://software.broadinstitute.org/gsea/msigdb/cards/REACTOME_HOMOLOGY_DIRECTED_REPAIR.html) | 138 | 5 | CDK2, CLSPN, BRIP1, CCNA1, EXO1 | 0,0420 |
| 139 | [KEGG_ASCORBATE_AND_ALDARATE_METABOLISM](http://software.broadinstitute.org/gsea/msigdb/cards/KEGG_ASCORBATE_AND_ALDARATE_METABOLISM.html) | 25 | 2 | ALDH7A1, UGT1A6 | 0,0459 |
| 140 | [REACTOME_CHOLESTEROL_BIOSYNTHESIS](http://software.broadinstitute.org/gsea/msigdb/cards/REACTOME_CHOLESTEROL_BIOSYNTHESIS.html) | 25 | 2 | EBP, ACAT2 | 0,0459 |
| 141 | [REACTOME_CYCLIN_A_B1_B2_ASSOCIATED_EVENTS_DURING_G2_M_TRANSITION](http://software.broadinstitute.org/gsea/msigdb/cards/REACTOME_CYCLIN_A_B1_B2_ASSOCIATED_EVENTS_DURING_G2_M_TRANSITION.html) | 25 | 2 | CDK2, CCNA1 | 0,0459 |
| 142 | [REACTOME_METABOLISM_OF_NUCLEOTIDES](http://software.broadinstitute.org/gsea/msigdb/cards/REACTOME_METABOLISM_OF_NUCLEOTIDES.html) | 99 | 4 | RRM1, RRM2, TYMS, UPP1 | 0,0477 |
| 143 | [PID_INTEGRIN_CS_PATHWAY](http://software.broadinstitute.org/gsea/msigdb/cards/PID_INTEGRIN_CS_PATHWAY.html) | 26 | 2 | ITGA1, ITGB6 | 0,0492 |
| 144 | [REACTOME_A_TETRASACCHARIDE_LINKER_SEQUENCE_IS_REQUIRED_FOR_GAG_SYNTHESIS](http://software.broadinstitute.org/gsea/msigdb/cards/REACTOME_A_TETRASACCHARIDE_LINKER_SEQUENCE_IS_REQUIRED_FOR_GAG_SYNTHESIS.html) | 26 | 2 | GPC6, VCAN | 0,0492 |
| 145 | [REACTOME_CASPASE_ACTIVATION_VIA_EXTRINSIC_APOPTOTIC_SIGNALLING_PATHWAY](http://software.broadinstitute.org/gsea/msigdb/cards/REACTOME_CASPASE_ACTIVATION_VIA_EXTRINSIC_APOPTOTIC_SIGNALLING_PATHWAY.html) | 26 | 2 | UNC5B, TNFSF10 | 0,0492 |
| 146 | [REACTOME_RESOLUTION_OF_D_LOOP_STRUCTURES_THROUGH_SYNTHESIS_DEPENDENT_STRAND_ANNEALING_SDSA](http://software.broadinstitute.org/gsea/msigdb/cards/REACTOME_RESOLUTION_OF_D_LOOP_STRUCTURES_THROUGH_SYNTHESIS_DEPENDENT_STRAND_ANNEALING_SDSA.html) | 26 | 2 | BRIP1, EXO1 | 0,0492 |

**Supplementary Material 5P. Venn diagram showing common genes in the first four signaling pathways (A) or first four signaling pathways highlighted in green (B)** (Signaling pathways analysis based on PC1 genes from PCA performed on SKOV3-PLNCX2 and SKOV3-ITGBL1 samples)

**A.**

**Set 2**

**Set 1**

**LAMB3**

**CDH1**

**Set 4**

**Set 3**

**0**

**CDH2, CDH6, CLDN16, CLDN7, CADM1, KRT5**

**CDK2, E2F8, CCNA1, CCNE2**

**GPC6, SEMA3C, FSTL1, CCBE1, IL1A, C1QTNF9B, MUC1, CRIM1, SERPINB8, SRGN, ADAMTSL3, S100P, SFRP1, TGFBI, VEGFA, INHBE, FNDC1, TNFSF10, GDF15, EREG, BTC, AREG**

**THBS1, ADAMTS1, ADAMTS5, ADAMTS9, SPARC, COL8A1, VCAN, PCOLCE2, LOXL2, MMP1, P3H2, SERPINE1**

**ITGA1, ITGB6**

**0**

**0**

**0**

**0**

**0**

**0**

**0**

**Set 1**

**Set 2**

**Set 4**

**Set 7**

**THBS1, ADAMTS1, ADAMTS5, ADAMTS9, SPARC, COL8A1, VCAN, PCOLCE2, LOXL2, MMP1, P3H2, SERPINE1**

**CDH1**

**LAMB3**

**0**

**0**

**0**

**0**

**0**

**ERBB4**

**0**

**EREG, BTC, AREG**

**0**

**B.**

**CDH2, CDH6, CLDN16, CLDN7, CADM1, KRT5**

**ITGA1, ITGB6**

**GPC6, SEMA3C, FSTL1, CCBE1, IL1A, C1QTNF9B, MUC1, CRIM1, SERPINB8, SRGN, ADAMTSL3, S100P, SFRP1, TGFBI, VEGFA, INHBE, FNDC1, TNFSF10, GDF15**
