# Supplementary material for: Evaluation of the Role of ITGBL1 in Ovarian Cancer
Source: Cancers (Basel). 2020 Sep 19;12(9):2676. doi: 10.3390/cancers12092676 (PMC7563769; doi:10.3390/cancers12092676)
Supplement: Supplementary file 1 [file cancers-12-02676-s001.zip › Suplement ITGBL1 10.09.2020/6. Supplementary Material 6. Comparison with Huang et al.docx]

**Supplementary Material 6. Comparison with Huang et al.**

Two studies analyzed gene expression pattern in the control *versus* ITGBL1-overexpressing cancer cells - our and that of Huang et al. [1]. We compared ITGBL1-overexpressing and control ovarian cancer cell lines (SKOV3 and OAW42) by Affymetrix GeneChip gene expression profiling. Huang et al. analyzed ITGBL1-overexpressing and control SMMC-7721 human hepatoma cell lines by RNA-sequencing.

Here, we present comparison of lists of differentially expressed genes found in these two studies.

[1] Wei Huang, Demin Yu, Mingjie Wang, Yue Han, Junyu Lin, Dong Wei, Jialin Cai, Bin Li, Peizhan Chen, Xinxin Zhang. ITGBL1 promotes cell migration and invasion through stimulating the TGF-β signalling pathway in hepatocellular carcinoma. Cell Prolif. 2020 Jul;53(7): e12836. doi: 10.1111/cpr.12836

**Summary:**

| **[Cortez et al.]**  **Type of comparison** | **[Cortez et al.]**  **No of genes** | **[Huang et al.]**  **No of genes** | **Common genes**  **(total)** | **Common genes**  **from those further studied by Huang et al.** |
| --- | --- | --- | --- | --- |
| SKOV3-PLNCX2 vs SKOV3-ITGBL1 | 9735 | 196 | 96 | FOS, VEGFA, **CDH2, VIM**, FOXQ1 |
| OAW42-PLNCX2 vs OAW42-ITGBL1 | 2626 | 196 | 48 | **CDH2, VIM** |

**Supplementary Material 6A. Comparison of our gene set (SKOV3-PLNCX2 vs SKOV3-ITGBL1) vs Huang et al. gene set.**

**100**

**96**

**9639**

**Huang et al. gene set**

**Our**

**gene set**

| **Rank in our gene set** | **Rank in Huang et al. gene set** | **Gene name** | **Symbol** | **Entrez ID** | **Estimated FC** | **log2 estimated FC** | **p-value** | **FDR adjusted p-value** |
| --- | --- | --- | --- | --- | --- | --- | --- | --- |
| 5 | 143 | unc-5 netrin receptor B | UNC5B | 219699 | -6,92 | -2,79 | 2,52E-16 | 1,12E-12 |
| 8 | 123 | solute carrier family 6 member 9 | SLC6A9 | 6536 | -8,56 | -3,10 | 5,31E-16 | 1,48E-12 |
| 13 | 27 | pregnancy specific beta-1-glycoprotein 5 | PSG5 | 5673 | 7,06 | 2,82 | 2,84E-15 | 4,69E-12 |
| 27 | 34 | alpha kinase 2 | ALPK2 | 115701 | 10,12 | 3,34 | 2,00E-14 | 1,60E-11 |
| 45 | 9 | ATP binding cassette subfamily G member 1 | ABCG1 | 9619 | -3,15 | -1,65 | 4,56E-14 | 2,26E-11 |
| 48 | 81 | vascular endothelial growth factor A | VEGFA | 7422 | -3,45 | -1,79 | 6,11E-14 | 2,83E-11 |
| 71 | 7 | paternally expressed 10 | PEG10 | 23089 | 8,27 | 3,05 | 1,95E-13 | 6,02E-11 |
| 77 | 78 | semaphorin 3C | SEMA3C | 10512 | 3,65 | 1,87 | 2,25E-13 | 6,51E-11 |
| 92 | 3 | pregnancy specific beta-1-glycoprotein 4 | PSG4 | 5672 | 4,18 | 2,06 | 4,00E-13 | 9,63E-11 |
| 232 | 61 | DNA damage inducible transcript 4 | DDIT4 | 54541 | -2,65 | -1,41 | 9,48E-12 | 9,10E-10 |
| 237 | 114 | ChaC glutathione specific gamma-glutamylcyclotransferase 1 | CHAC1 | 79094 | -3,60 | -1,85 | 1,02E-11 | 9,57E-10 |
| 285 | 120 | hes related family bHLH transcription factor with YRPW motif 1 | HEY1 | 23462 | -2,34 | -1,23 | 2,12E-11 | 1,66E-09 |
| 289 | 39 | DAB2, clathrin adaptor protein | DAB2 | 1601 | 2,32 | 1,22 | 2,23E-11 | 1,72E-09 |
| 296 | 5 | carboxypeptidase A4 | CPA4 | 51200 | 4,03 | 2,01 | 2,44E-11 | 1,84E-09 |
| 299 | 132 | ERBB receptor feedback inhibitor 1 | ERRFI1 | 54206 | -3,21 | -1,68 | 2,50E-11 | 1,86E-09 |
| 353 | 25 | urothelial cancer associated 1 (non-protein coding) | UCA1 | 652995 | 3,19 | 1,68 | 4,99E-11 | 3,15E-09 |
| 385 | 100 | epiregulin | EREG | 2069 | -3,20 | -1,68 | 7,79E-11 | 4,47E-09 |
| 403 | 113 | placenta specific 8 | PLAC8 | 51316 | 5,05 | 2,34 | 9,16E-11 | 5,05E-09 |
| 460 | 28 | squalene epoxidase | SQLE | 6713 | 1,87 | 0,90 | 1,66E-10 | 8,03E-09 |
| 490 | 137 | caveolin 1 | CAV1 | 857 | 1,89 | 0,92 | 2,29E-10 | 1,04E-08 |
| 499 | 88 | pregnancy specific beta-1-glycoprotein 1 | PSG1 | 5669 | 3,56 | 1,83 | 2,46E-10 | 1,10E-08 |
| 509 | 53 | N-myc downstream regulated 1 | NDRG1 | 10397 | -1,91 | -0,94 | 2,71E-10 | 1,18E-08 |
| 544 | 147 | solute carrier organic anion transporter family member 4A1 | SLCO4A1 | 28231 | -1,98 | -0,98 | 3,57E-10 | 1,46E-08 |
| 550 | 133 | lysyl oxidase | LOX | 4015 | -1,95 | -0,97 | 3,94E-10 | 1,60E-08 |
| 574 | 38 | ATP binding cassette subfamily A member 1 | ABCA1 | 19 | -1,82 | -0,87 | 5,24E-10 | 2,03E-08 |
| 633 | 32 | carboxypeptidase M | CPM | 1368 | -1,90 | -0,92 | 8,66E-10 | 3,05E-08 |
| 678 | 67 | porcupine O-acyltransferase | PORCN | 64840 | -1,57 | -0,65 | 1,26E-09 | 4,13E-08 |
| 793 | 118 | isopentenyl-diphosphate delta isomerase 1 | IDI1 | 3422 | 1,75 | 0,81 | 2,53E-09 | 7,10E-08 |
| 922 | 41 | sarcoglycan epsilon | SGCE | 8910 | 1,55 | 0,63 | 5,36E-09 | 1,29E-07 |
| 1027 | 141 | transmembrane protein 45A | TMEM45A | 55076 | 2,05 | 1,04 | 9,82E-09 | 2,13E-07 |
| 1050 | 135 | collagen type I alpha 1 chain | COL1A1 | 1277 | 1,52 | 0,61 | 1,09E-08 | 2,30E-07 |
| 1075 | 124 | LDL receptor related protein 1 | LRP1 | 4035 | 1,79 | 0,84 | 1,25E-08 | 2,59E-07 |
| 1127 | 98 | ATPase phospholipid transporting 8B1 | ATP8B1 | 5205 | 1,51 | 0,60 | 1,53E-08 | 3,02E-07 |
| 1262 | 139 | zinc finger, X-linked, duplicated B | ZXDB | 158586 | -1,75 | -0,80 | 2,45E-08 | 4,32E-07 |
| 1395 | 14 | 3-hydroxy-3-methylglutaryl-CoA synthase 1 | HMGCS1 | 3157 | 1,93 | 0,95 | 4,14E-08 | 6,61E-07 |
| 1445 | 131 | complement C1q like 1 | C1QL1 | 10882 | 1,86 | 0,90 | 4,86E-08 | 7,50E-07 |
| 1456 | 47 | keratin 16 | KRT16 | 3868 | -2,09 | -1,07 | 5,07E-08 | 7,76E-07 |
| 1570 | 176 | sterol regulatory element binding transcription factor 1 | SREBF1 | 6720 | -1,46 | -0,54 | 7,22E-08 | 1,02E-06 |
| 1585 | 154 | nexilin F-actin binding protein | NEXN | 91624 | 1,99 | 0,99 | 7,60E-08 | 1,07E-06 |
| 1606 | 163 | pyruvate carboxylase | PC | 5091 | 1,50 | 0,58 | 7,97E-08 | 1,11E-06 |
| 1610 | 65 | kinesin family member 21B | KIF21B | 23046 | -1,38 | -0,47 | 8,05E-08 | 1,11E-06 |
| 1649 | 30 | insulin like growth factor 2 mRNA binding protein 2 | IGF2BP2 | 10644 | 1,41 | 0,50 | 9,08E-08 | 1,23E-06 |
| 1748 | 55 | stearoyl-CoA desaturase | SCD | 6319 | -1,42 | -0,51 | 1,26E-07 | 1,61E-06 |
| 1864 | 22 | high mobility group AT-hook 2 | HMGA2 | 8091 | 1,40 | 0,49 | 1,86E-07 | 2,22E-06 |
| 1973 | 8 | ring finger protein 182 | RNF182 | 221687 | 1,42 | 0,51 | 2,55E-07 | 2,88E-06 |
| 2050 | 1 | integrin subunit beta like 1 | ITGBL1 | 9358 | 1,49 | 0,58 | 3,16E-07 | 3,43E-06 |
| 2141 | 80 | major histocompatibility complex, class I, B | HLA-B | 3106 | -1,56 | -0,64 | 4,12E-07 | 4,28E-06 |
| 2238 | 155 | ras homolog family member B | RHOB | 388 | 1,63 | 0,71 | 5,54E-07 | 5,52E-06 |
| 2244 | 104 | protein tyrosine phosphatase, receptor type U | PTPRU | 10076 | -1,27 | -0,35 | 5,68E-07 | 5,64E-06 |
| 2449 | 24 | calcium voltage-gated channel subunit alpha1 H | CACNA1H | 8912 | -1,36 | -0,44 | 9,30E-07 | 8,46E-06 |
| 2522 | 63 | methylsterol monooxygenase 1 | MSMO1 | 6307 | 1,56 | 0,64 | 1,14E-06 | 1,01E-05 |
| 2591 | 89 | interferon induced protein with tetratricopeptide repeats 1 | IFIT1 | 3434 | 1,54 | 0,62 | 1,33E-06 | 1,14E-05 |
| 2615 | 29 | PDZ domain containing 1 | PDZK1 | 5174 | 2,42 | 1,27 | 1,38E-06 | 1,18E-05 |
| 2663 | 94 | eva-1 homolog A, regulator of programmed cell death | EVA1A | 84141 | 1,42 | 0,50 | 1,59E-06 | 1,33E-05 |
| 2733 | 57 | plasminogen activator, urokinase | PLAU | 5328 | 1,50 | 0,59 | 1,90E-06 | 1,55E-05 |
| 2810 | 161 | plexin A3 | PLXNA3 | 55558 | -1,35 | -0,43 | 2,28E-06 | 1,81E-05 |
| 2873 | 72 | death associated protein kinase 1 | DAPK1 | 1612 | 1,38 | 0,47 | 2,65E-06 | 2,06E-05 |
| 2918 | 183 | polycystin 1, transient receptor potential channel interacting | PKD1 | 5310 | -1,28 | -0,35 | 2,94E-06 | 2,24E-05 |
| 3154 | 96 | insulin like growth factor binding protein 3 | IGFBP3 | 3486 | -1,21 | -0,28 | 5,06E-06 | 3,57E-05 |
| 3380 | 58 | family with sequence similarity 83 member A | FAM83A | 84985 | -1,28 | -0,35 | 7,68E-06 | 5,06E-05 |
| 3457 | 52 | Fos proto-oncogene, AP-1 transcription factor subunit | FOS | 2353 | -1,32 | -0,40 | 8,78E-06 | 5,66E-05 |
| 3618 | 37 | EYA transcriptional coactivator and phosphatase 4 | EYA4 | 2070 | -1,28 | -0,35 | 1,18E-05 | 7,26E-05 |
| 3650 | 4 | aryl hydrocarbon receptor nuclear translocator 2 | ARNT2 | 9915 | -1,26 | -0,33 | 1,25E-05 | 7,62E-05 |
| 4015 | 76 | matrix metallopeptidase 28 | MMP28 | 79148 | -1,31 | -0,38 | 2,39E-05 | 0,00013 |
| 4271 | 59 | tripartite motif containing 16 like | TRIM16L | 147166 | -1,29 | -0,37 | 3,70E-05 | 0,00019 |
| 4382 | 103 | protein S | PROS1 | 5627 | 1,28 | 0,36 | 4,48E-05 | 0,00023 |
| 4636 | 102 | keratin 18 | KRT18 | 3875 | -1,19 | -0,25 | 6,97E-05 | 0,00033 |
| 4961 | 84 | family with sequence similarity 83 member F | FAM83F | 113828 | -1,26 | -0,34 | 0,00012 | 0,00053 |
| 4988 | 192 | zinc finger SWIM-type containing 4 | ZSWIM4 | 65249 | -1,20 | -0,26 | 0,00012 | 0,00055 |
| 4997 | 56 | inhibin beta B subunit | INHBB | 3625 | -1,66 | -0,73 | 0,00012 | 0,00055 |
| 5023 | 150 | seizure related 6 homolog like 2 | SEZ6L2 | 26470 | -1,22 | -0,29 | 0,00013 | 0,00057 |
| 5293 | 23 | ankyrin repeat domain 1 | ANKRD1 | 27063 | 1,27 | 0,35 | 0,00019 | 0,00082 |
| 5525 | 152 | MN1 proto-oncogene, transcriptional regulator | MN1 | 4330 | 1,37 | 0,45 | 0,00026 | 0,00107 |
| 5801 | 45 | adrenomedullin 2 | ADM2 | 79924 | -1,20 | -0,26 | 0,00038 | 0,00146 |
| 6082 | 99 | glycerophosphocholine phosphodiesterase 1 | GPCPD1 | 56261 | -1,31 | -0,39 | 0,00056 | 0,00204 |
| 6392 | 166 | MAF bZIP transcription factor K | MAFK | 7975 | -1,15 | -0,20 | 0,00085 | 0,00295 |
| 6487 | 122 | interleukin 18 | IL18 | 3606 | -1,26 | -0,33 | 0,00098 | 0,00336 |
| 6537 | 172 | ferritin heavy chain 1 | FTH1 | 2495 | -1,17 | -0,23 | 0,00104 | 0,00353 |
| 6832 | 86 | Rho GTPase activating protein 29 | ARHGAP29 | 9411 | 1,18 | 0,24 | 0,00148 | 0,00483 |
| 7595 | 26 | hexokinase 2 | HK2 | 3099 | -1,16 | -0,22 | 0,00355 | 0,01042 |
| 7755 | 68 | adrenoceptor beta 2 | ADRB2 | 154 | -1,38 | -0,46 | 0,00417 | 0,01199 |
| 7760 | 83 | echinoderm microtubule associated protein like 2 | EML2 | 24139 | -1,14 | -0,18 | 0,00420 | 0,01207 |
| 7892 | 193 | transducin like enhancer of split 2 | TLE2 | 7089 | 1,12 | 0,16 | 0,00477 | 0,01347 |
| 8227 | 175 | latent transforming growth factor beta binding protein 4 | LTBP4 | 8425 | -1,13 | -0,17 | 0,00660 | 0,01788 |
| 8320 | 185 | forkhead box Q1 | FOXQ1 | 94234 | -1,23 | -0,30 | 0,00717 | 0,01919 |
| 8487 | 116 | plexin B3 | PLXNB3 | 5365 | -1,09 | -0,12 | 0,00830 | 0,02178 |
| 8644 | 130 | 6-phosphofructo-2-kinase/fructose-2,6-biphosphatase 4 | PFKFB4 | 5210 | 1,12 | 0,16 | 0,00941 | 0,02426 |
| 8855 | 148 | spire type actin nucleation factor 2 | SPIRE2 | 84501 | -1,12 | -0,17 | 0,01127 | 0,02835 |
| 8930 | 156 | semaphorin 4B | SEMA4B | 10509 | -1,08 | -0,11 | 0,01211 | 0,03020 |
| 9000 | 182 | complexin 1 | CPLX1 | 10815 | -1,14 | -0,19 | 0,01296 | 0,03207 |
| 9026 | 167 | glucosidase alpha, acid | GAA | 2548 | -1,12 | -0,17 | 0,01315 | 0,03246 |
| 9043 | 111 | multiple EGF like domains 6 | MEGF6 | 1953 | -1,10 | -0,13 | 0,01326 | 0,03267 |
| 9505 | 170 | anoctamin 8 | ANO8 | 57719 | -1,09 | -0,12 | 0,01857 | 0,04352 |
| 9614 | 162 | reticulon 4 receptor like 2 | RTN4RL2 | 349667 | -1,11 | -0,15 | 0,02008 | 0,04652 |
| 9616 | 177 | ATP binding cassette subfamily A member 2 | ABCA2 | 20 | 1,09 | 0,12 | 0,02009 | 0,04654 |
| 9665 | 101 | hexose-6-phosphate dehydrogenase/glucose 1-dehydrogenase | H6PD | 9563 | -1,17 | -0,23 | 0,02066 | 0,04761 |

**Supplementary Material 6B. Comparison of our gene set (OAW42-PLNCX2 vs OAW42-ITGBL1) vs Huang et al. gene set.**

**148**

**48**

**2578**

**Huang et al. gene set**

**Our**

**gene set**

| **Rank in our gene set** | **Rank in Huang et al. gene set** | **Gene name** | **Symbol** | **Entrez ID** | **Estimated FC** | **log2 estimated FC** | **p-value** | **FDR adjusted p-value** |
| --- | --- | --- | --- | --- | --- | --- | --- | --- |
| 12 | 55 | stearoyl-CoA desaturase | SCD | 6319 | 1,77 | 0,82 | 6,80E-10 | 1,26E-06 |
| 18 | 25 | urothelial cancer associated 1 (non-protein coding) | UCA1 | 652995 | -2,39 | -1,26 | 1,20E-09 | 1,45E-06 |
| 101 | 24 | calcium voltage-gated channel subunit alpha1 H | CACNA1H | 8912 | 1,46 | 0,54 | 1,18E-07 | 2,60E-05 |
| 109 | 8 | ring finger protein 182 | RNF182 | 221687 | 1,45 | 0,53 | 1,53E-07 | 3,11E-05 |
| 133 | 1 | integrin subunit beta like 1 | ITGBL1 | 9358 | 1,49 | 0,58 | 3,22E-07 | 5,38E-05 |
| 180 | 61 | DNA damage inducible transcript 4 | DDIT4 | 54541 | -1,41 | -0,49 | 8,62E-07 | 0,00011 |
| 184 | 98 | ATPase phospholipid transporting 8B1 | ATP8B1 | 5205 | -1,32 | -0,40 | 9,14E-07 | 0,00011 |
| 190 | 156 | semaphorin 4B | SEMA4B | 10509 | -1,26 | -0,34 | 9,48E-07 | 0,00011 |
| 206 | 22 | high mobility group AT-hook 2 | HMGA2 | 8091 | -1,32 | -0,41 | 1,20E-06 | 0,00013 |
| 205 | 53 | N-myc downstream regulated 1 | NDRG1 | 10397 | -1,35 | -0,43 | 1,21E-06 | 0,00013 |
| 214 | 134 | integrin subunit beta 4 | ITGB4 | 3691 | -1,50 | -0,59 | 1,32E-06 | 0,00014 |
| 252 | 137 | caveolin 1 | CAV1 | 857 | -1,31 | -0,39 | 2,05E-06 | 0,00018 |
| 262 | 11 | myosin ID | MYO1D | 4642 | 1,30 | 0,38 | 2,31E-06 | 0,00020 |
| 273 | 131 | complement C1q like 1 | C1QL1 | 10882 | 1,53 | 0,61 | 2,58E-06 | 0,00021 |
| 272 | 102 | keratin 18 | KRT18 | 3875 | -1,28 | -0,36 | 2,58E-06 | 0,00021 |
| 301 | 130 | 6-phosphofructo-2-kinase/fructose-2,6-biphosphatase 4 | PFKFB4 | 5210 | -1,34 | -0,43 | 3,88E-06 | 0,00029 |
| 313 | 124 | LDL receptor related protein 1 | LRP1 | 4035 | 1,39 | 0,48 | 4,41E-06 | 0,00031 |
| 328 | 159 | nuclear receptor subfamily 4 group A member 1 | NR4A1 | 3164 | 1,32 | 0,41 | 5,59E-06 | 0,00038 |
| 413 | 28 | squalene epoxidase | SQLE | 6713 | 1,24 | 0,31 | 1,35E-05 | 0,00073 |
| 442 | 147 | solute carrier organic anion transporter family member 4A1 | SLCO4A1 | 28231 | -1,28 | -0,35 | 1,66E-05 | 0,00083 |
| 527 | 23 | ankyrin repeat domain 1 | ANKRD1 | 27063 | 1,35 | 0,44 | 2,69E-05 | 0,00114 |
| 544 | 133 | lysyl oxidase | LOX | 4015 | 1,26 | 0,33 | 2,90E-05 | 0,00119 |
| 742 | 89 | interferon induced protein with tetratricopeptide repeats 1 | IFIT1 | 3434 | -1,32 | -0,40 | 9,45E-05 | 0,00284 |
| 998 | 56 | inhibin beta B subunit | INHBB | 3625 | -1,59 | -0,67 | 0,00024 | 0,00537 |
| 1033 | 193 | transducin like enhancer of split 2 | TLE2 | 7089 | 1,18 | 0,24 | 0,00027 | 0,00573 |
| 1078 | 192 | zinc finger SWIM-type containing 4 | ZSWIM4 | 65249 | 1,18 | 0,24 | 0,00031 | 0,00634 |
| 1328 | 72 | death associated protein kinase 1 | DAPK1 | 1612 | -1,19 | -0,26 | 0,00061 | 0,01016 |
| 1386 | 152 | MN1 proto-oncogene, transcriptional regulator | MN1 | 4330 | -1,32 | -0,40 | 0,00071 | 0,01148 |
| 1452 | 154 | nexilin F-actin binding protein | NEXN | 91624 | 1,29 | 0,37 | 0,00082 | 0,01265 |
| 1465 | 32 | carboxypeptidase M | CPM | 1368 | -1,17 | -0,23 | 0,00085 | 0,01285 |
| 1476 | 67 | porcupine O-acyltransferase | PORCN | 64840 | -1,12 | -0,17 | 0,00086 | 0,01290 |
| 1488 | 59 | tripartite motif containing 16 like | TRIM16L | 147166 | -1,19 | -0,25 | 0,00088 | 0,01321 |
| 1494 | 135 | collagen type I alpha 1 chain | COL1A1 | 1277 | 1,14 | 0,19 | 0,00089 | 0,01332 |
| 1505 | 101 | hexose-6-phosphate dehydrogenase/glucose 1-dehydrogenase | H6PD | 9563 | 1,30 | 0,38 | 0,00091 | 0,01350 |
| 1521 | 41 | sarcoglycan epsilon | SGCE | 8910 | 1,13 | 0,18 | 0,00094 | 0,01378 |
| 1675 | 132 | ERBB receptor feedback inhibitor 1 | ERRFI1 | 54206 | -1,22 | -0,28 | 0,00132 | 0,01752 |
| 1780 | 155 | ras homolog family member B | RHOB | 388 | -1,22 | -0,29 | 0,00163 | 0,02038 |
| 1858 | 9 | ATP binding cassette subfamily G member 1 | ABCG1 | 9619 | 1,11 | 0,15 | 0,00186 | 0,02232 |
| 1916 | 57 | plasminogen activator, urokinase | PLAU | 5328 | -1,20 | -0,26 | 0,00209 | 0,02434 |
| 1949 | 176 | sterol regulatory element binding transcription factor 1 | SREBF1 | 6720 | 1,13 | 0,18 | 0,00221 | 0,02528 |
| 2096 | 70 | angiopoietin like 4 | ANGPTL4 | 51129 | -1,19 | -0,25 | 0,00288 | 0,03065 |
| 2261 | 78 | semaphorin 3C | SEMA3C | 10512 | -1,13 | -0,18 | 0,00365 | 0,03592 |
| 2276 | 36 | PTPRF interacting protein alpha 4 | PPFIA4 | 8497 | -1,10 | -0,14 | 0,00377 | 0,03688 |
| 2416 | 27 | pregnancy specific beta-1-glycoprotein 5 | PSG5 | 5673 | 1,13 | 0,18 | 0,00458 | 0,04219 |
| 2510 | 119 | tensin 1 | TNS1 | 7145 | -1,12 | -0,16 | 0,00514 | 0,04561 |
| 2532 | 150 | seizure related 6 homolog like 2 | SEZ6L2 | 26470 | 1,13 | 0,17 | 0,00529 | 0,04655 |
| 2539 | 112 | aldehyde dehydrogenase 2 family member | ALDH2 | 217 | 1,16 | 0,22 | 0,00536 | 0,04701 |
| 2605 | 99 | glycerophosphocholine phosphodiesterase 1 | GPCPD1 | 56261 | -1,21 | -0,28 | 0,00572 | 0,04888 |
