# Supplementary material for: Evaluation of the Role of ITGBL1 in Ovarian Cancer
Source: Cancers (Basel). 2020 Sep 19;12(9):2676. doi: 10.3390/cancers12092676 (PMC7563769; doi:10.3390/cancers12092676)
Supplement: Supplementary file 1 [file cancers-12-02676-s001.zip › Suplement ITGBL1 10.09.2020/7. Supplementary Material 7. Antibodies.docx]

**Supplementary Material 7. Antibodies used in other ITGBL1-related studies**

**Ovarian cancer (1) Sun et al, 2016** Extracellular matrix protein ITGBL1 promotes ovarian cancer cell migration and adhesion through Wnt/PCP signaling and FAK/SRC pathway. Biomed Pharmacother. <https://doi.org/10.1016/j.biopha.2016.03.053>

| **WB Ab: Proteintech**, no cat# indicated, (1:1000). **Our remarks:** Proteintech has in its catalogue Ab (17484-1-AP) raised against ITGBL1-GST fusion protein cat# Ag11521 but is currently not selling this Ab. Sun et al, 2016 is referred in the references for this Ab: <https://www.ptglab.com/Products/ITGBL1-Antibody-17484-1-AP.htm#publications> | | | | **Our remarks:** No information about the Ab used for IHC, no HE-stained tissue sections shown, control tissues not listed. |
| --- | --- | --- | --- | --- |
| ITGBL1 detection in wt OC cell lines (cell lysates) | ITGBL1 detection in knocked-down OC cell lines (cell lysates) | ITGBL1 detection in knocked-down OC cell lines (cell lysates) | WB detection of ITGBL1-GST fusion protein (Proteintech Cat#: Ag11521). **Note:** Band size wrongly denoted (manufacturer declared size - 64 kDa) | **Authors:** we found that expression of ITGBL1 is elevated in malignant tissues compared to normal tissues.  **Our remarks:** In our pathologist’s opinion samples described as “normal” resemble cancer tissue (large, abnormal nuclei, etc.). |
| **Our remarks:**No size marker shown, no size indicated for wt ITGBL1 | | |  |  |
|  |  |  |  |  |

**Ovarian cancer (2) Song et al, 2020** Upregulation of ITGBL1 predicts poor prognosis and promotes chemoresistance in ovarian cancer. Cancer Biomark. DOI 10.3233/CBM-190460

| **WB Ab:** AV42196, Sigma-Aldrich, St. Louis, MO, USA. **Our remarks:** Ab dilution not reported, no size marker visible on the blot; ITGBL1 is detected in cell lysates. We analyzed ES2 and SKOV3 with HPA005676 Ab and were able to detect ITGBL1 only in culture media, while not in cell lysates. | | | **IHC Ab:** ab251678, Abcam Inc, USA (1:200). **Our remarks:** Abcam ab251678 dedicated to IHC is identical to **HPA005676 (Sigma).** HE-stained tissue sections not shown, control tissues not listed. |
| --- | --- | --- | --- |
| ITGBL1 detection in wt OC cell lines (cell lysates) | ITGBL1 detection in knocked-down OC cell lines | ITGBL1 detection in knocked-down OC cell lines (cell lysates) | **Authors conclusion:** ITGBL1 expression was significantly upregulated in ovarian cancer tissues compared to that in adjacent non-cancer tissues and high expression of ITGBL1 was significantly associated with lymph node invasion and advanced FIGO stage.  **Our remarks:** In adjacent non-cancer tissue inclusion cyst is visible with strongly stained epithelium, which is a counterpart for epithelial ovarian cancer |
|  |  |  |  |

**Colorectal cancer (3) Qiu et al, 2018** ITGBL1 promotes migration, invasion and predicts a poor prognosis in colorectal cancer. Biomed Pharmacother. <https://doi.org/10.1016/j.biopha.2018.05.033>

| **WB Ab:** Sigma-Aldrich, USA, no cat#, (1:1000). **Our remarks:** Sigma is selling two Ab dedicated to WB **(AV42196** and **AV42197)**, raised against two distinct epitopes | | **IHC Ab: Absin China** (1:50), no cat#. **Our remarks:** we were unable to find company‘s web site. HE-stained tissue sections not shown. Control tissues not listed. |
| --- | --- | --- |
| ITGBL1 detection in wt CRC cell lines | ITGBL1 detection in knocked-down CRC cell lines | **Authors conclusion:** ITGBL1 expression in CRC tissues was more frequent than in adjacent normal tissues. |
|  |  |  |
| **Our remarks:** No size marker on the blot, no ITGBL1 band size indicated | |  |

**Gastric cancer (4) Li et al, 2017**  ITGBL1 predicts a poor prognosis and correlates with EMT phenotype in gastric cancer. J Cancer doi: 10.7150/jca.20900

| **WB Ab:** Abnova, US, no cat#, (1:500). **Our remarks:** Abnova produces one Ab (PAB20310) recommended to WB and IHC | **IHC Ab:** Abnova, US, no cat#, (1:200). **Our remarks:** Abnova produces one Ab (PAB20310) recommended to WB and IHC. HE-stained tissue sections not shown. Control tissues not listed. |
| --- | --- |
| WB detection of ITGBL1 in cancer tissue and tumor adjacent normal tissue. **Our remarks:** No size marker, no ITGBL1 band size indicated | **Authors conclusion:** ITGBL1 expression was significantly up-regulated in GC compared with normal controls. Elevated ITGBL1 expression was positively correlated with node-metastasis and distant metastasis stage. |
|  |  |

**Lung cancer (5) Gan et al., 2015** DOI 10.1007/s13277-015-3919-8

| **WB Ab:**Sigma-Aldrich, no cat#, (1:1000). **Our remarks:** Sigma is selling two Ab dedicated to WB, produced against two different epitopes: **AV42196** and **AV42197** | |
| --- | --- |
| ITGBL1 detection in wt lung cancer cell lines | ITGBL1 detection in knocked-down cell lines |
|  |  |
| **Our remarks:** No size marker, no ITGBL1 band size indicated | |

| **Pulmonary fibrosis (6) Song et al, 2019** lncITPF Promotes Pulmonary Fibrosis by Targeting hnRNP-L Depending on Its Host Gene ITGBL1 <https://doi.org/10.1016/j.ymthe.2018.08.026> | |  | **Liver fibrosis (7) Wang et al, 2017**  DOI: 10.1038/srep43446 |
| --- | --- | --- | --- |
| **WB Ab:** Abcam, Hong Kong, China; no cat#, no dilution.  **Our remarks:** Abcam is currently selling one Ab dedicated to IHC and WB: ab244416, previously Abcam offered also av93592 (WB, ELISA) | |  | **WB Ab:** Abcam, Cambridge, Massachusetts, USA; no cat#, no dilution indicated.  **Our remarks:** Abcam is selling one Ab dedicated to IHC and WB: ab244416, previously Abcam offered also av93592 (WB, ELISA) |
| WB detection of ITGBL1 in MRC-5 cells | |  | WB detection of ITGBL1 in Huh7 cell line |
|  |  |  |  |
| **Our remarks:**No size marker on the blot. Size indicated as 88 kDa (estimated ITGBL1 size is 54 kDa) | |  | **Our remarks:** No size marker on the blot. |

**Hepatocellular carcinoma (9) Huang et al, 2020** doi:10.1111/cpr.12836.

| **WB Ab:** Abcam; ab93592; 1:1000. **Our remarks:** Abcam is currently not selling this Ab | | |  | **IHC Ab:** Novus; NBP1-82473; 1:100 |
| --- | --- | --- | --- | --- |
| WB detection of ITGBL1 in liver tumor and adjacent normal tissue | WB detection of ITGBL1 in liver cancer cell lines | WB detection of ITGBL1 in liver cancer cell lines with overexpression | WB detection of ITGBL1 in knocked-down cell line | HE-stained tissue sections not shown, only this quality picture available (preprint) |
|  |  |  |  |  |
| **Our remarks:**No size marker on the blots, no ITGBL1 band size indicated | | | |  |

**Prostate cancer (10) Li et al, 2019** DOI: [10.2147/OTT.S200082](https://doi.org/10.2147/ott.s200082)

| **WB Ab:** in WB methodology, Proteintech Cat# Ag11521 (referring to ITGBL1-GST fusion protein) is indicated wrongly as an anti-ITGBL1 Ab.  **Our remarks:** Possibly, Authors used the same Ab as Sun et al (2016) (cat# 17484-1-AP; currently not sold), however their work is not listed among references for this Ab at Proteintech web site | | | **IHC Ab:** not defined in Methods section, HE-stained tissue sections not shown |
| --- | --- | --- | --- |
| WB detection of ITGBL1 in prostate tumor and adjacent normal tissue | WB detection of ITGBL1 in prostate cancer cell lines | WB detection of ITGBL1 in prostate cancer cell lines with overexpression and knock-down | **Authors conclusions:** ITGBL1 expression level associated with lymph node metastases, with Gleason score, and stage.  **Our remarks:** In opinion of our pathologist, image described as non-lymph nodes metastatic prostate cancer (non-LNM) resembles normal prostate tissue (small, regular nuclei, normal nucleus-to-cytoplasm ratio, etc.) |
|  |  |  |  |
| **Our remarks:** No size marker on the blots, no ITGBL1 band size indicated | | |  |

**Breast cancer (11) Li et al., 2015** DOI: 10.1158/0008-5472.CAN-15-0240

| Wb Ab - GenWay Biotech, San Diego, CA, USA (no cat #); WB methodology not described  Our note: GenWay Biotech offers one anty-ITGBL1 Ab <https://www.genwaybio.com/itgbl1-integrin-beta-like-1-with-egf-like-repeat-domains>), |
| --- |
| WB detection of ITGBL1 in breast cancer cell lines with overexpression and knock-down.  **Our remarks:**No size marker on the blots, no ITGBL1 band size indicated |
|  |

**Cartilage and Arthritis (12) Song et al., 2018** doi: 10.1126/scitranslmed.aam7486

| **IHC Ab:** Sigma, no cat#, no dilution indicated. **Our remark:** Sigma is selling one Ab dedicated to IHC - HPA005676 | | |
| --- | --- | --- |
| ITGBL1 detection in mouse cartilage, normal and with induced osteoarthritis | | |
|  |  |  |
